# MZF-1/Elk-1 interaction domain as therapeutic target for protein kinase Cα-based triple-negative breast cancer cells

**DOI:** 10.18632/oncotarget.11337

**Published:** 2016-08-17

**Authors:** Chia-Jen Lee, Li-Sung Hsu, Chia-Herng Yue, Ho Lin, Yung-Wei Chiu, Yu-Yu Lin, Chih-Yang Huang, Mien-Chie Hung, Jer-Yuh Liu

**Affiliations:** ^1^ Center for Molecular Medicine, China Medical University Hospital, Taichung 40402, Taiwan; ^2^ Institute of Biochemistry, Microbiology and Immunology, Medical College, Chung-Shan Medical University, Taichung 40201, Taiwan; ^3^ Clinical Laboratory, Chung Shan Medical University Hospital, Taichung 40201, Taiwan; ^4^ Department of Surgery, Tungs' Taichung Metro Harbor Hospital, Taichung 435, Taiwan; ^5^ Department of Life Science, National Chung Hsing University, Taichung 402, Taiwan; ^6^ Emergency Department and Center of Hyperbaric Oxygen Therapy, Tungs' Taichung Metro Harbor Hospital, Taichung 435, Taiwan; ^7^ Graduate Institute of Cancer Biology, China Medical University, Taichung 40402, Taiwan; ^8^ Graduate Institute of Chinese Medical Science, School of Chinese Medicine, China Medical University, Taichung 40402, Taiwan; ^9^ Graduate Institute of Basic Medical Science, China Medical University, Taichung 40402, Taiwan; ^10^ Department of Health and Nutrition Biotechnology, Asia University, Taichung 41354, Taiwan; ^11^ Department of Molecular and Cellular Oncology, The University of Texas MD Anderson Cancer Center, Houston, Texas 77030, USA

**Keywords:** MZF-1, Elk-1, PKCα, triple-negative breast cancer cells

## Abstract

Recent reports demonstrate that the expression of protein kinase C alpha (PKCα) in triple-negative breast cancer (TNBC) correlates with decreased survival outcomes. However, off-target effects of targeting PKCα and limited understanding of the signaling mechanisms upstream of PKCα have hampered previous efforts to manipulate this ubiquitous gene. This study shows that the expression of both myeloid zinc finger 1 (MZF-1) and Ets-like protein-1 (Elk-1) correlates with PKCα expression in TNBC. We found that the acidic domain of MZF-1 and the heparin-binding domain of Elk-1 facilitate the heterodimeric interaction between the two genes before the complex formation binds to the PKCα promoter. Blocking the formation of the heterodimer by transfection of MZF-1_60–72_ or Elk-1_145–157_ peptide fragments at the MZF-1 / Elk-1 interface decreases DNA-binding activity of the MZF-1 / Elk-1 complex at the PKCα promoter. Subsequently, PKCα expression, migration, tumorigenicity, and the epithelial–mesenchymal transition potential of TNBC cells decrease. These subsequent effects are reversed by transfection with full-length PKCα, confirming that the MZF-1/Elk-1 heterodimer is a mediator of PKCα in TNBC cells. These data suggest that the next therapeutic strategy in treating PKCα-related cancer will be developed from blocking MZF-1/Elk-1 interaction through their binding domain.

## INTRODUCTION

Triple-negative breast cancers (TNBCs) comprise most of breast cancer phenotypes that are difficult to treat because they do not express estrogen receptor (ER), progesterone receptor (PR), and HER2 genes [1]. Treatment for TNBC currently involves conventional chemotherapy; however, relapse leading to poor outcome occurs frequently because of high rates of metastasis and general inaccuracy of chemotherapy [2, 3]. The presence of cancer stem cells (CSCs) or tumor-initiating cells (TICs) can account for treatment failure and TNBC recurrence since breast TICs (BTICs) can reinitiate tumor growth after treatment [4–6] and are responsible for tumor initiation, progression, and drug resistance [7, 8]. Since the identification of TNBC/BTIC, prognosis accuracy has been improved; however, clinical trials have yet to improve treatment results.

Tam et al. [9] showed that protein kinase C alpha (PKCα) is a central regulatory node in cells with EMT-caused breast CSCs. Hsu et al. [10] revealed that PKCα is associated with TNBC/BTICs in both cell lines and tumor samples; additionally, the expression of PKCα in TNBC is correlated with decreased survival outcomes. These findings suggest that PKCα is a unique prognostic marker and an achievable therapeutic target for TNBC. However, although the development of therapeutic agents targeting PKCα has been the focus of several laboratories [11], targeting PKCα often leads to off-target effects. Better understanding about the signaling mechanism upstream is required.

Several mechanisms contributing to PKCα expression have been investigated. These mechanisms include the shift in signaling from the epidermal growth factor receptor to the platelet-derived growth factor receptor during progression from non-stem cells to CSCs [9]; epithelial–mesenchymal transition (EMT) associated with PKCα overexpression and activation in two genetic models of breast cancer cells [12]; and activation of erythroblastic leukemia viral oncogene homolog 2 (ErbB2) [13].

In the present study, we specifically considered the transcription factors Ets-like protein-1 (Elk-1) and myeloid zinc finger-1 (MZF-1), which regulate PKCα expression in cancer cells [14–16]. In addition, we found that the Elk-1/MZF-1 and PKCα expression is also correlated with the potential of cell migration and invasion in TNBC cells [17]. We found that the formation of MZF-1/Elk-1 heterodimer can modulate PKCα expression. Overall, this study explored a peptide-based strategy inhibiting EMT and tumorigenesis in TNBC cells.

## RESULTS

### PKCα expression correlates with MZF-1/Elk-1 in breast cancer and TNBC

**Figure 1:**
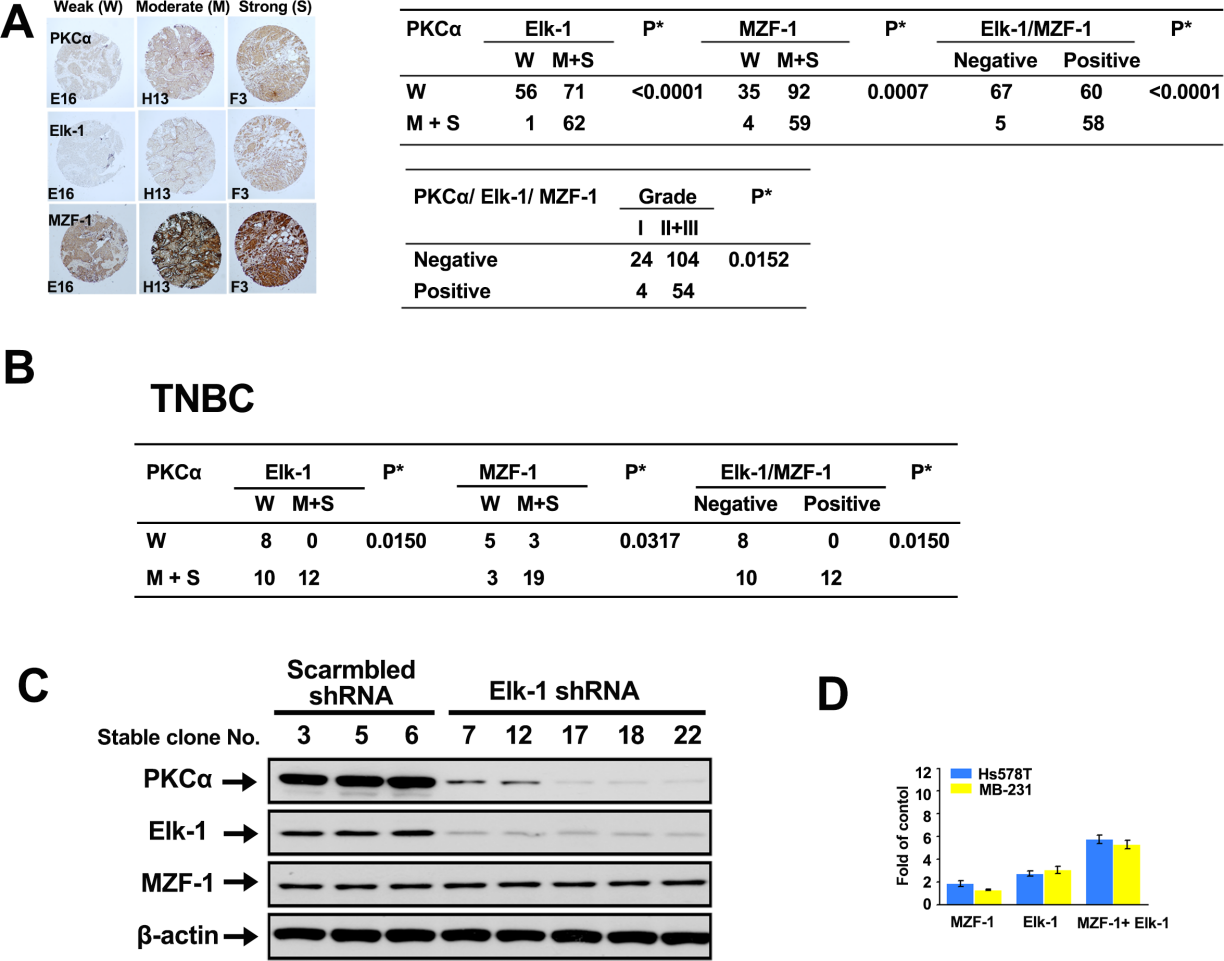
Correlations between PKCα expression and Elk-1/MZF-1 expression in breast cancer (**A**) Immunohistochemical analyses and correlations of PKCα and Elk-1/MZF-1 expression in human breast cancer. The left panel shows representative staining results for samples scored by visual assessment as “weak,” “moderate,” or “strong” according to staining intensity. The right panel depicts the numbers of each group classified based on PKCα, Elk-1, or MZF-1 staining intensity or grade. Moderate or strong expression of the genes of interest was given a positive rating, otherwise, a negative rating. Clinical characteristic grades of I, II, and III were obtained from US Biomax Inc. *P < 0.05, Pearson's chi-squared test. (**B**) Correlations of PKCα and Elk-1/MZF-1 expression by immunohistochemical analyses in human TNBC. (**C**) Effects of Elk-1 knockdown on PKCα expression in TNBC cell lines. Immunoblotting analysis of the expression levels of PKCα, Elk-1, and MZF-1 in cells transfected with Elk-1 shRNA. β-actin was used as an internal control. The shRNA Elk-1-expressing plasmid vector was constructed using the pcDNA-HU6 vector (given by Dr. J. Tsai Chang, Institute of Toxicology, College of Medicine, Chung Shan Medical University, Taichung, Taiwan) as the vector backbone. The shRNA Elk-1 duplex sequence obtained from the human Elk-1 genes (GenBank, NCBI) was designed using the BLOCK-iT™ RNAi Designer software available at http://www.invitrogen.com. The sequence corresponded to the coding regions relative to the first nucleotide of the start codon. Stable clones were selected with geneticin (G418; 600 μg/ml) at 37 °C for 4 weeks. (**D**) Luciferase activity of TNBC cell lines co-transfected with 2.5 μg of MZF-1 or Elk-1 or MZF-1/Elk-1 expression vectors. Transcriptional activity is expressed as the fold change in induction compared with the control group (*n* = 3).

To determine whether the clinical relevance of the correlation between PKCα and Elk-1 and/or MZF-1 exists in breast cancer, we examined the expression of these proteins in tissue arrays by immunohistochemical (IHC) staining. We observed a positive correlation between moderate-to-strong PKCα and either Elk-1 and/or MZF-1 staining in breast cancer (Figure 1A). The moderate-to-strong staining of PKCα/Elk-1/MZF-1 occurred most frequently in grades 2 and 3. We also detected the same proteins in the tissue array of TNBC, in which correlations between moderate-to-strong PKCα and either Elk-1 and/or MZF-1 staining were also observed (Figure 1B). We then validated these results in siRNA Elk-1 knockdown assay, which showed decreased PKCα protein expression in TNBC MB-231 cells (Figure 1C). We evaluated PKCαpromoter containing a putative binding site for MZF-1/Elk-1 via luciferase reporter assay in TNBC MDA-MB-231/Hs578T cells (Figure 1D), which showed more than one fold increase in the transcriptional activities by Elk-1 or/and MZF-1, supporting the role of Elk-1/MZF-1 in regulating PKCα expression in TNBC.

### MZF-1/Elk-1 complex binds to the promoter region of PKCα in TNBC cells

**Figure 2:**
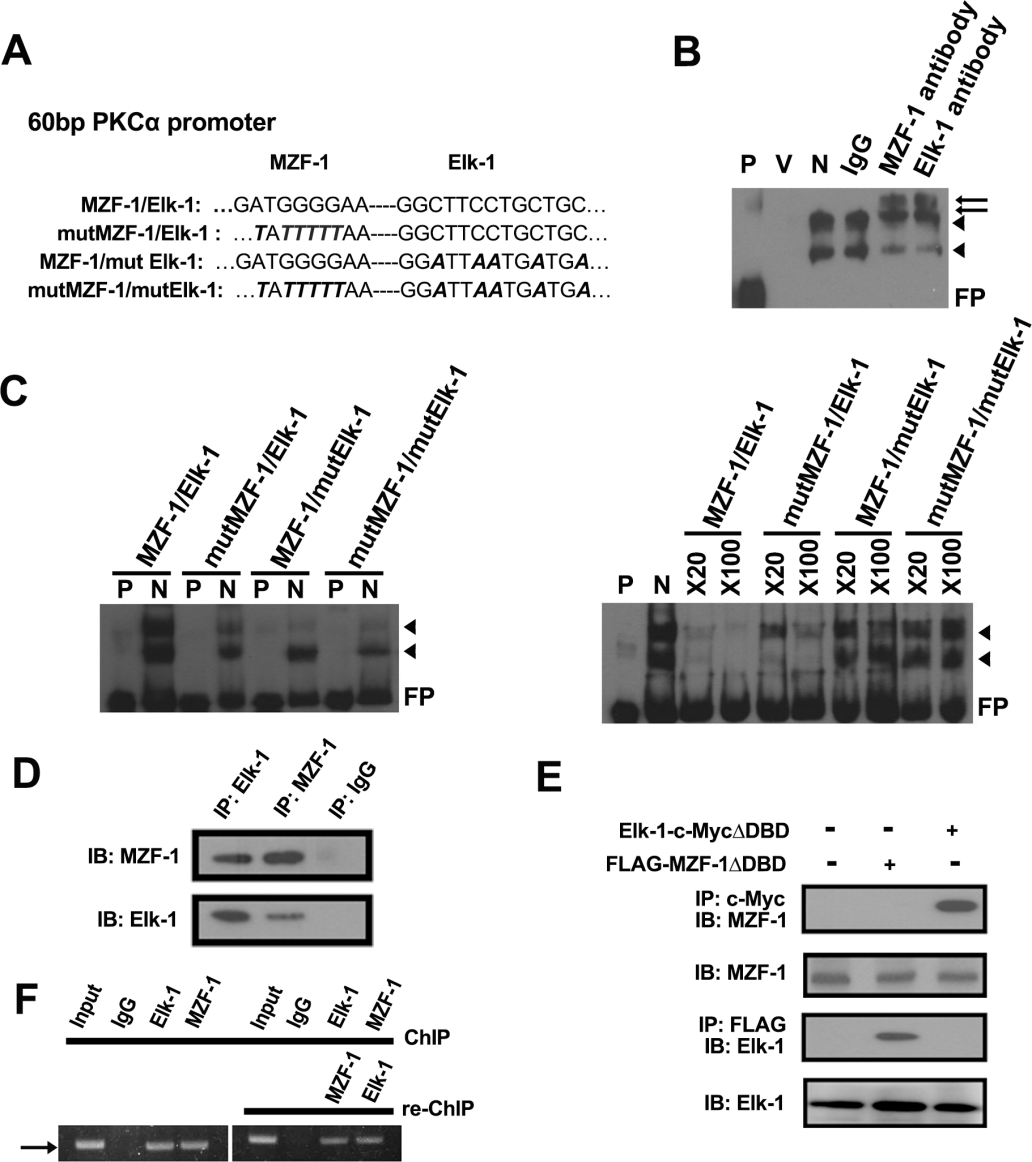
Elk-1/MZF-1 complex binds to the promoter region of PKCα to upregulate its protein expression (**A**) Specific Elk-1/MZF-1 binding sequence at the PKCα promoter in which one of the wild-type (WT) and three of the mutated sequences were designed. Black italics denote mutated sequence. (**B**) Specific binding activities of Elk-1/MZF-1 at the PKCα promoter, as visualized by EMSA. Biotin-labeled WT probes were incubated with MB-231 cell nuclear extracts and IgG/Elk-1/MZF-1 antibodies, and the reaction was resolved on a non-denaturing polyacrylamide gel. DNA–protein complexes are denoted by black arrowheads, and the two supershifted bands are denoted by black arrows. P: probe only; V: nuclear extract only; N: nuclear extract plus probe; FP: free probe. (**C**) Verification of the specific binding activities of Elk-1/MZF-1 by a competitive assay. EMSA of biotin-labeled WT or mutated oligonucleotide probes (left panel) and competition EMSA of biotin-labeled oligonucleotides with unlabeled WT or mutated oligonucleotides in 20-fold to 100-fold molar excess (right panel) are shown. (**D**) Co-IP assay of the interaction between endogenous MZF-1 and Elk-1 in MB-231 cells. Protein extracts were IP with an anti-MZF-1 antibody or anti-Elk-1 antibody/control rabbit IgG, as indicated. The resulting immunoprecipitates were resolved by SDS-PAGE and IB with both antibodies sequentially. (**E**) Interaction between MZF-1 and Elk-1 in MB-231 cells was detected by transfection of 5 μg of either FLAG-MZF-1ΔDBD or Elk-1-c-MycΔDBD to determine the sequence requirements for protein binding. Protein extracts were IP and IB with the indicated antibodies. (**F**) Confirmation of interactions between MZF-1 and Elk-1 and DNA binding activity by ChIP and Re-ChIP assays. In the ChIP assay, chromatin was pulled down with IgG, Elk-1, and MZF-1 antibodies. In the Re-ChIP assay, the pulled-down chromatin was incubated with anti-Elk-1 antibodies, followed by anti-MZF-1 antibodies; the sequence was then reversed. The band denoted by an arrow corresponds to the amplification of the *PKCα* promoter by the PCR performed. The bands amplified from the total chromatin were also employed as control (Input).

To further validate the binding of MZF-1/Elk-1 to the *PKCα* promoter, we mutated the *PKCα* promoter region by replacing all guanine bases with thymines and all cytosines with alanines (Figure 2A). Afterward, we conducted an electrophoretic mobility shift assay (EMSA) and identified two slow migrating bands. Incubation with an antibody against either MZF-1 or Elk-1 resulted in two supershifted bands (Figure 2B). By contrast, binding was reduced when we incubated the nuclear extract with mutant probes with alterations in the Elk-1 and/or MZF-1 binding sites (Figure 2C, left). Moreover, binding decreased more substantially with the addition of 20-fold and 100-fold excesses of unlabeled wild-type probes than with unlabeled mutant probes (mut MZF-1, mut Elk-1, or mut MZF-1/Elk-1) (Figure 2C, right). These combined findings confirm that MZF-1/Elk-1 binds to the *PKCα* promoter and regulates its transcriptional activity.

The Elk-1/MZF-1 DNA-binding sites are proximal on the *PKCα* promoter, and Elk-1/MZF-1 forms a complex to bind to the PKCα promoter [18]; thus, we conducted co-immunoprecipitation (IP) and identified MZF-1 in the complex in MB-231 cells by the Elk-1 antibody and vice versa (Figure 2D). In addition, we transfected cells with truncated Elk-1 (Elk-1-c-Myc-ΔDBD deletion mutant lacking the N-terminal region [19]), and the MZF-1 protein was observed in the complex immunoprecipitated by a c-Myc antibody (Figure 2E). Similarly, when the cells were transfected with the Flag-MZF-1ΔDBD vector (deletion mutant lacking the C-terminal region [20]), the Elk-1 protein was observed in the complex immunoprecipitated by a FLAG antibody. The presence of MZF-1/Elk-1 in all cells in this experiment confirmed that Elk-1 binds to the N-terminal region of MZF-1, whereas MZF-1 binds to the C-terminal region of Elk-1, thereby forming a heterodimeric complex in MB-231 cells.

To determine if the Elk-1/MZF-1 heterodimer forms before binding to the PKC*α* promoter in MB-231 cells, we performed a chromatin immunoprecipitation (ChIP) assay. As shown in Figure 2F the PKC*α* promoter fragment was amplified from the immunoprecipitated complex by using either the Elk-1 or MZF-1 antibody. The results from re-Chip assay indicated that the MZF-1/Elk-1 formed a dimer before binding to the PKC*α* promoter.

### Acidic domain of MZF-1 interacts with heparin-binding domain of Elk-1

**Figure 3:**
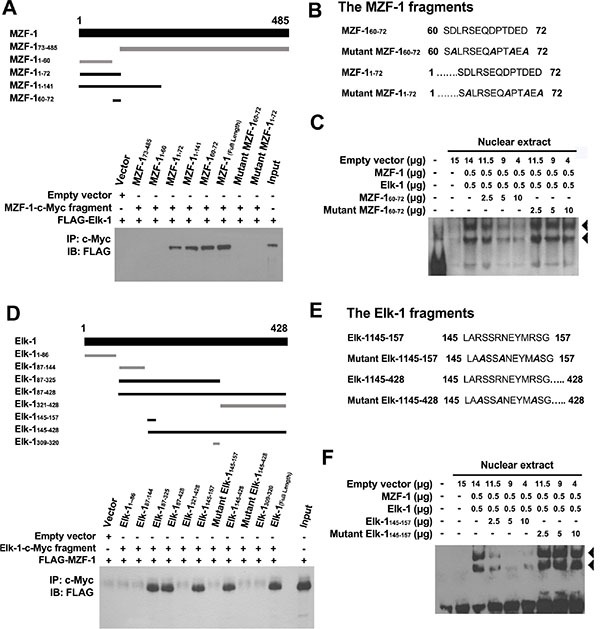
Identification of MZF-1 and Elk-1 interacting domains (**A**) MZF-1-c-Myc and its fragments (amino acids 73–485, 1–60, 1–72, 1–141, and 60–72) were used to indicate the binding domain of MZF-1 (upper panel). Dark-black bands denote the fragments that bound Elk-1 as present in the co-immunoprecipitation assay (lower panel). HEK-293 cells were transfected with 5 μg of FLAG-Elk-1 and either empty vector or the indicated MZF-1-c-Myc fragment. The c-Myc-fused peptides were immunoprecipitated (IP) by the c-Myc monoclonal antibody and then immunoblotted (IB) with the anti-FLAG antibodies. Lysates of HEK-293 cells transfected with the FLAG-Elk-1 only were also immunoblotted as control (Input). “+” indicates the presence of a component, whereas “−“ indicates the absence of a component. (**B**) Two mutant fragments (amino acids 1–72 and 60–72) of MZF-1 in which the negatively charged aspartates in their binding domains were mutated to uncharged alanines (shown in slanted dark-black band). (**C**) Effect of normal and mutant fragments of MZF-1 on specific binding activities of Elk-1 and MZF-1 at the PKCα promoter analyzed by EMSA. The biotin-labeled WT oligonucleotide probes were incubated with a specific concentration of nuclear extracts of the different-treated HEK-293 cells. The HEK-293 cells were transfected with empty vector, Elk-1, MZF-1, MZF-1_60–72_, or mutant MZF-1_60–72_, and the reactions were resolved on a non-denaturing polyacrylamide gel. DNA–protein complexes are denoted by the black arrow-head. FP: free probe. (**D**) Elk-1-c-Myc and its fragments (amino acids 1–428, 1–86, 87–144, 87–325, 87–4281–86, 87–144, 87–325, 87–428, and 60–72) of Elk-1 used to indicate the binding domain (upper panel). Dark-black bands indicate the fragments that bound MZF-1 as present in the co-immunoprecipitation assay (lower panel). HEK-293 cells were co-transfected with 5 μg of FLAG-MZF-1 construct and either empty vector or the indicated Elk-1-c-Myc fragment. Lysates of HEK-293 cells transfected with the FLAG-MZF-1 construct were also immunoblotted as control (Input). (**E**) Two mutant Elk-1 fragments (amino acids 145–157 and 145–428) in which the negatively charged arginines in their binding domains were mutated to uncharged alanines (in slanted dark-black). (**F**) Effects of normal and mutant Elk-1 fragments on specific binding activities of Elk-1 and MZF-1 at the PKCα promoter analyzed by EMSA. Biotin-labeled wild-type oligonucleotide probes were incubated with the indicated concentration of nuclear extracts of the different-treated HEK-293 cells, which were transfected with empty vector, Elk-1, MZF-1, Elk-1_145–157_, or mutant Elk-1_145–157_.

To identify the specific residues through which MZF-1 interacts with Elk-1, we designed various protein fragments for co-IP assays [21] (Figure 3A, top). The full-length MZF-1 and MZF-1_1–72_, MZF-1_1–141_, and MZF-1_60–72_ fragments (all containing the acidic domain) all bound to Elk-1 (Figure 3A, lower panel) but not MZF-1_1–60_ or MZF-1_73–485_ (all lacking the acidic domain). The acidic domain (amino acids 60–72) contains four aspartates and two glutamates upstream of the zinc finger regions [22]; thus, we generated mutations within MZF-1_60–72_ and MZF-1_1–72_, in which the negatively charged aspartates (D61, D67, D70, and D72) were changed to uncharged alanines; their interaction with Elk-1 substantially decreased (Figure 3B). To determine whether inhibition of their interaction will reduce DNA-binding activity, we designed to disrupt the interactions between endogenous Elk-1 and MZF-1 by saturating the protein–protein binding domains with peptides corresponding to the MZF-1_60–72_ fragment. EMSA results demonstrated that MZF-1_60–72_ decreased MZF-1/Elk-1 DNA-binding activity in a dose-dependent manner (Figure 3C). However, the mutant form of the MZF-1_60–72_ fragment did not affect their binding activity. These findings further validated that MZF-1 interacts with Elk-1 through its acidic domain.

The acidic domain is important for interaction with the positively charged heparin-binding domain [23]. Heparin-binding sites often contain clusters (XBX, XBBX, XBBBX; B = basic residue; X = non-basic residue) of basic amino acids (R and K) [24]. Two putative regions in Elk-1 contain such basic clusters: (i) LARSSRNEYMRSG (amino acids 145–157) in the B domain, which contains three clusters (ARS, SRN, and MRS), and (ii) QKGRKPRD (amino acids 311–318) in the D domain, which also contains three clusters (QKG, GRKP, and PRD).

Thus, we constructed several Elk-1 fragments to determine the region through which Elk-1 interacts with MZF-1 (Figure 3D, top). The fragments that contained amino acids 145–157 interacted with MZF-1 (Figure 3D, bottom), whereas mutation of the positively charged arginines to alanines (R147, R150, and R155) within this region abolished their interaction (Figure 3E). The addition of the Elk-1_145–157_ fragment also decreased MZF-1/Elk-1 DNA-binding activity in a dose-dependent manner (Figure 3F), whereas the mutant form did not. These findings identified the region spanning amino acids 145–157 as the heparin-binding domain, which binds to MZF-1.

### Interruption of MZF-1 and Elk-1 heterodimer reduces the malignant phenotypes of TNBC cells

**Figure 4:**
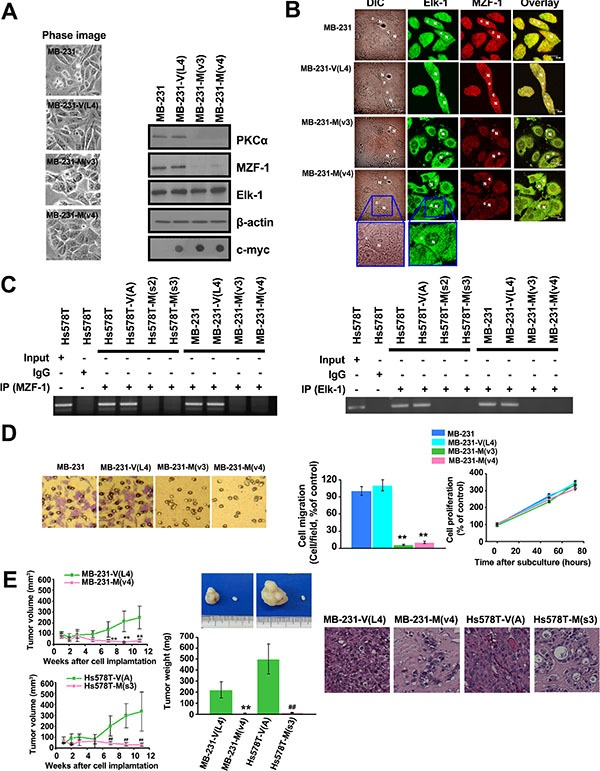
Disrupting the interaction between Elk-1 and MZF-1 decreases PKCα expression, drug resistance and malignant phenotypes (**A**) Changes in morphology and gene expression in MZF-1_60– 72_ construct-transfected stably cloned MB-231 cells. β-actin was used as a control, and c-Myc was used as a marker for transfected cells. (**B**) Confocal microscopy showing the distribution of the Elk-1 and MZF-1 proteins. Cells were stained with antibodies against Elk-1 and MZF-1, followed by the appropriate FITC- or rhodamine-conjugated secondary antibodies. Confocal slices of 0.5 and 0.6 μm thicknesses were obtained, and images were obtained focusing the center of the nucleus. “N” denotes the nucleus; “C” denotes the cytosol. (**C**) ChIP assays indicating the binding activity of endogenous Elk-1 and MZF-1 to the PKCα promoter in various cells. The assays were performed using an MZF-1 (left panel) or Elk-1 (right panel) antibody. (**D**) Visualization and quantification of cell migration and proliferation of modified MB-231 cells. (**E**) Tumor growth in nude mice after xenografts of modified MB-231 and Hs578T cells (left panels). Tumors removed from the mice were weighed (middle images and graph) and sliced before histological examination (right images). **p < 0.01 compared with the empty vector-transfected MB-231 group, ^##^*p* < 0.01, compared with the empty vector-transfected Hs578T group.

The effect of MZF-1_60–72_ on PKCα functions was investigated because MZF-1_60–72_ has been shown to disrupt the endogenous MZF-1/Elk-1 DNA-binding activity. The results showed that stable clone TNBC MB-231 and Hs578T breast cancer cells expressing MZF-1_60–72_ [MB-231-M(v3), MB-231-M(v4), Hs578T-M(s2), and Hs578T-M(s3)] were more rounded in shape than the elongated parental and vector control cells (Figure 4A and Supplementary Figure S1A). In addition, PKCα and MZF-1 expression levels decreased in the stable clone cells, whereas the Elk-1 levels remained relatively the same. Fluorescence imaging analysis of confocal immunofluorescence microscopy revealed that both Elk-1 and MZF-1 were predominantly present in the cytosol but more equally distributed in the nucleus and cytosol of the parental and empty vector cells (Figure 4B and Supplementary Figure S1B).

To determine whether MZF-1_60–72_ blocks endogenous Elk-1 and MZF-1 from binding to the PKCα promoter, we conducted ChIP assays. The PKCα promoter fragments amplified from the immunoprecipitated complex by using either Elk-1 or MZF-1 antibodies decreased in all MZF-1_60–72_-expressing stable cells (Figure 4C). This decrease demonstrates that the fragment not only interrupts endogenous Elk-1/MZF-1 from binding but also interrupts them from interacting with the promoter.

We then examined the effects of MZF-1_60–72_ on the tumorigenic potential of MB-231 and Hs578T breast cancer cells. Cell migration significantly reduced by 80%–90% in MZF-1_60–72_-expressing stable cells relative to parental and control cells (Figure 4D and Supplementary Figure S1C). However, no changes were observed in cell proliferation. The efficacy of MZF-1_60–72_ was then evaluated in a breast cancer xenograft mouse model. Mice injected with MZF-1_60–72_-expressing stable cells developed tumors more slowly than those injected with vector control cells (Figure 4E, left). The maximum inhibition of tumor growth was 91.0% ± 5.2% (*n* = 5) for Hs578T and 90.7% ± 4.6% (*n* = 5) for MB-231 stable cells. The mean tumor growth inhibition from 7 weeks to 11 weeks was 85.6% ± 4.1% for Hs578T and 84.4% ± 6.7% for MB-231 stable cells. Tumor weight from mice injected with MZF-1_60–72_-expressing stable cells also significantly decreased (Figure 4E, middle), and the cells from these tumors exhibited more interstitial tissues when tubular structures first appeared (Figure 4E, right). Moreover, leukocyte infiltration near the outer edge of the tumor occurred, which may indicate limited tumor growth [25]. The inhibitory effects of MZF-1_60–72_ on tumorigenesis further demonstrated that this region of MZF-1 is essential for the formation of the MZF-1/Elk-1 interaction and transcriptional activation of PKCα which is highly expressed in cancer cells.

### Blocking MZF-1/Elk-1 heterodimer formation decreases EMT potential

**Figure 5:**
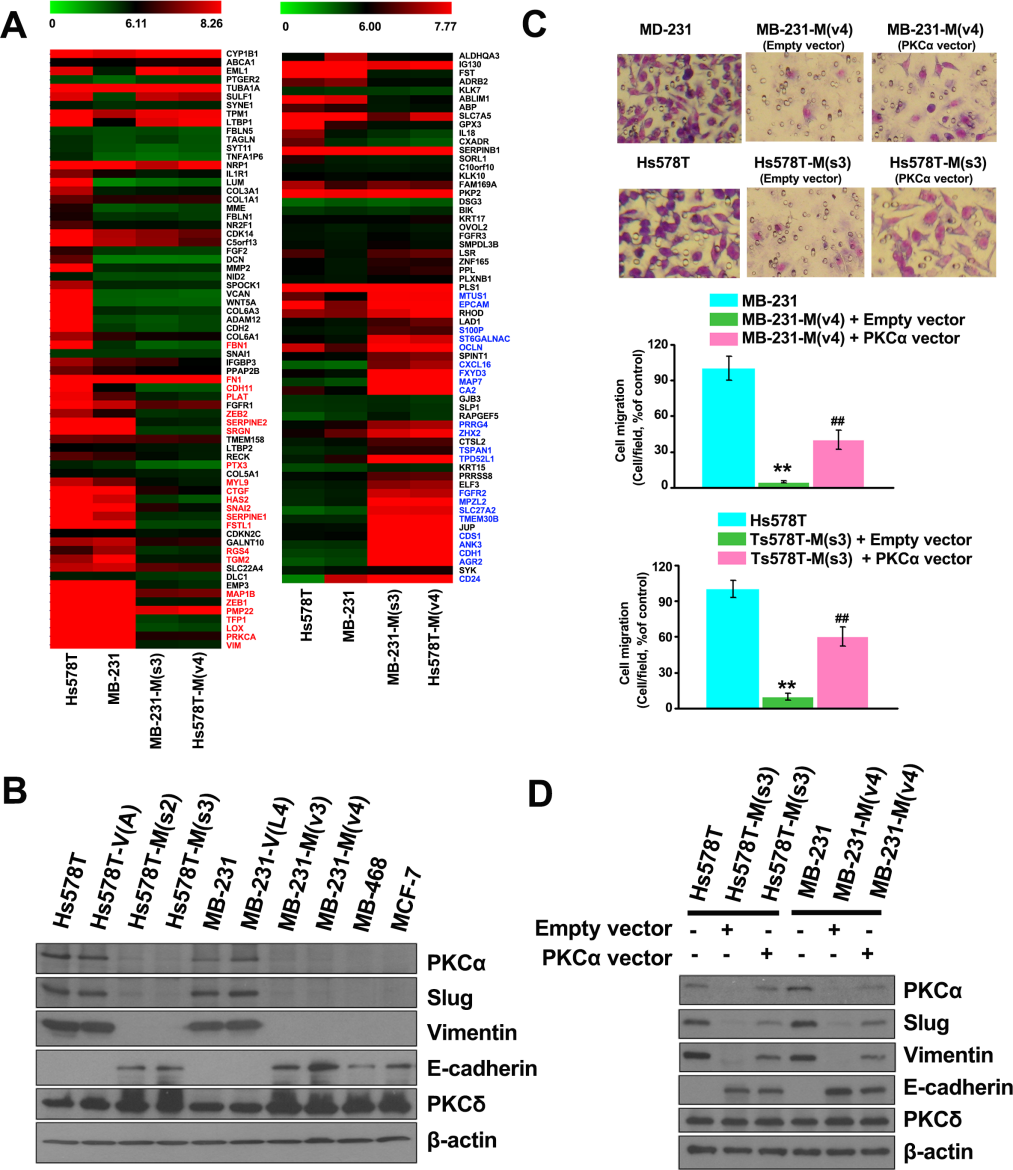
Disrupting the interaction between MZF-1 and Elk-1 decreases EMT potential (**A**) Comparison of the gene expression profiles of upregulated (left panel) and downregulated (right panel) EMT-related genes in MZF-1_60–72_-transfected Hs578T and MB-231 cloned cells, as determined by microarray with those of the parental cells. (**B**) Immunoblotting analysis of changes in protein levels in the parental and transfected cells and in the less malignant MB-468 (MB-468) and MCF-7 cells. (**C**) Visualization and quantification of cell migration of PKCα-transfected cells by migration assay. MZF-1_60–72_ construct-transfected stably cloned cells were transfected with the empty-vector or full-length PKCα construct for 3 days, and migration assay was performed. **p < 0.01 compared with the parental cells, ^##^*p* < 0.01 compared with the empty vector-transfected groups. (**D**) Immunoblotting analysis of changes in protein levels in the PKCα-co-transfected cells. β-actin was used as a control.

PKCα has been shown to play a role in EMT [9, 26–28]; thus, we performed gene expression profiling in MZF-1_60–72_-expressing stable breast cancer cells, particularly focusing on “EMT-core genes” [29, 30]. Out of the 22,203 genes analyzed in both cell lines, 821 and 931 genes were upregulated or downregulated, respectively. Diverse biological functions were found among the affected genes, including 24 EMT-core-upregulated genes (*CDH11*, *CTGF*, *EMP3*, *FBN1*, *FN1*, *FSTL1*, *HAS2*, *LOX*, *MAP1B*, *MYL9*, *PLAT*, *PMP22*, *PRKCA*, *PTX3*, *RGS4*, *SERPINE1*, *SERPINE2*, *SNAI2*, *SRGN*, *TFPI*, *TGM2*, *VIM*, *ZEB1*, and *ZEB2*) that were decreased (Figure 5A, left, red) and 24 EMT-core-downregulated genes (*AGR2*, *ANK3*, *CA2*, *CD24*, *CDH1*, *CDS1*, *CXCL16*, *ELF3*, *EPCAM*, *FGFR2*, *FXYD3*, *JUP*, *MAP7*, *MPZL2*, *MTUS1*, *OCLN*, *PRRG4*, *S100P*, *SLC27A2*, *ST6GALNAC2*, *TMEM30B*, *TPD52L1*, *TSPAN1*, and *ZHX2*) that were increased (Figure 5A, right, green). Similar changes in EMT-core-upregulated and -downregulated genes were also observed when the malignant human breast cancer cells MB-231 and Hs578T were compared with the less malignant human breast cancer cells MB-468 and MCF-7 (Supplementary Figure S2). The protein levels of PKCα, Slug (*SNAI2)*, and Vimentin (*VIM*) markedly decreased, whereas E-cadherin (*CDH1*) substantially increased; PKCδ, which belongs to the PKC family, remained unchanged (Figure 5B). Changes in the protein expression of these EMT-core genes were also similar in Hs578T cells stably expressing Elk-1_145–157_ (Supplementary Figure S3).

To validate the function of PKCα in EMT, both MB-231-M(V4) and Hs578T-M(s3) cells were transfected with full-length PKCα. The expression of PKCα significantly increased cell migration in MB-231-M(V4) and Hs578T-M(s3) cells from 5% to 41% and 9% to 62%, respectively, compared with the untransfected cells (Figure 5C). The expression levels of the EMT-related genes (Slug, Vimentin, and E-cadherin) were also reversed (Figure 5D). These findings indicate that the MZF-1/Elk-1 heterodimer affects EMT-related genes through the PKCα pathway.

### TAT-fused peptides inhibit MZF-1/Elk-1 heterodimer formation

**Figure 6:**
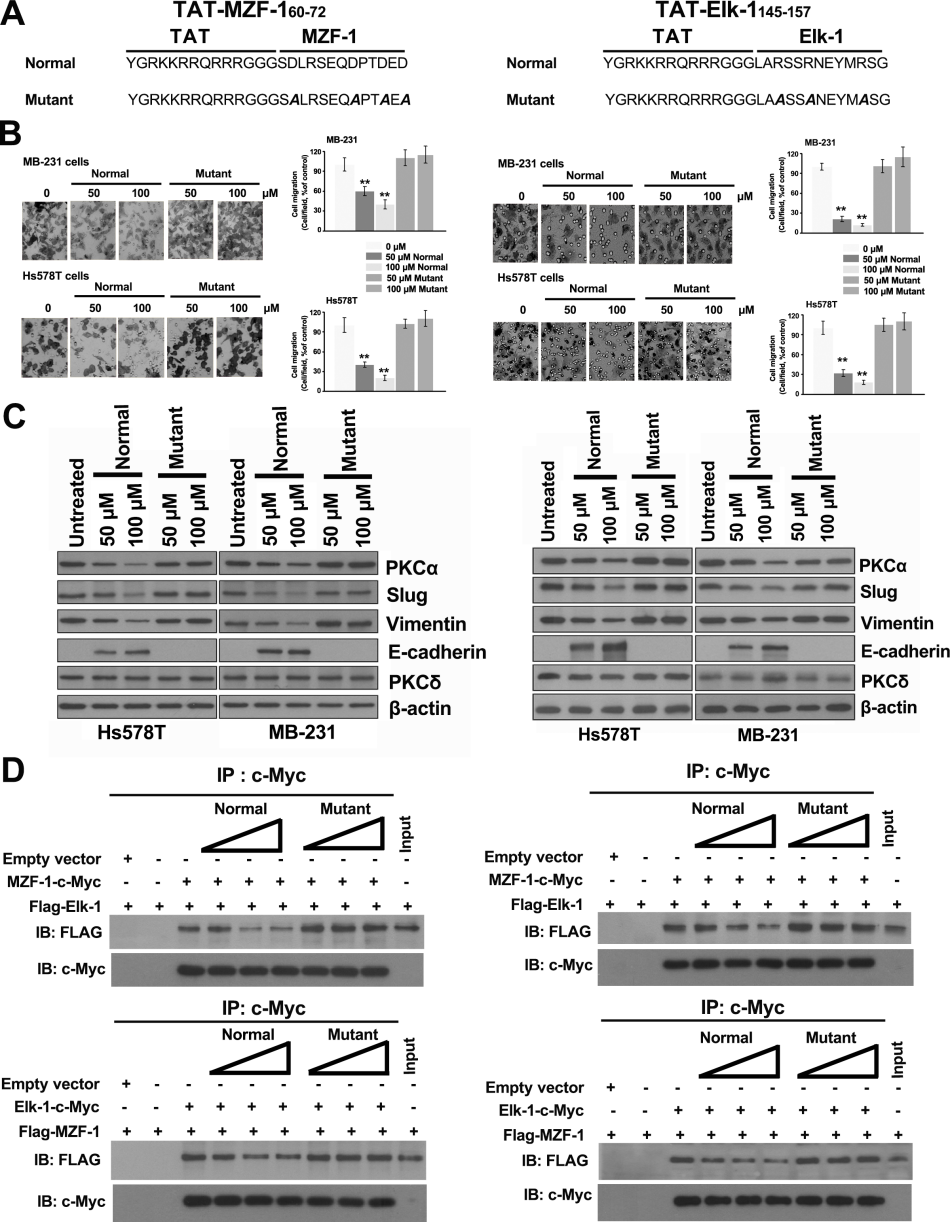
TAT-fused peptides decrease PKCα expression and reduce EMT (**A**) Sequences of the TAT-fused TAT-MZF-1_60–72_ and TAT-Elk-1_145–157_ peptides (normal and mutant). (**B**) Effects of TAT-fused peptides on cell migration. Cell migration assay was performed 3 days after peptides were added to the cells. (**C**) Immunoblotting analysis of changes in protein levels in the TAT-fused peptide-treated cells 3 days post-treatment, with β-actin as the control. (**D**) Detection of the effects of TAT-fused peptides on Elk-1 and MZF-1 interaction by co-immunoprecipitation assay. HEK-293 cells transfected with empty vector, FLAG-Elk-1, MZF-1-c-Myc, FLAG-MZF-1, or Elk-1-cMyc construct. “+” indicates the presence of each item, and “−” indicates the absence of each item. Different concentrations (50, 100, and 150 nmol) of TAT-fused peptides (normal or mutant) were then added to the lysates and incubated overnight at 4°C, and then subjected to IP with the anti-c-Myc antibody, followed by IB against FLAG and c-Myc. Lysates of HEK-293 cells transfected with FLAG-Elk-1 or FLAG-MZF-1 vector were indicated as control “Input”.

Given that the interruption of the MZF-1/Elk-1 interaction significantly affected EMT, we examined the efficacy of MZF-1_60–72_ and Elk-1_145–157_ peptides against breast cancer cells by using cell-permeable peptides fused to a short sequence (residues 48–57) of the HIV *trans*-activating regulatory protein (TAT) [31]. Hs578T and MB-231 cells treated with either TAT-MZF-1_60–72_ (Figure 6A, left) or TAT-Elk-1_145–157_ peptide (Figure 6A, right) reduced cell migration (Figure 6B), and the levels of EMT-related (PKCα, Slug, and Vimentin) and MET-related (E-cadherin) proteins noticeably increased and decreased, respectively (Figure 6C). However, no changes were observed in the cells treated with a TAT-fused mutant peptide. These findings are consistent with above results (Figure 5B). Moreover, co-IP assay results indicated that both MZF-1_60–72_ and Elk-1_145–157_, but not mutant TAT-fused peptides, reduced the binding of MZF-1 to Elk-1 and vice versa in a dose-dependent manner (Figure 6D). The combined results suggested that the interaction between MZF-1 and Elk-1 may be considered as a therapeutic target.

## DISCUSSION

PKCα is often involved in studies on the mechanism of EMT because it has been a key candidate signaling molecule in the progression of many carcinomas [9, 12, 26–28]. EMT increases the adaptation potential of cancer cells, such as bypassing cellular senescence, as well as the subsequent development of CSCs [32]. CSCs are responsible for driving tumor growth, recurrence, and metastasis [33, 34] and demonstrates highly aggressive cancer traits. The present study revealed that both PKCα and EMT can be regulated by the MZF-1/Elk-1 heterodimer in TNBC cells. PKCα can also be reduced by treating cells with fragments containing either of the two binding domains, namely, MZF-1_60–72_ or Elk-1_145–157_. The effects of this fragment saturation can be reversed by transfection with full-length PKCα, suggesting that the formation of MZF-1/Elk-1 heterodimers occurs in the upstream of the induction of EMT mediated through the PKCα pathway in TNBC cells. In addition, although MZF-1 has been reported to use various domains such as SCAN or zinc finger motifs to bind to its co-factors [35, 36], there have been no known reports of MZF-1 using its acidic domain for protein-protein interaction in the promotion of TNBC malignancy.

Tan et al. [13] reported that ErbB2-overexpressing breast tumors showed significantly higher positive rates of PKCα activation. The activated PKCα then increases the expression of urokinase-type plasminogen activator receptor (uPAR) and cancer cell invasion. In our microarray data, we found that, by interrupting MZF-1/Elk-1 heterodimers in TNBC cells, PKCα decrease and uPAR decrease are associated despite no changes in ErbB2. Other microarray data also indicated that the expression of ErbB2 is lower in TNBC cell lines than in non-TNBC cell lines despite both PKCα and uPAR being higher in TNBC cell lines [37], thus suggesting that MZF-1/Elk-1 heterodimers may be upstream for PKCα in PKCα-overexpressing TNBC cells, a parallel position to ErbB2 in ErbB2-overexpressing breast tumors.

In this study, the expression levels of MZF-1 and EMT-related genes decreased in cells treated with the MZF-1/Elk-1 binding domain. This effect was reversed by co-treatment with the protease inhibitor to inhibit MZF-1 degradation (Supplementary Figure S1D), indicating that the heterodimer may be a means to slow MZF-1 degradation and prolong its regulatory functions on EMT. This phenomenon may explain MZF-1 over-expression in TNBC cells [16] without changes in the mRNA levels of MZF-1 [37]. In addition, the inhibition of MZF-1 by shRNA transfection can inhibit the expression of PKCα and the EMT potential in TNBC cells (Supplementary Figure S1E), suggesting the important role of MZF-1 in TNBC cells.

MZF-1 has also been implicated to regulate various factors in many cancers and cellular malignancies [38]. MZF-1 can cause MYC transcription activity, as well as induce migratory, invasive, and *in vivo* metastatic potential in solid tumor cells [39]. Similarly, MZF-1 can induce production of TGF-β1 in breast cancer and plays a critical role in osteopontin-induced mesenchymal-stem-cell to cancer-associated-fibroblast transformation [40]. However, in TNBC cells treated with MZF-1/Elk-1 binding domain, MZF-1 decreased without changes in MYC and TGF-β1 being observed in our microarray data. In addition, a decrease in the expression of AXL, a novel receptor tyrosine kinase known to be an essential EMT-induced regulator of breast cancer and regulated by MZF-1 [41–44], was also observed, and the decrease can be reversed by rescuing MZF-1 expression with protease inhibitor (Supplementary Figure S1D). Other microarray data support that the expression of MYC and TGF-β1 in TNBC cell lines is lower than that in non-TNBC cell lines regardless of AXL being higher in TNBC cell lines [37]. Thus, this divergence of expression levels can be attributed to the difference in cell conditions between TNBC and non-TNBC.

An increase in Elk-1 phosphorylation can cause increased nuclear translocation, which leads to cancer progression [45]. Interestingly, our data showed a halt in nuclear translocation in MZF-1_60–72_-treated cells (Supplementary Figure S1F) without change in Elk-1 phosphorylation. This occurrence may be attributed to the loss of its MZF-1 partner, which degraded quickly in the fragment-treated cells, disabling Elk-1 translocation. This indicates that Elk-1 nuclear localization is directly linked to the presence of MZF-1, and the interaction of MZF-1 with Elk-1 may be involved in the nuclear translocation of Elk-1 in TNBC cells.

In the current study, we used Elk-1_145–157_ and MZF-1_60–72_ fragment-fused peptides as a novel inhibitor to target the protein–protein interface and reduce PKCα transcriptional activities and its subsequent downstream expressions. The Mochly-Rosen laboratory [11] implies that a phosphorylation inhibitor can specifically target PKCα substrates, but the cells affected are nonspecific. By contrast, our laboratory targeted the PKCα transcription factors Elk-1 and MZF-1 in PKCα-, Elk-1-, and MZF-1-expressing TNBC cells only without any adverse effects on cells that do not express them. Therefore, considering the high correlation between the two transcription factors MZF-1/Elk-1 and PKCα expression in TNBC patients, inhibiting the MZF-1/Elk-1 interaction represents a novel and feasible strategy to specifically inhibit PKCα expression and, consequently, TNBC.

## MATERIALS AND METHODS

### Immunohistochemical (IHC) staining

The array slides (breast cancer BR20834 and TNBC BR1503b) were purchased from US Biomax Inc. (Rockville, MD, USA). The BR20834 slides included 205 invasive ductal carcinomas, 2 invasive lobular carcinomas, and 1 invasive papillary carcinoma. However, 15 invasive ductal carcinomas were lost during the evaluation. The BR1503b slides included 7 breast intraductal carcinoma and 60 breast invasive ductal carcinoma, which contained 30 TNBC, duplicate cores per case. Detailed information for this array can be viewed at http://www.biomax.us/tissue-arrays/. The sections were immunostained for PKCα (1:200) (BD Biosciences, San Jose, CA, USA), Elk-1 (1:400) (Santa Cruz, CA), and MZF-1 (1:400) (Santa Cruz) and the expression was scored by staining as follows: 1+, weak; 2+, moderate; and 3+, strong.

### Cell lines

Cancer cells from various human organs, namely, breast cancer Hs578T (BCRC no.60120), MDA-MB-231 (MB-231) (BCRC no.60425), and MCF-7 (BCRC no.60436) cells from the breast; HEK-293 (BCRC no.60019) cells from embryonic kidney, were purchased from the Bioresources Collection and Research Center, Food Industry Research and Development Institute (Hsinchu, Taiwan). MDA-MB-468 (MB-468) (ATCC no. HTB-132) cells from breast were obtained directly from the ATCC (Manassas, VA, USA). The cells were cultured in media specific to each cell line, and supplemented with 10% fetal bovine serum, 100 units/ml penicillin G, and 100 μg/ml streptomycin (Gibico, grand Island, NY, USA) in a humidified atmosphere containing 5% CO_2_ at 37°C.

### Plasmid construction

The pcDNA-Elk-1 and pcDNA-MZF-1 plasmids were constructed using the basic cytomegalovirus (CMV) promoter-containing pcDNA3 vector (Invitrogen, Carlsbad, CA, USA). Open reading frames of the human MZF-1 (GenBank Accession No. AF161886 10781-12235 bp) and Elk-1 (GenBank Accession No. AB016193 101-1384 bp) genes were amplified from MB-231 cells by reverse transcription-polymerase chain reaction (RT-PCR) and cloned into vectors; the resulting recombinant plasmids were designated as pcDNA-MZF-1 and pcDNA-Elk-1, respectively. Supplemental Table S1 lists the primer sequences and the restriction sites used for cloning. The PCR products were isolated and cloned into the pcDNA^™^ 3.1/myc-His vector (Invitrogen).

The plasmids containing different fragments of MZF-1-c-Myc (encoding amino acids 1-60, 1-141, 73-485, 60-72 and its mutant, and 1-72 and its mutant) and Elk-1-c-Myc (encoding amino acids 1-86, 87-144, 87-325, 87-428, 309-320, 321-428, 145-428, 145-157 and its mutant, and 145-428 and its mutant) were amplified from pcDNA-MZF-1-c-Myc and pcDNA-Elk-1 by PCR (see Supplemental Table S1 primer sequences and restriction sites), respectively. The PCR products were isolated and cloned into the pcDNA^™^ 3.1/myc-His vector (Invitrogen).

The plasmids containing FLAG-MZF-1 (encoding full length, amino acid 1-485 and amino acids 1-141) and FLAG-Elk-1 (full length amino acids 1-428) were amplified from pcDNA-MZF-1-c-Myc and pcDNA-Elk-1-c-Myc by PCR (see Supplementary Table S1 primer sequences and restriction sites), respectively. The PCR products were isolated and cloned into the pFLAG-CMVTM-2 vector (Sigma-Aldrich).

The plasmids containing full-length PKCα-c-Myc (encoding amino acids 1-672) was constructed by cloning PKCα (45-2060 bp) in the pcDNATM 3.1/myc-His vector. The open reading frame of the human PKCα (GenBank Accession No. NM_002737) gene was amplified from Hs578T cells by RT-PCR. Supplementary Table S1 lists the primer pairs and their corresponding restriction enzymes. The PCR products were isolated and cloned into the pcDNATM 3.1/myc-His vector (Invitrogen) after digesting with the appropriate restriction enzymes.

All of the sequences were aligned and identified in Supplementary Figure S4–S35.

### Transfection, stable clone establishment and treatment with TAT-fused peptides

Lipofectin was used for transfection. Cells were cultured in 60 mm dishes containing minimum essential Dulbecco's Modified Eagle medium (DMEM) supplemented with 10% fetal calf serum (FCS) at 37°C .

Stable clones were established by seeding low-passage cells at a density of 3 × 10^5^ cells in 60-mm tissue culture dishes and transfecting the cells with 5 μg MZF-1_60–72_ plasmid using Lipofectamine 2000. Stable clones were selected by growing the cells in DMEM supplemented with geneticin (G418; 600 μg/ml) at 37°C for five weeks. Individual clones were then transferred to 96-well plates and grown until confluence. After being transferred to flasks, the cells were cultured until confluence, harvested, and frozen in liquid nitrogen for further experiments.

The TAT-fused peptides were designed such that the TAT moiety corresponded to amino acid residues 48-57 of the HIV TAT protein [31], the MZF-1_60–72_ moiety corresponded to residue 60–72 of the human MZF-1 protein, and the Elk-1_145–157_ moiety corresponded to residues 145-157 of the human Elk-1 protein. The TAT and MZF-1/Elk-1 moieties were linked by three glycine linker residues. The TAT-fused peptides were synthesized by MDBio, Inc. (Taipei, Taiwan). For transduction of the TAT fusion proteins, cells were cultured to 50–60% confluence. The culture medium was removed and replaced with fresh, serum-free medium, followed by the addition of the TAT fusion proteins at the indicated concentrations. Three days post-treatment, the cells were used for migration assays and western blotting.

### EMSA analysis

EMSA analysis was performed using a LightShift™ chemiluminescent EMSA kit (Pierce, Rockford, IL, USA) with 15 μg of nuclear extract as previously described^13^. Biotin-labeled double-stranded wild-type MZF-1/Elk-1 oligonucleotides containing the MZF-1 and Elk-1 binding sites in the human PKCα promoter; mutant MZF-1/Elk-1 oligonucleotides were used as probes.

### Co- IP and immunoblotting (IB) analysis

Cells were lysed in a modified radioimmunoprecipitation assay buffer. The cell lysates were then centrifuged at 16000 × g for 10 min and kept on ice. Approximately 500 μL lysate was incubated with 2 μg of specific antibodies. After incubating for 18 h, the specific proteins were purified using according to the manufacturer's protocol. The c-Myc-fusion proteins were purified using c-Myc monoclonal antibody-agarose beads (Pierce Mammalian c-Myc Tag IP/Co-IP kit, Pierce, Rockford, IL, USA).

The protein levels were determined by immunoblotting assays. The membranes were probed with the following specific antibodies: anti-PKCα, δ, ε, or ι (BD Biosciences); anti-c-Myc (Immobilon-P; Millipore, Bedford, MA, USA); anti-urokinase-type plasminogen activator (uPA) (GeneTex, Inc., Irvine, CA, USA); anti-FLAG, anti-E-cadherin, anti-vimentin and anti-Slug (Cell Signaling, Beverly, MA, USA); anti-Elk-1, anti-phospho-Elk-1, anti-MZF-1, and anti-β-actin (Santa Cruz) in blocking buffer at 4°C overnight. After the blots were incubated with horseradish peroxidase-labeled anti-mouse or anti-rabbit secondary antibodies (Promega), antibody-reactive proteins were detected using a chemiluminescent substrate (GE Healthcare).

### ChIP assay

ChIP was performed as previously described Reid et al. [46]. The samples were pre-cleared with protein A agarose (Sigma-Aldrich) for 30 min at 4°C and incubated with IgG, MZF-1, or Elk-1 antibodies (Santa Cruz) overnight at 4°C. The region between −760 and −550 of the PKCα promoter was amplified from the immunoprecipitated chromatin using the primers: sense, 5′-GGTACAGGCAGCTAAAACAC-3′, and antisense, 5′-GTCTTCCTTCTCCCACTCC-3′. After PCR, the 210 bp product was resolved and visualized on a 2% agarose gel.

For re-ChIP, a previously described methodology [46], was followed. The precipitated complexes eluted from the primary immunoprecipitates were pooled from three or four reactions and incubated with 30 μl ChIP elution buffer (50 mM NaHCO_3_, 1% SDS). The samples were mixed for 30 min at room temperature and centrifuged, and the supernatants were collected. Further supernatant Re-ChIP assays and result analyses were performed as previously described for primary ChIP immunoprecipitation.

### Cell proliferation, migration, and invasion assays

Cell proliferation, migration and invasion were analyzed as previously described [14].

### Animal studies

Female 4- to 6-week-old BALB/c nude mice were purchased from the National Laboratory Animal Center (Taipei, Taiwan) and were housed in conventional cages with free access to water and rodent chow at 20–22°C with a 12-hour light–dark cycle. All procedures involving laboratory animal use were in accordance with the guidelines of the Instituted Animal Care and Use Committee of China Medical University (IACUC, CMU) for the care and use of laboratory animals and all experimental procedures were approved by IACUC-CMU. The cancer cells were detached from culture dishes by trypsinization 48 h later and then washed three times in serum-free DMEM. Approximately 1 × 10^7^ cells in 100 μl volume were subcutaneously injected into the right posterior flank of the mice using a 1 ml syringe with a 24-gauge needle. Five mice were used in each group, and the experiment was repeated twice. The tumor volume was calculated using the formula 0.5236 × L1 (L2)^2^, where L1 is the long diameter and L2 is the short diameter. The inhibition of tumor growth was calculated using the following formula: (tumor volume in control group - tumor volume in test group)/(tumor volume in control group) × 100%. After 2 or 3 months, the mice were sacrificed to remove the tumors, and the tumor mass was measured and subjected to histopathological examination.

### Confocal immunofluorescence microscopy

Cells were seeded at a density of 2 × 10^5^ cells/well in 6-well plates and incubated in complete medium overnight at 37°C. The cells were then fixed in 4% paraformaldehyde for 30 min and permeabilized with 0.1% saponin, 2% goat serum (Vector Laboratories) and 0.02% NaN3 for 15 min. Elk-1 and MZF-1 were visualized using their particular antibodies (1:200) followed by FITC-conjugated goat anti-mouse IgG antibody and rhodamine-conjugated goat anti-rabbit IgG antibody (Rockland Immunochemicals, Inc., Gilbertsville, PA.), respectively. Immunofluorescence densities and images were obtained using a Leica TCS SP2 confocal microscope detection system (Wetzlar, Germany) at excitation wavelengths of 488 and 543 nm. Emissions were detected using bandpass filters of 505 nm to 525 nm and 578 nm to 623 nm.

### Microarray analysis

Total cellular RNA was isolated using an RNeasy mini kit. (QIAGEN GmbH, Hilden, Germany). RNA quantity and purity were assessed at 260 and 280 nm, respectively, using a spectrophotometer (ND-1000 Nanodrop,Labtech International Ltd, East Sussex, UK). The RNA (300 ng) was amplified and labeled using the GeneChip WT sense target labeling and control reagents (Affymetrix 900652; Affymetrix Inc., Santa Clara, CA) for expression analysis. An Affymetrix GeneChip Human Gene 1.0 ST array was hybridized for 17 h at 45°C at 60 rpm. The arrays were washed (Affymetrix Fluidics Station 450), stained using streptavidin-phycoerythrin (GeneChip^®^ hybridization, wash, and stain Kit, Affymetrix 900720) and subsequently scanned using an Affymetrix GeneChip^®^ Scanner 3000. The resulting data were analyzed using Affymetrix Expression Console software with default RMA parameters. Among the differentially expressed genes, genes designated as upregulated were over-expressed by at least twofold compared with the untreated control (*p* < 0.05). Genes designated as downregulated were under-expressed by at least 0.75-fold compared with the untreated control (*p* < 0.05). The data present in this study have been deposited in the National Center for Biotechnology Information (NCBI) Gene Expression Omnibus and can be accessed through Gene Expression Omnibus Series accession number GSE56306 (http://www.ncbi.nlm.nih.gov/geo).

### Statistical analysis

The data are expressed as the mean ± S.D. and were analyzed by ANOVA. Pearson's chi-squared test and Student's *t*-test were used in two-group comparisons. *P* < 0.05 was considered as statistically significant.

## SUPPLEMENTARY MATERIALS FIGURES AND TABLE


