# Supplementary material for: MZF-1/Elk-1 interaction domain as therapeutic target for protein kinase Cα-based triple-negative breast cancer cells
Source: Oncotarget. 2016 Aug 17;7(37):59845–59. doi: 10.18632/oncotarget.11337 (PMC5312353; doi:10.18632/oncotarget.11337)
Supplement: Supplementary file 1 [file oncotarget-07-59845-s001.pdf]

MZF-1/Elk-1 interaction domain as therapeutic target for protein kinase Cα-based triple-negative breast cancer cells

Supplementary Materials

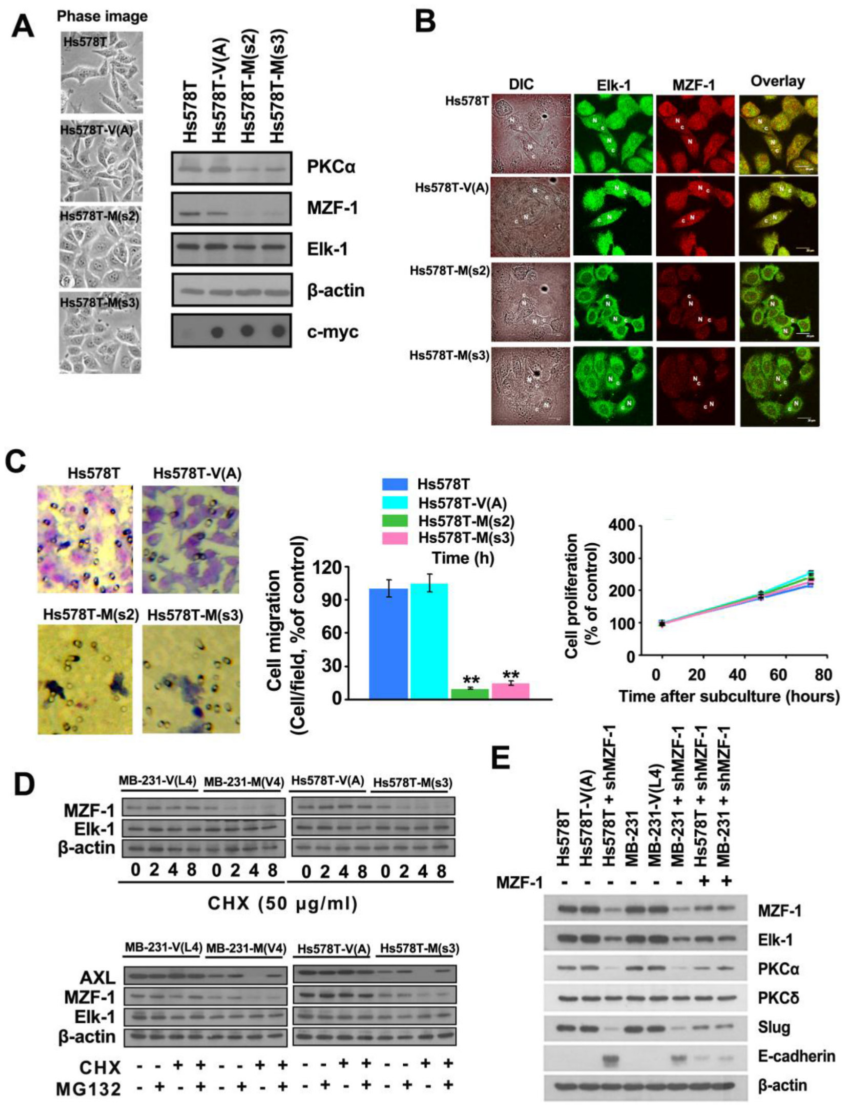

Supplementary Figure S1: Disrupting Elk-1 and MZF-1 binding affects PKCα expression and drug sensitivity in MZF-1$_{60-72}$ construct-transfected stably cloned Hs578T cells. (**A**) Changes in morphology and gene expression of MZF-1$_{60-72}$-transfected stable-cloned Hs578T cells. β-Actin was used as an internal control, and c-Myc was used as a marker of transfected cells. (**B**) Confocal microscopy showing the distribution of the Elk-1 and MZF-1 proteins. Cells were stained with antibodies against Elk-1 and MZF-1, followed by FITC- and rhodamine-conjugated secondary antibodies, respectively. Confocal slices of between 0.5 and 0.6 μm were obtained, and the images were taken through the center of the nucleus. "*N*" indicates the nucleus; "c" indcates the cytosol. (**C**) Visualization and quantification of cell migration and proliferation of modified Hs578T cells, as determined by the migration and proliferation assay. The data indicate the mean ± S.D. ($n = 3$ in each group). $*p < 0.05$, $**p < 0.01$ compared with the control Hs578T group. (**D**) The degradation of MZF-1 dependent on proteasome activity. The rate of MZF-1 degradation of in tested stable-cloned Hs578T and MB-231 cells were decreased as compared to their parent cells (up panel), which were determined by using cycloheximide treatment in near confluent cells. AXL and MZF-1 cycloheximide chase analysis in the presence or absence of the proteasome inhibitor MG132 in immunoblotting analysis showed that the degraded protein is rescued by the proteasome inhibitor (down panel). (**E**) The effect of MZF-1 on the EMT in the parental Hs578T and MDA-MB-231 cells was tested by MZF1 shRNA knockdown assay and detected by immunoblotting analysis. The data indicated that the inhibition of MZF-1 can inhibit the expression of PKCα and the EMT potential in TNBC cells, and forced MZF-1 expression reversed the changes. "+" indicates co-treated with MZF-1 plasmid; "−" indicates without co-treatment.

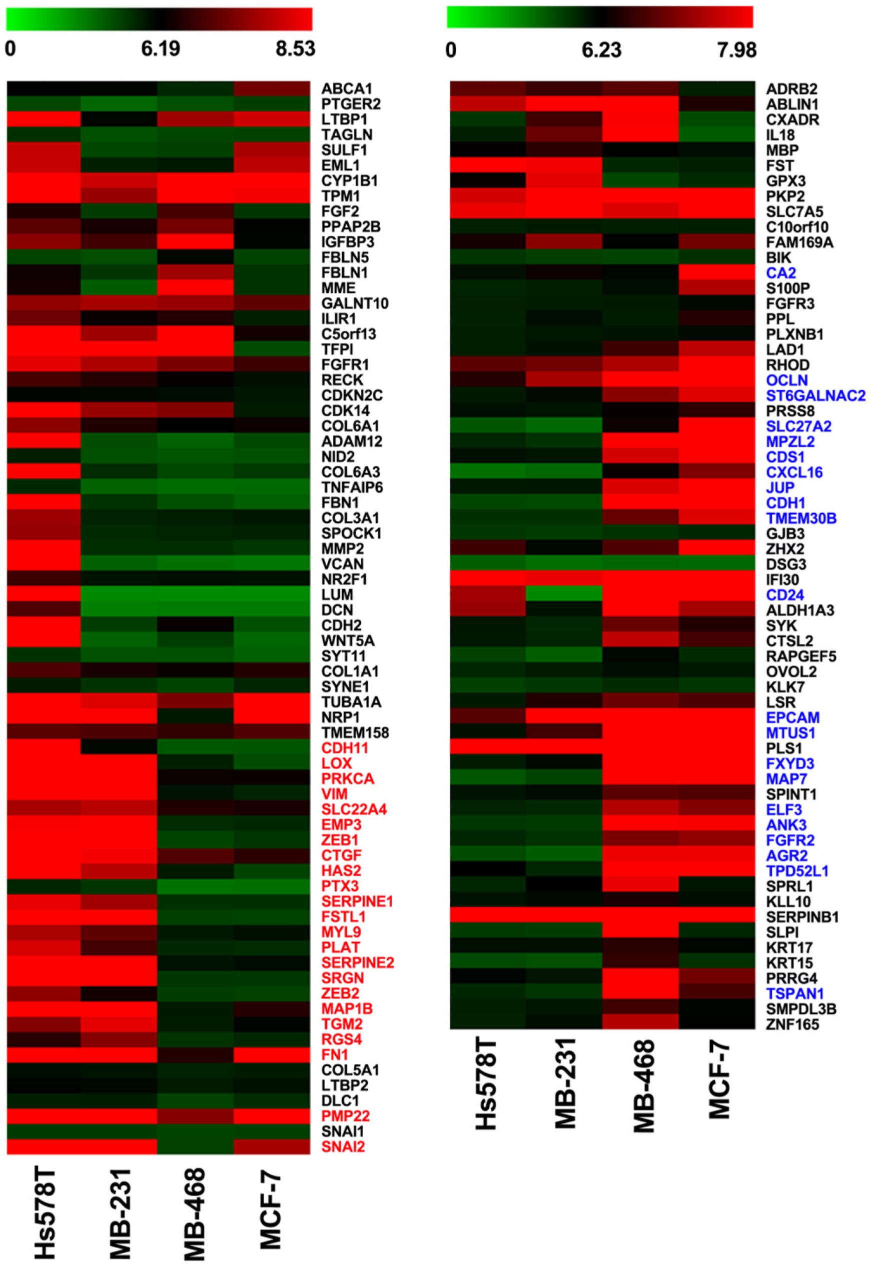

Supplementary Figure S2: Comparisons of the gene expression profiles of EMT-related genes in the malignant cell lines Hs578T and MB-231 with those of the less malignant cell lines MB-468 and MCF-7. The gene expression profiles of the upregulated EMT-related genes (left panel) and downregulated EMT-related genes (right panel) were detected by microarray assays of the four breast cancer cell lines.

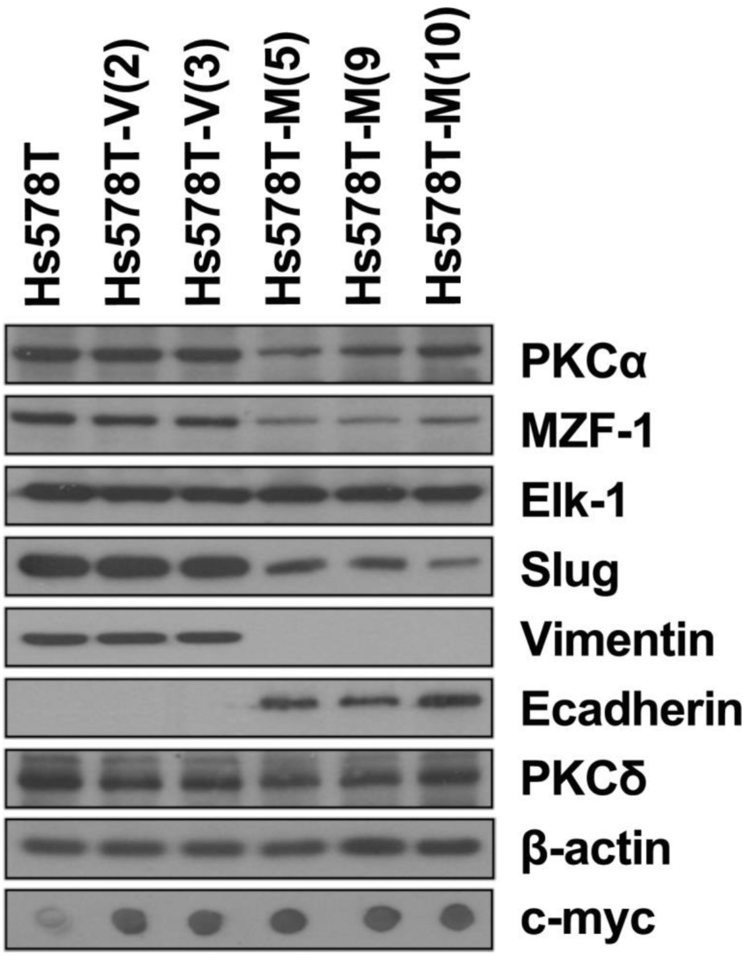

Supplementary Figure S3: Disrupting MZF-1/Elk-1 heterodimer formation by Elk-1$_{145\text{-}157}$ induces MET in Hs578T cells. Changes in protein levels in the parental and Elk-1$_{145\text{-}157}$-transfected stable Hs578T cells as detected by immunoblotting analysis. Low-passage cells were seeded at a density of 3×10^5 cells in 60 mm tissue culture dishes and then transfected with the Elk-1$_{145\text{-}157}$ construct (5 µg) using Lipofectamine 2000. After transfection for 6 h, the cells were washed three times in serum-free MEM and allowed to recover for 24 h in fresh medium. Stable clones were selected with geneticin (G418; 600 µg/ml) at 37°C for four weeks.

A

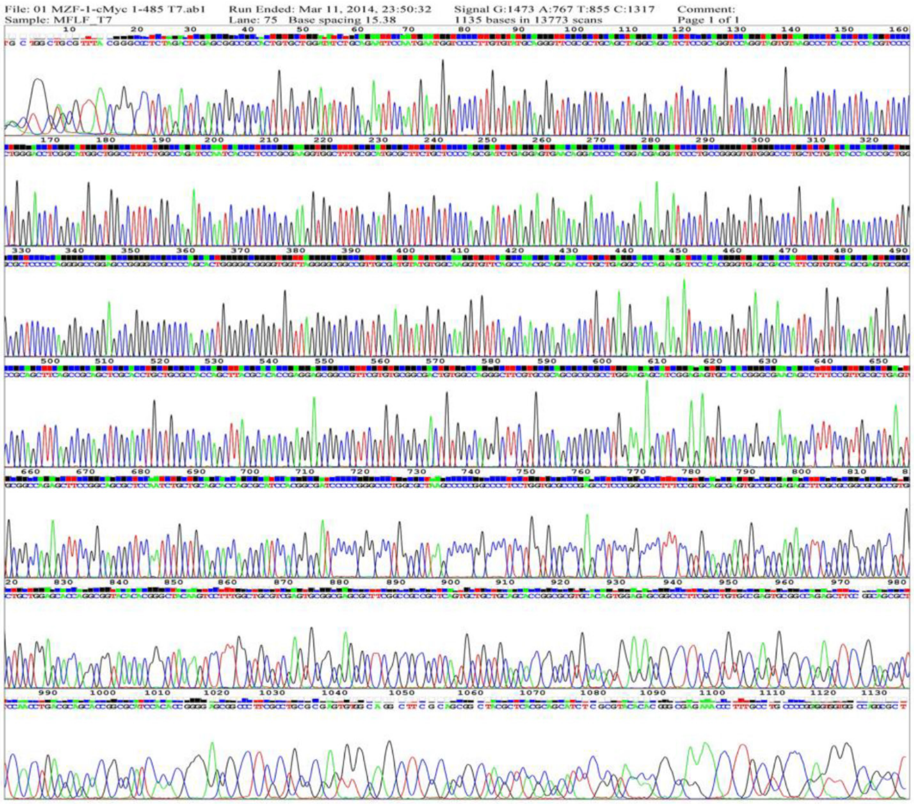

B

```
2014年6月29日                NCBI Blast:Nucleotide Sequence (880 letters)

Range 1: 10780 to 11591 GenBank Graphics                  Next Match   Previous Match
Score            Expect    Identities       Gaps          Strand
1495 bits(809)   0.0       811/812(99%)     0/812(0%)     Plus/Plus

Query  69     AATGAATGGTCCCCTTGTGTATGCAGGGTTCGCGCTGCAGCTAGGCAGCATCTCCGCAGG  128
              ||||||||||||||||||||||||||||||||||||||||||||||||||||||||||||
Sbjct  10780  AATGAATGGTCCCCTTGTGTATGCAGGGTTCGCGCTGCAGCTAGGCAGCATCTCCGCAGG  10839

Query  129    TCCAGGTAGTGTAAGCCCTCACCTCCACGTCCCCTGGGACCTCGGCATGGCTGGCCTTTC  188
              ||||||||||||||||||||||||||||||||||||||||||||||||||||||||||||
Sbjct  10840  TCCAGGTAGTGTAAGCCCTCACCTCCACGTCCCCTGGGACCTCGGCATGGCTGGCCTTTC  10899

Query  189    TGGCCAGATCCAATCACCCTCCCGCGAAGGTGGCTTTGCGCATGCGCTTCTGCTCCCCAG  248
              ||||||||||||||||||||||||||||||||||||||||||||||||||||||||||||
Sbjct  10900  TGGCCAGATCCAATCACCCTCCCGCGAAGGTGGCTTTGCGCATGCGCTTCTGCTCCCCAG  10959

Query  249    CGATCTGAGGAGTGAACAGGACCCCACGGACGAGGATCCCTGCCGGGGTGTGGGCCCTGC  308
              ||||||||||||||||||||||||||||||||||||||||||||||||||||||||||||
Sbjct  10960  CGATCTGAGGAGTGAACAGGACCCCACGGACGAGGATCCCTGCCGGGGTGTGGGCCCTGC  11019

Query  309    TCTGATCACCACCCGCTGGCGCTCCCCCAGGGGCCGGAGCCGGGGCCGCCCCAGCACTGG  368
              ||||||||||||||||||||||||||||||||||||||||||||||||||||||||||||
Sbjct  11020  TCTGATCACCACCCGCTGGCGCTCCCCCAGGGGCCGGAGCCGGGGCCGCCCCAGCACTGG  11079

Query  369    GGGCGGGGTGGTTAGGGGCGGCCGTTGCGATGTATGTGGCAAGGTGTTCAGCCAACGCAG  428
              ||||||||||||||||||||||||||||||||||||||||||||||||||||||||||||
Sbjct  11080  GGGCGGGGTGGTTAGGGGCGGCCGTTGCGATGTATGTGGCAAGGTGTTCAGCCAACGCAG  11139

Query  429    CAACCTGCTGAGGCACCAGAAGATCCACACGGGTGAGCGACCATTCGTGTGCAGCGAGTG  488
              ||||||||||||||||||||||||||||||||||||||||||||||||||||||||||||
Sbjct  11140  CAACCTGCTGAGGCACCAGAAGATCCACACGGGTGAGCGACCATTCGTGTGCAGCGAGTG  11199

Query  489    CGGCCGCAGCTTCAGCCGCAGCTCGCACCTGCTGCGCCACCAGCTTACGCACACCGAGGA  548
              ||||||||||||||||||||||||||||||||||||||||||||||||||||||||||||
Sbjct  11200  CGGCCGCAGCTTCAGCCGCAGCTCGCACCTGCTGCGCCACCAGCTTACGCACACCGAGGA  11259

Query  549    GCGGCCGTTCGTGTGCGGCGACTGTGGCCAGGGCTTCGTGCGCAGCGCGCGCCTGGAAGA  608
              ||||||||||||||||||||||||||||||||||||||||||||||||||||||||||||
Sbjct  11260  GCGGCCGTTCGTGTGCGGCGACTGTGGCCAGGGCTTCGTGCGCAGCGCGCGCCTGGAAGA  11319

Query  609    GCATCGGAGAGTGCACACGGGCGAACAGCCTTTCCGTTGCGCTGAGTGCGGCCAGAGCTT  668
              ||||||||||||||||||||||||||||||||||||||||||||||||||||||||||||
Sbjct  11320  GCATCGGAGAGTGCACACGGGCGAACAGCCTTTCCGTTGCGCTGAGTGCGGCCAGAGCTT  11379

Query  669    CCGGCAGCGCTCCAATCTGCTGCAGCACCAGCGCATCCACGGCGATcccccgggccctgg  728
              ||||||||||||||||||||||||||||||||||||||||||||||||||||||||||||
Sbjct  11380  CCGGCAGCGCTCCAATCTGCTGCAGCACCAGCGCATCCACGGCGATCCCCCGGGCCCTGG  11439

Query  729    cgctaagcccccggcccctcctggtgcgcccgagcctcccggcccctTTCCGTGCAGCGA  788
              ||||||||||||||||||||||||||||||||||||||||||||||||||||||||||||
Sbjct  11440  CGCTAAGCCCCCGGCCCCTCCTGGTGCGCCCGAGCCTCCCGGCCCCTTTCCGTGCAGCGA  11499

Query  789    GTGCCGCGAGAGCTTCGCGCGGCGCGCCGTGCTGCTGGAGCACCAGGCGGTACACACGGG  848
              ||||||||||||||||||||||||||||||||||||||||||||||||||||||||||||
Sbjct  11500  GTGCCGCGAGAGCTTCGCGCGGCGCGCCGTGCTGCTGGAGCACCAGGCGGTACACACGGG  11559

Query  849    CTACAAGTCCTTTGGCTGCGTCGAGTGCGGCG  880
              | ||||||||||||||||||||||||||||||
Sbjct  11560  CGACAAGTCCTTTGGCTGCGTCGAGTGCGGCG  11591
```

Supplementary Figure S4: The up sequence (10781 to 11591) of MZF-1 with a myc-tag (amino acids 1-485) (GenBank Accession No. AF 161886, nucleotides 10781 to 12235). (**A**) The sequencing data was present using the T7 primer (TAATACGACTCACTATAGGG) as initiator. (**B**) The "Query" (70 to 880) of sequence was aligned and identified by the National Center for Biotechnology Information (NCBI) BLAST (URL: http://blast.ncbi.nlm.nih.gov/Blast.cgi).

A

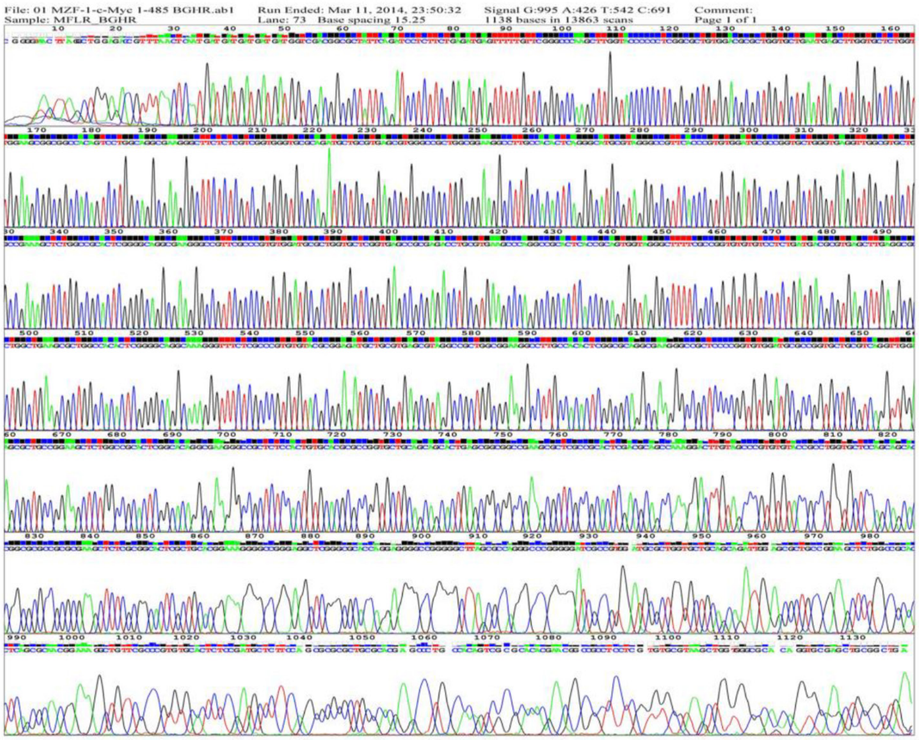

B

2014年6月29日 NCBI Blast:Nucleotide Sequence (880 letters)

Range 1: 11473 to 12235 GenBank Graphics Next Match Previous Match

Score	Expect	Identities	Gaps	Strand
1399 bits(757)	0.0	761/763(99%)	0/763(0%)	Plus/Minus

```
Query  118    CTCGGCGCTGTGGACGCGCTGGTGCTGAATGAGCTTGGTGCTCTGGTGGAAGCGGCGGCC  177
              ||||||||||||||||||||||||||||||||||||||||||||||||||||||||||||
Sbjct  12235  CTCGGCGCTGTGGACGCGCTGGTGCTGAATGAGCTTGGTGCTCTGGTGGAAGCGGCGGCC  12176

Query  178    ACAGTCCTGGCAGGCGAAGGGCTTCTCTCGTCGGTGGGTGCGCAGATGCTGCGTGAGCGT  237
              ||||||||||||||||||||||||||||||||||||||||||||||||||||||||||||
Sbjct  12175  ACAGTCCTGGCAGGCGAAGGGCTTCTCTCGTCGGTGGGTGCGCAGATGCTGCGTGAGCGT  12116

Query  238    GGGCCGCTGGCGGAAGGCCTTGCCACACTCAGGGCATGCGTAGGGCCGTTCACCCGTGTG  297
              ||||||||||||||||||||||||||||||||||||||||||||||||||||||||||||
Sbjct  12115  GGGCCGCTGGCGGAAGGCCTTGCCACACTCAGGGCATGCGTAGGGCCGTTCACCCGTGTG  12056

Query  298    GATGCGCCGGTGCTGGGTGAGGTTGGCGTGCTGCCGAAAGCTCTGGCCGCACTCGGGGCA  357
              ||||||||||||||||||||||||||||||||||||||||||||||||||||||||||||
Sbjct  12055  GATGCGCCGGTGCTGGGTGAGGTTGGCGTGCTGCCGAAAGCTCTGGCCGCACTCGGGGCA  11996

Query  358    GGCGAAGGGCCGTTCGCCCGTGTGGATGCGCTGGTGCTCGGTGAGCCGCGAGACCTGCGT  417
              ||||||||||||||||||||||||||||||||||||||||||||||||||||||||||||
Sbjct  11995  GGCGAAGGGCCGTTCGCCCGTGTGGATGCGCTGGTGCTCGGTGAGCCGCGAGACCTGCGT  11936

Query  418    GAAGCCCAGGCCGCACTCACCGCAGTGGTAGGGCTTTTCGCCGGTGTGTGTCCTCTGATG  477
              ||||||||||||||||||||||||||||||||||||||||||||||||||||||||||||
Sbjct  11935  GAAGCCCAGGCCGCACTCACCGCAGTGGTAGGGCTTTTCGCCGGTGTGTGTCCTCTGATG  11876

Query  478    ACGCGTGAGCTTGAGGCGCTGGCTGAAGCGCTGGCCACACTCGGGGCAGGCAAAGGGTTT  537
              ||||||||||||||||||||||||||||||||||||||||||||||||||||||||||||
Sbjct  11875  ACGCGTGAGCTTGAGGCGCTGGCTGAAGCGCTGGCCACACTCGGGGCAGGCAAAGGGTTT  11816

Query  538    CTCGCCCGTGTGTACGCGGAGATGCTGCGTGAGCGTAGGCCGCTGGCGGAAGGCCTTGCC  597
              ||||||||||||||||||||||||||||||||||||||||||||||||||||||||||||
Sbjct  11815  CTCGCCCGTGTGTACGCGGAGATGCTGCGTGAGCGTAGGCCGCTGGCGGAAGGCCTTGCC  11756

Query  598    ACACTCGGCGCAGGCGAAGGGCCGCTCCCCGGTGTGGATGCGCCGGTGCTGCGTCAGGTT  657
              ||||||||||||||||||||||||||||||||||||||||||||||||||||||||||||
Sbjct  11755  ACACTCGGCGCAGGCGAAGGGCCGCTCCCCGGTGTGGATGCGCCGGTGCTGCGTCAGGTT  11696

Query  658    GGAGCGCTGCCGGAAGCTCTGGCCGCACTCGGCACAGGCGAAGGGCCGCTCTCCACTGTG  717
              ||||||||||||||||||||||||||||||||||||||||||||||||||| ||||||||
Sbjct  11695  GGAGCGCTGCCGGAAGCTCTGGCCGCACTCGGCACAGGCGAAGGGCCGCTCGCCACTGTG  11636

Query  718    CACGCGCCGGTGCTGCAGCAGCACTGAGCGGCGGCCGAAGCGCTCGCCGCACTCGACGCA  777
              ||||||||||||||||||||||||||||||||||||||||||||||||||||||||||||
Sbjct  11635  CACGCGCCGGTGCTGCAGCAGCACTGAGCGGCGGCCGAAGCGCTCGCCGCACTCGACGCA  11576

Query  778    GCCAAAGGACTTGTAGCCCGTGTGTACCGCCTGGTGCTCCAGCAGCACGGCGCGCCGCGC  837
              |||||||||||||| |||||||||||||||||||||||||||||||||||||||||||||
Sbjct  11575  GCCAAAGGACTTGTCGCCCGTGTGTACCGCCTGGTGCTCCAGCAGCACGGCGCGCCGCGC  11516

Query  838    GAAGCTCTCGCGGCACTCGCTGCACGGAAAGGGGCCGGGAGGC  880
              |||||||||||||||||||||||||||||||||||||||||||
Sbjct  11515  GAAGCTCTCGCGGCACTCGCTGCACGGAAAGGGGCCGGGAGGC  11473
```

Supplementary Figure S5: The down sequence (11473 to 12235) of MZF-1 with a myc-tag (amino acids 1-485) (GenBank Accession No. AF 161886, nucleotides 10781 to 12235). (**A**) The sequencing data was present using the BGHR primer (TAGAAGGCACAGTCGAGGC) as initiator. (**B**) The "Query" (118 to 880) of sequence was aligned and identified by the NCBI BLAST. Two nucleotides, "T and A", in the sequencing data of MZF-1 with a myc-tag differ from the nucleotides "G and C" at nucleotides 11561 and 11644 in the GenBank AF 161886.1 sequence, respectively. The amino acid (tyrosine) encoded by the first mutation (TAC) is different from the amino acid (aspartate) encoded by the original sequence (GAC). However, the amino acid (glycine) encoded by the second mutation (GGA) is the same as that encoded by the original sequence (GGC).

A

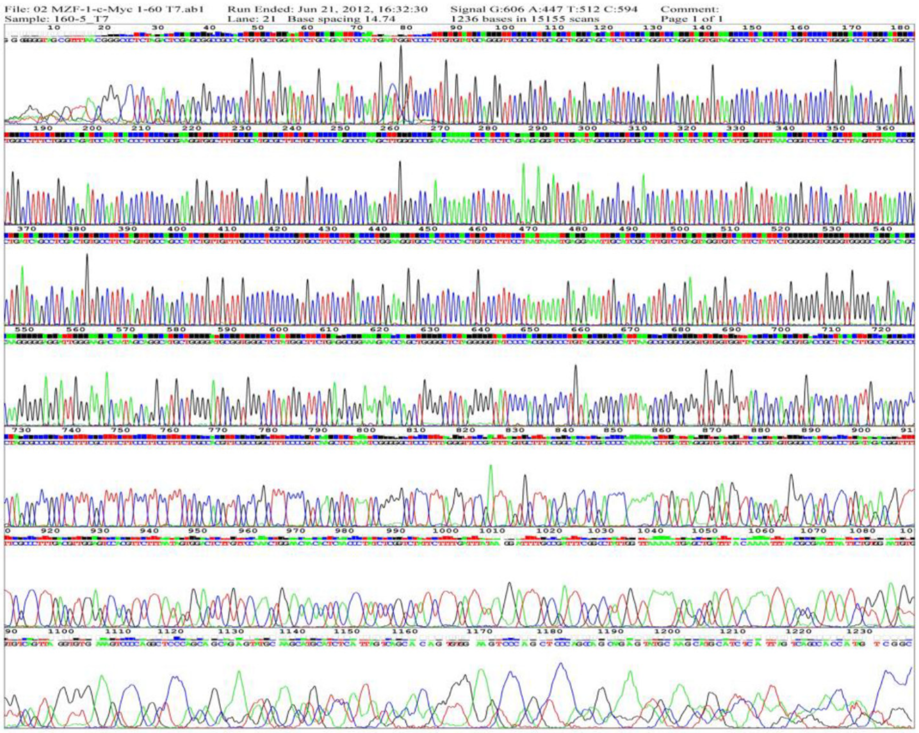

B

2014年6月29日 NCBI Blast:Nucleotide Sequence (320 letters)

Range 1: 10780 to 10960 GenBank Graphics Next Match Previous Match

Score	Expect	Identities	Gaps	Strand
335 bits(181)	2e-88	181/181(100%)	0/181(0%)	Plus/Plus

```
Query  72     AATGAATGGTCCCCTTGTGTATGCAGGGTTCGCGCTGCAGCTAGGCAGCATCTCCGCAGG  131
              ||||||||||||||||||||||||||||||||||||||||||||||||||||||||||||
Sbjct  10780  AATGAATGGTCCCCTTGTGTATGCAGGGTTCGCGCTGCAGCTAGGCAGCATCTCCGCAGG  10839

Query  132    TCCAGGTAGTGTAAGCCCTCACCTCCACGTCCCCTGGGACCTCGGCATGGCTGGCCTTTC  191
              ||||||||||||||||||||||||||||||||||||||||||||||||||||||||||||
Sbjct  10840  TCCAGGTAGTGTAAGCCCTCACCTCCACGTCCCCTGGGACCTCGGCATGGCTGGCCTTTC  10899

Query  192    TGGCCAGATCCAATCACCCTCCCGCGAAGGTGGCTTTGCGCATGCGCTTCTGCTCCCCAG  251
              ||||||||||||||||||||||||||||||||||||||||||||||||||||||||||||
Sbjct  10900  TGGCCAGATCCAATCACCCTCCCGCGAAGGTGGCTTTGCGCATGCGCTTCTGCTCCCCAG  10959

Query  252    C  252
              |
Sbjct  10960  C  10960
```

Supplementary Figure S6: MZF-1 with a myc-tag (amino acids 1-60) (GenBank Accession No. AF 161886, nucleotides 10781 to 10960). (**A**) The sequencing data was present using the T7 primer as initiator. (**B**) The "Query" (73 to 252) of sequence was aligned and identified by the NCBI BLAST.

A

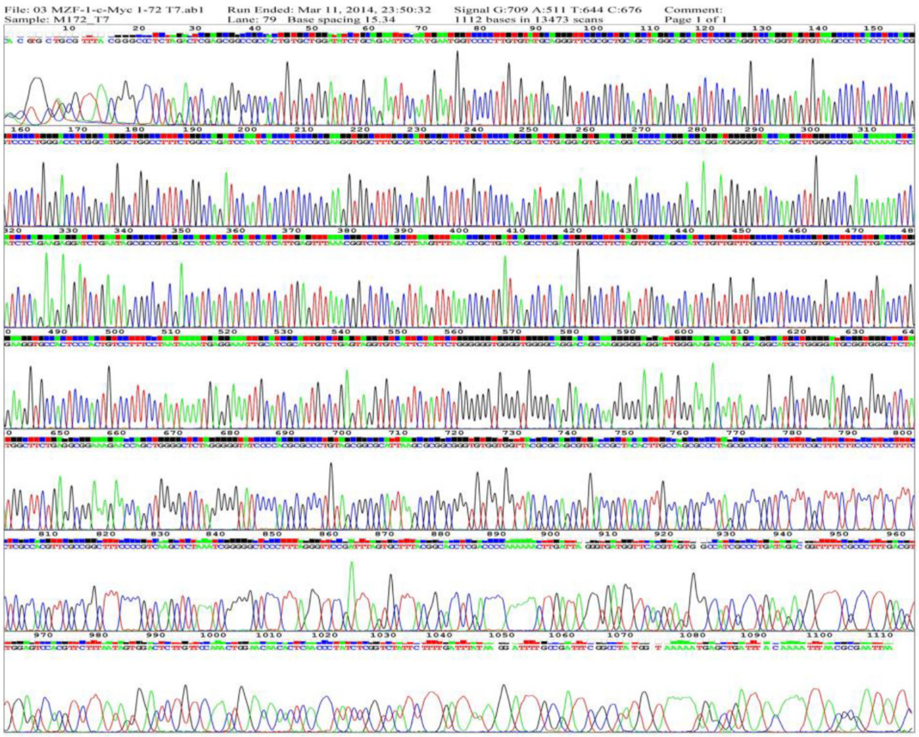

B

2014年6月29日 NCBI Blast:Nucleotide Sequence (400 letters)

Range 1: 10780 to 10996 GenBank Graphics Next Match Previous Match

Score	Expect	Identities	Gaps	Strand
401 bits(217)	2e-108	217/217(100%)	0/217(0%)	Plus/Plus

```
Query  69     AATGAATGGTCCCCTTGTGTATGCAGGGTTCGCGCTGCAGCTAGGCAGCATCTCCGCAGG  128
              ||||||||||||||||||||||||||||||||||||||||||||||||||||||||||||
Sbjct  10780  AATGAATGGTCCCCTTGTGTATGCAGGGTTCGCGCTGCAGCTAGGCAGCATCTCCGCAGG  10839

Query  129    TCCAGGTAGTGTAAGCCCTCACCTCCACGTCCCCTGGGACCTCGGCATGGCTGGCCTTTC  188
              ||||||||||||||||||||||||||||||||||||||||||||||||||||||||||||
Sbjct  10840  TCCAGGTAGTGTAAGCCCTCACCTCCACGTCCCCTGGGACCTCGGCATGGCTGGCCTTTC  10899

Query  189    TGGCCAGATCCAATCACCCTCCCGCGAAGGTGGCTTTGCGCATGCGCTTCTGCTCCCCAG  248
              ||||||||||||||||||||||||||||||||||||||||||||||||||||||||||||
Sbjct  10900  TGGCCAGATCCAATCACCCTCCCGCGAAGGTGGCTTTGCGCATGCGCTTCTGCTCCCCAG  10959

Query  249    CGATCTGAGGAGTGAACAGGACCCCACGGACGAGGAT  285
              |||||||||||||||||||||||||||||||||||||
Sbjct  10960  CGATCTGAGGAGTGAACAGGACCCCACGGACGAGGAT  10996
```

Supplementary Figure S7: MZF-1 with a myc-tag (amino acids 1-72) (GenBank Accession No. AF 161886, nucleotides 10781 to 10996). (**A**) The sequencing data was present using the T7 primer as initiator. (**B**) The "Query" (70 to 285) of sequence was aligned and identified by the NCBI BLAST.

A

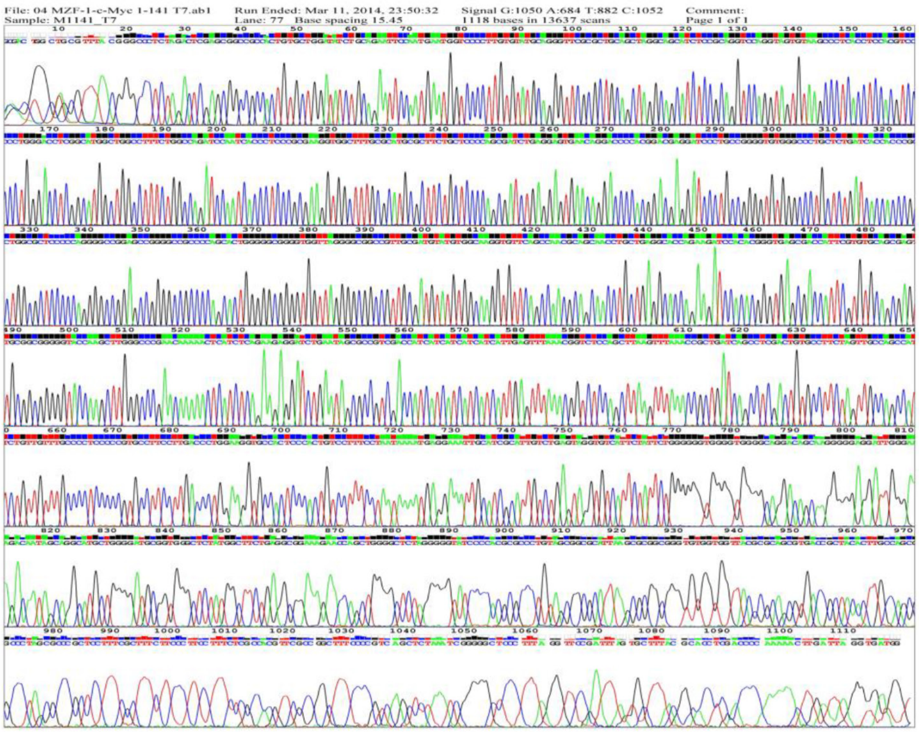

B

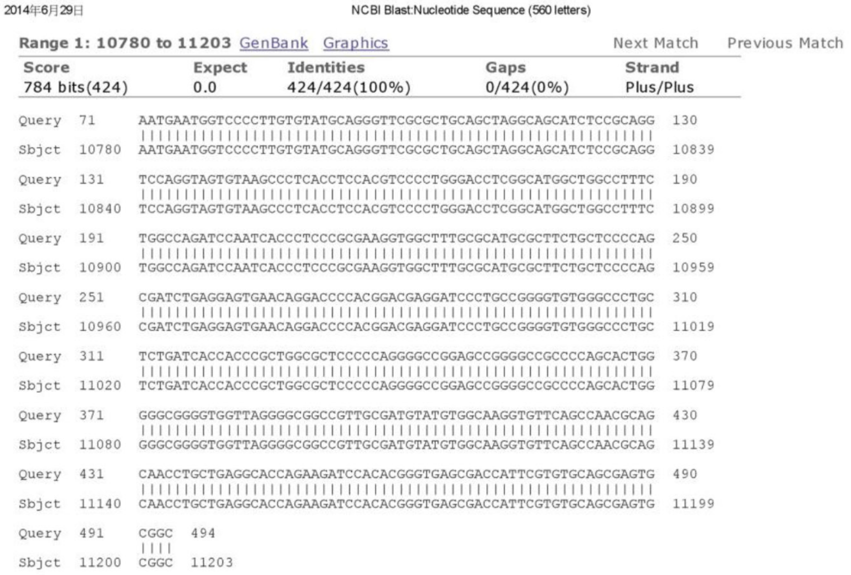

2014年6月29日 NCBI Blast:Nucleotide Sequence (560 letters)

Range 1: 10780 to 11203 GenBank Graphics Next Match Previous Match

Score	Expect	Identities	Gaps	Strand
784 bits(424)	0.0	424/424(100%)	0/424(0%)	Plus/Plus

```
Query  71     AATGAATGGTCCCCTTGTGTATGCAGGGTTCGCGCTGCAGCTAGGCAGCATCTCCGCAGG  130
              ||||||||||||||||||||||||||||||||||||||||||||||||||||||||||||
Sbjct  10780  AATGAATGGTCCCCTTGTGTATGCAGGGTTCGCGCTGCAGCTAGGCAGCATCTCCGCAGG  10839

Query  131    TCCAGGTAGTGTAAGCCCTCACCTCCACGTCCCCTGGGACCTCGGCATGGCTGGCCTTTC  190
              ||||||||||||||||||||||||||||||||||||||||||||||||||||||||||||
Sbjct  10840  TCCAGGTAGTGTAAGCCCTCACCTCCACGTCCCCTGGGACCTCGGCATGGCTGGCCTTTC  10899

Query  191    TGGCCAGATCCAATCACCCTCCCGCGAAGGTGGCTTTGCGCATGCGCTTCTGCTCCCCAG  250
              ||||||||||||||||||||||||||||||||||||||||||||||||||||||||||||
Sbjct  10900  TGGCCAGATCCAATCACCCTCCCGCGAAGGTGGCTTTGCGCATGCGCTTCTGCTCCCCAG  10959

Query  251    CGATCTGAGGAGTGAACAGGACCCCACGGACGAGGATCCCTGCCGGGGTGTGGGCCCTGC  310
              ||||||||||||||||||||||||||||||||||||||||||||||||||||||||||||
Sbjct  10960  CGATCTGAGGAGTGAACAGGACCCCACGGACGAGGATCCCTGCCGGGGTGTGGGCCCTGC  11019

Query  311    TCTGATCACCACCCGCTGGCGCTCCCCCAGGGGCCGGAGCCGGGGCCGCCCCAGCACTGG  370
              ||||||||||||||||||||||||||||||||||||||||||||||||||||||||||||
Sbjct  11020  TCTGATCACCACCCGCTGGCGCTCCCCCAGGGGCCGGAGCCGGGGCCGCCCCAGCACTGG  11079

Query  371    GGGCGGGGTGGTTAGGGGCGGCCGTTGCGATGTATGTGGCAAGGTGTTCAGCCAACGCAG  430
              ||||||||||||||||||||||||||||||||||||||||||||||||||||||||||||
Sbjct  11080  GGGCGGGGTGGTTAGGGGCGGCCGTTGCGATGTATGTGGCAAGGTGTTCAGCCAACGCAG  11139

Query  431    CAACCTGCTGAGGCACCAGAAGATCCACACGGGTGAGCGACCATTCGTGTGCAGCGAGTG  490
              ||||||||||||||||||||||||||||||||||||||||||||||||||||||||||||
Sbjct  11140  CAACCTGCTGAGGCACCAGAAGATCCACACGGGTGAGCGACCATTCGTGTGCAGCGAGTG  11199

Query  491    CGGC  494
              ||||
Sbjct  11200  CGGC  11203
```

Supplementary Figure S8: MZF-1 with a myc-tag (amino acids 1-141) (GenBank Accession No. AF 161886, nucleotides 10781 to 11203) (**A**) The sequencing data was present using the T7 primer as initiator. (**B**) The "Query" (72 to 494) of sequence was aligned and identified by the NCBI BLAST.

A

B

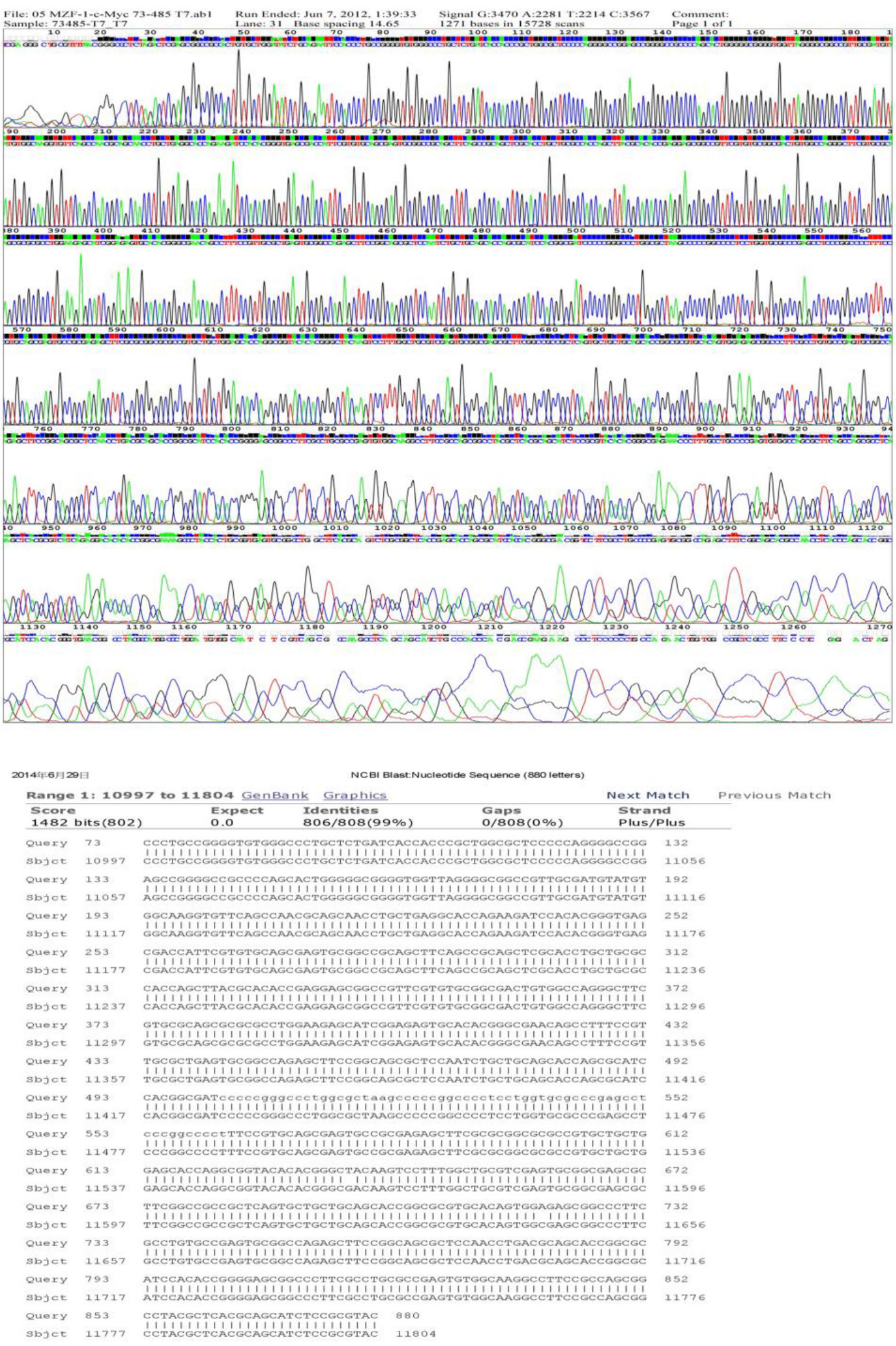

Supplementary Figure S9: The up sequence (10997 to 11643) of MZF-1 with a myc-tag (amino acids 73-485) (GenBank Accession No. AF 161886, nucleotides 10997 to 12235). (**A**) The sequencing data was present using the T7 primer as initiator. (**B**) The "Query" (73 to 719) of sequence was aligned and identified by the NCBI BLAST.

A

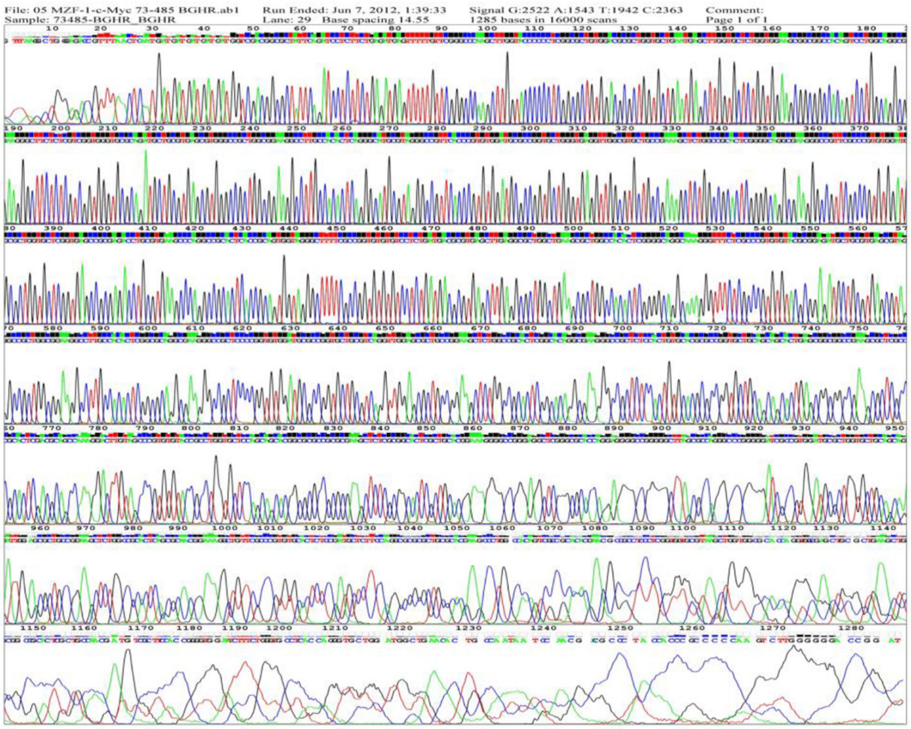

B

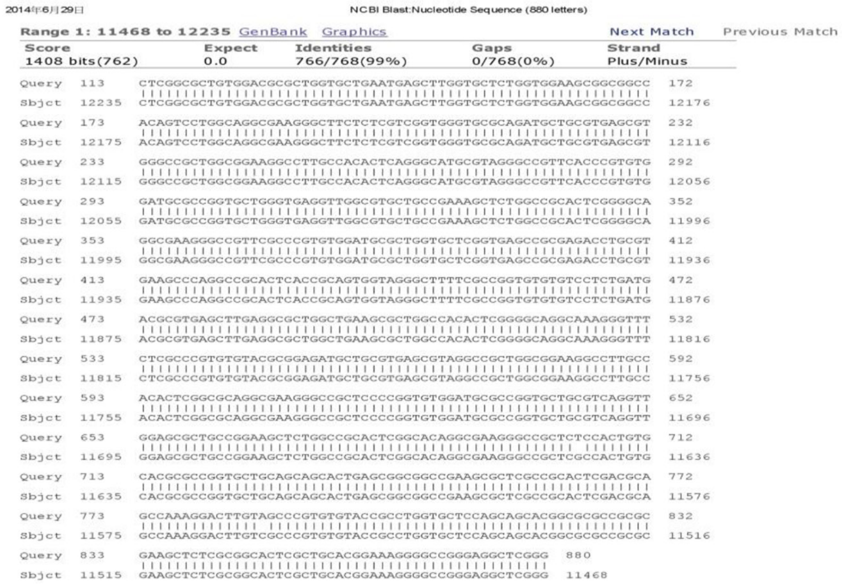

2014年6月29日 NCBI Blast:Nucleotide Sequence (880 letters)

Range 1: 11468 to 12235 GenBank Graphics Next Match Previous Match

Score	Expect	Identities	Gaps	Strand
1408 bits(762)	0.0	766/768(99%)	0/768(0%)	Plus/Minus

```
Query  113    CTCGGCGCTGTGGACGCGCTGGTGCTGAATGAGCTTGGTGCTCTGGTGGAAGCGGCGGCC  172
              ||||||||||||||||||||||||||||||||||||||||||||||||||||||||||||
Sbjct  12235  CTCGGCGCTGTGGACGCGCTGGTGCTGAATGAGCTTGGTGCTCTGGTGGAAGCGGCGGCC  12176

Query  173    ACAGTCCTGGCAGGCGAAGGGCTTCTCTCGTCGGTGGGTGCGCAGATGCTGCGTGAGCGT  232
              ||||||||||||||||||||||||||||||||||||||||||||||||||||||||||||
Sbjct  12175  ACAGTCCTGGCAGGCGAAGGGCTTCTCTCGTCGGTGGGTGCGCAGATGCTGCGTGAGCGT  12116

Query  233    GGGCCGCTGGCGGAAGGCCTTGCCACACTCAGGGCATGCGTAGGGCCGTTCACCCGTGTG  292
              ||||||||||||||||||||||||||||||||||||||||||||||||||||||||||||
Sbjct  12115  GGGCCGCTGGCGGAAGGCCTTGCCACACTCAGGGCATGCGTAGGGCCGTTCACCCGTGTG  12056

Query  293    GATGCGCCGGTGCTGGGTGAGGTTGGCGTGCTGCCGAAAGCTCTGGCCGCACTCGGGGCA  352
              ||||||||||||||||||||||||||||||||||||||||||||||||||||||||||||
Sbjct  12055  GATGCGCCGGTGCTGGGTGAGGTTGGCGTGCTGCCGAAAGCTCTGGCCGCACTCGGGGCA  11996

Query  353    GGCGAAGGGCCGTTCGCCCGTGTGGATGCGCTGGTGCTCGGTGAGCCGCGAGACCTGCGT  412
              ||||||||||||||||||||||||||||||||||||||||||||||||||||||||||||
Sbjct  11995  GGCGAAGGGCCGTTCGCCCGTGTGGATGCGCTGGTGCTCGGTGAGCCGCGAGACCTGCGT  11936

Query  413    GAAGCCCAGGCCGCACTCACCGCAGTGGTAGGGCTTTTCGCCGGTGTGTGTCCTCTGATG  472
              ||||||||||||||||||||||||||||||||||||||||||||||||||||||||||||
Sbjct  11935  GAAGCCCAGGCCGCACTCACCGCAGTGGTAGGGCTTTTCGCCGGTGTGTGTCCTCTGATG  11876

Query  473    ACGCGTGAGCTTGAGGCGCTGGCTGAAGCGCTGGCCACACTCGGGGCAGGCAAAGGGTTT  532
              ||||||||||||||||||||||||||||||||||||||||||||||||||||||||||||
Sbjct  11875  ACGCGTGAGCTTGAGGCGCTGGCTGAAGCGCTGGCCACACTCGGGGCAGGCAAAGGGTTT  11816

Query  533    CTCGCCCGTGTGTACGCGGAGATGCTGCGTGAGCGTAGGCCGCTGGCGGAAGGCCTTGCC  592
              ||||||||||||||||||||||||||||||||||||||||||||||||||||||||||||
Sbjct  11815  CTCGCCCGTGTGTACGCGGAGATGCTGCGTGAGCGTAGGCCGCTGGCGGAAGGCCTTGCC  11756

Query  593    ACACTCGGCGCAGGCGAAGGGCCGCTCCCCGGTGTGGATGCGCCGGTGCTGCGTCAGGTT  652
              ||||||||||||||||||||||||||||||||||||||||||||||||||||||||||||
Sbjct  11755  ACACTCGGCGCAGGCGAAGGGCCGCTCCCCGGTGTGGATGCGCCGGTGCTGCGTCAGGTT  11696

Query  653    GGAGCGCTGCCGGAAGCTCTGGCCGCACTCGGCACAGGCGAAGGGCCGCTCTCCACTGTG  712
              |||||||||||||||||||||||||||||||||||||||||||||||||||| |||||||
Sbjct  11695  GGAGCGCTGCCGGAAGCTCTGGCCGCACTCGGCACAGGCGAAGGGCCGCTCGCCACTGTG  11636

Query  713    CACGCGCCGGTGCTGCAGCAGCACTGAGCGGCGGCCGAAGCGCTCGCCGCACTCGACGCA  772
              ||||||||||||||||||||||||||||||||||||||||||||||||||||||||||||
Sbjct  11635  CACGCGCCGGTGCTGCAGCAGCACTGAGCGGCGGCCGAAGCGCTCGCCGCACTCGACGCA  11576

Query  773    GCCAAAGGACTTGTAGCCCGTGTGTACCGCCTGGTGCTCCAGCAGCACGGCGCGCCGCGC  832
              |||||||||||||| |||||||||||||||||||||||||||||||||||||||||||||
Sbjct  11575  GCCAAAGGACTTGTCGCCCGTGTGTACCGCCTGGTGCTCCAGCAGCACGGCGCGCCGCGC  11516

Query  833    GAAGCTCTCGCGGCACTCGCTGCACGGAAAGGGGCCGGGAGGCTCGGG  880
              ||||||||||||||||||||||||||||||||||||||||||||||||
Sbjct  11515  GAAGCTCTCGCGGCACTCGCTGCACGGAAAGGGGCCGGGAGGCTCGGG  11468
```

Supplementary Figure S10: The down sequence (11708 to 12235) of MZF-1 with a myc-tag (amino acids 73-485) (GenBank Accession No. AF 161886, nucleotides 10997 to 12235). (**A**) The sequencing data was present using the BGHR primer as initiator. (**B**) The "Query" (113 to 640) of sequence was aligned and identified by the NCBI BLAST.

A

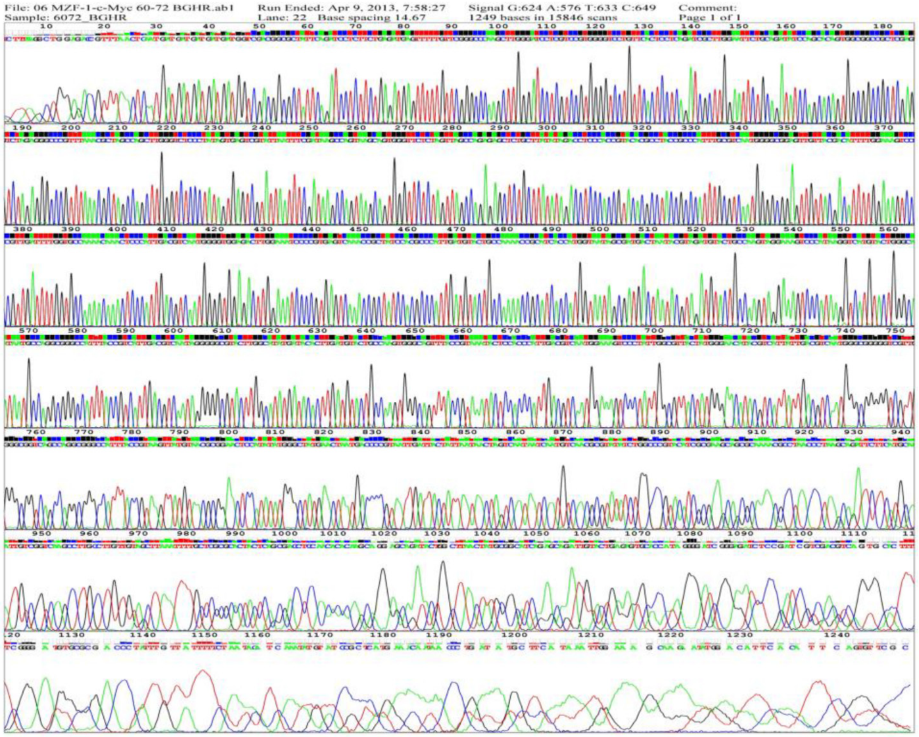

B

2014年6月30日 NCBI Blast:Nucleotide Sequence (160 letters)

Range 1: 10958 to 10999 GenBank Graphics Next Match Previous Match

Score	Expect	Identities	Gaps	Strand
78.7 bits(42)	2e-11	42/42(100%)	0/42(0%)	Plus/Minus

```
Query  104    GGGATCCTCGTCCGTGGGGTCCTGTTCACTCCTCAGATCGCT  145
              ||||||||||||||||||||||||||||||||||||||||||
Sbjct  10999  GGGATCCTCGTCCGTGGGGTCCTGTTCACTCCTCAGATCGCT  10958
```

Supplementary Figure S11: MZF-1 with a myc-tag (amino acids 60-72) (GenBank Accession No. AF 161886, nucleotides 10958 to 10996). (**A**) The sequencing data was present using the BGHR primer as initiator. (**B**) The "Query" (107 to 145) of sequence was aligned and identified by the NCBI BLAST.

A

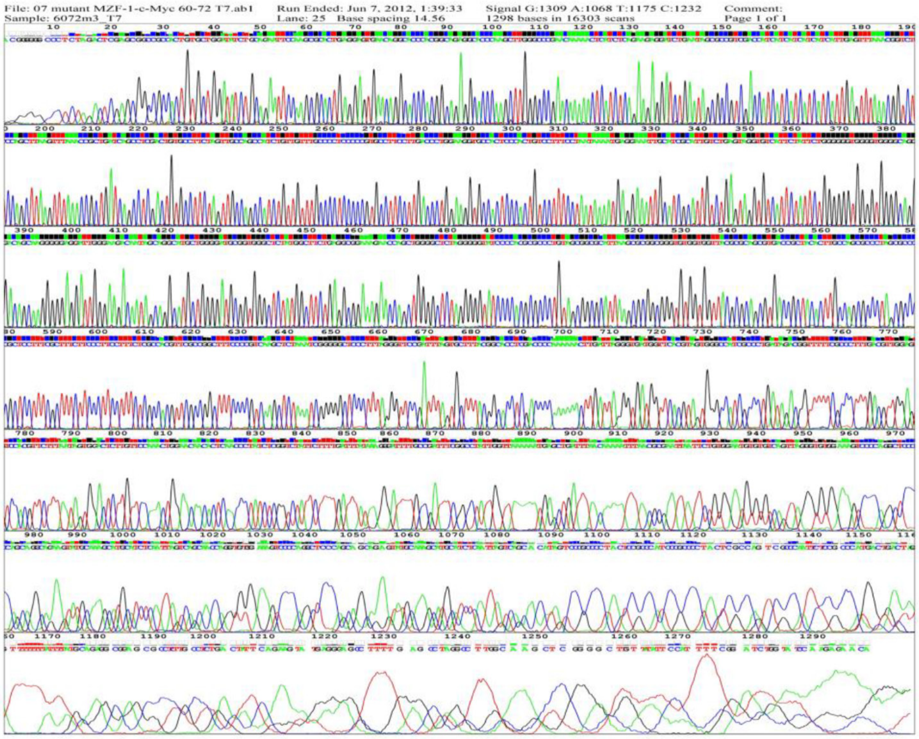

B

```
Query  59     AGCGCACTGAGGAGTGAACAGGCACCCACGGCAGAGGCA  97
              ||||  ||||||||||||||||  |||||||  ||||
Sbjct  10958  AGCGATCTGAGGAGTGAACAGGACCCCACGGACGAGGAT  10996
```

Supplementary Figure S12: Mutant MZF-1 with a myc-tag (amino acids 60-72) (GenBank Accession No. AF 161886, nucleotides 10958 to 10996). (**A**) The sequencing data was present using the T7 primer as initiator. (**B**) The "Query" (59 to 97) of sequence was aligned and identified by the NCBI BLAST.

A

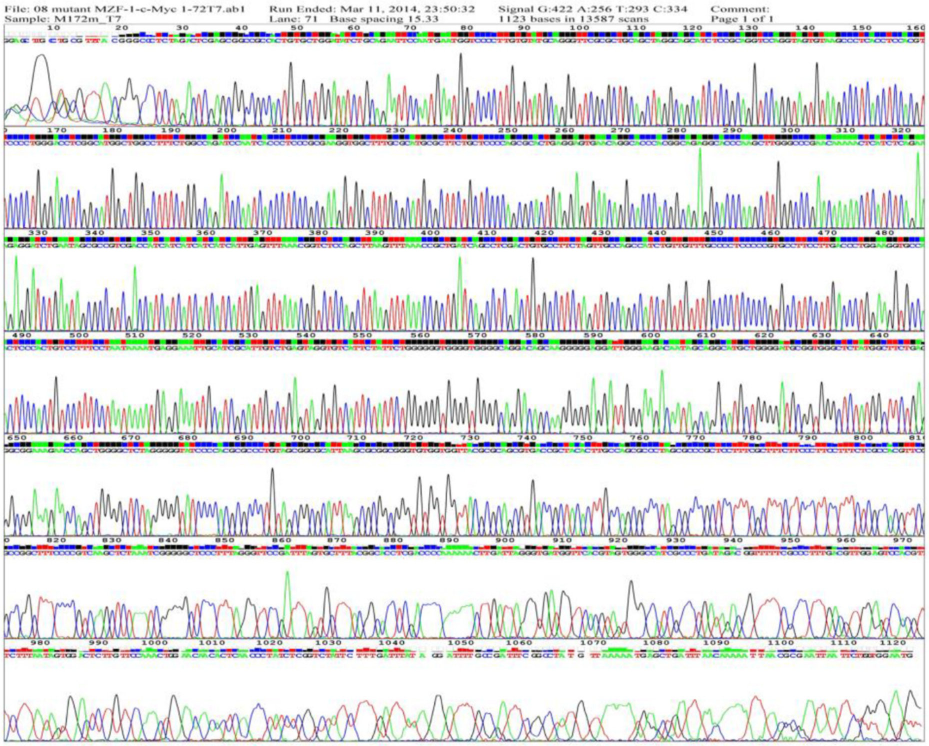

B

2014年6月30日　　NCBI Blast:Nucleotide Sequence (320 letters)

Range 1: 10780 to 10988 GenBank Graphics　　Next Match　Previous Match

Score	Expect	Identities	Gaps	Strand
364 bits(197)	2e-97	205/209(98%)	0/209(0%)	Plus/Plus

```
Query  72     AATGAATGGTCCCCTTGTGTATGCAGGGTTCGCGCTGCAGCTAGGCAGCATCTCCGCAGG  131
              ||||||||||||||||||||||||||||||||||||||||||||||||||||||||||||
Sbjct  10780  AATGAATGGTCCCCTTGTGTATGCAGGGTTCGCGCTGCAGCTAGGCAGCATCTCCGCAGG  10839

Query  132    TCCAGGTAGTGTAAGCCCTCACCTCCACGTCCCCTGGGACCTCGGCATGGCTGGCCTTTC  191
              ||||||||||||||||||||||||||||||||||||||||||||||||||||||||||||
Sbjct  10840  TCCAGGTAGTGTAAGCCCTCACCTCCACGTCCCCTGGGACCTCGGCATGGCTGGCCTTTC  10899

Query  192    TGGCCAGATCCAATCACCCTCCCGCGAAGGTGGCTTTGCGCATGCGCTTCTGCTCCCCAG  251
              ||||||||||||||||||||||||||||||||||||||||||||||||||||||||||||
Sbjct  10900  TGGCCAGATCCAATCACCCTCCCGCGAAGGTGGCTTTGCGCATGCGCTTCTGCTCCCCAG  10959

Query  252    CGCACTGAGGAGTGAACAGGCACCCACGG  280      281 CAGAGGCA 288
              ||  |||||||||||||||   |||||||                 | | | | |
Sbjct  10960  CGATCTGAGGAGTGAACAGGACCCCACGG  10988    10989 ACGAGGAT 10996
```

Supplementary Figure S13: Mutant MZF-1 with a myc-tag (amino acids 1-72) (GenBank Accession No. AF 161886, nucleotides 10781 to 10996). (**A**) The sequencing data was present using the T7 primer as initiator. (**B**) The “Query” (73 to 288) of sequence was aligned and identified by the NCBI BLAST.

A

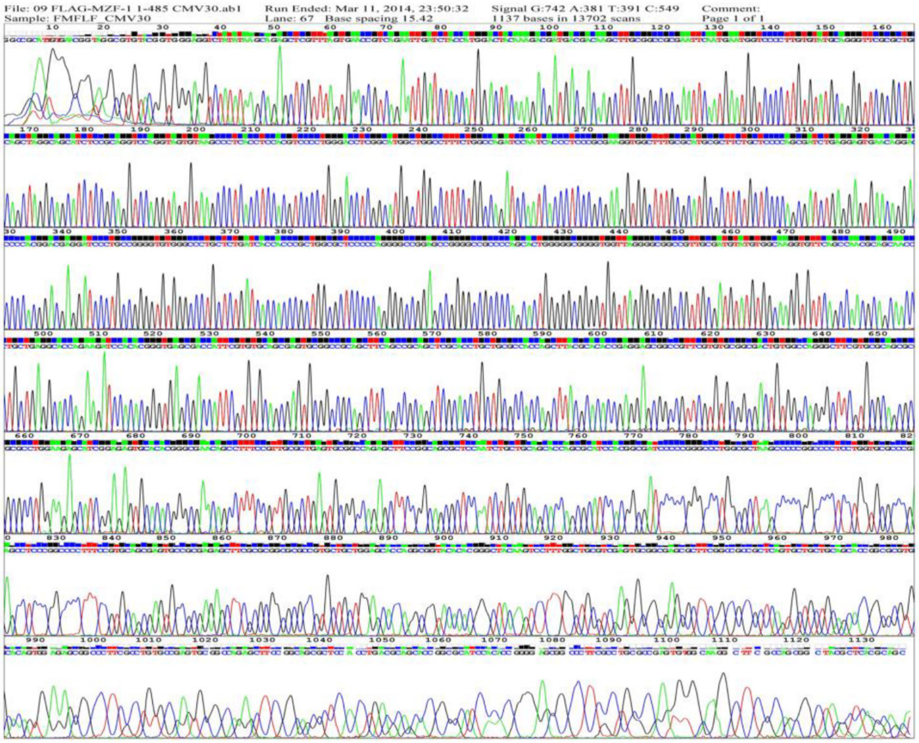

B

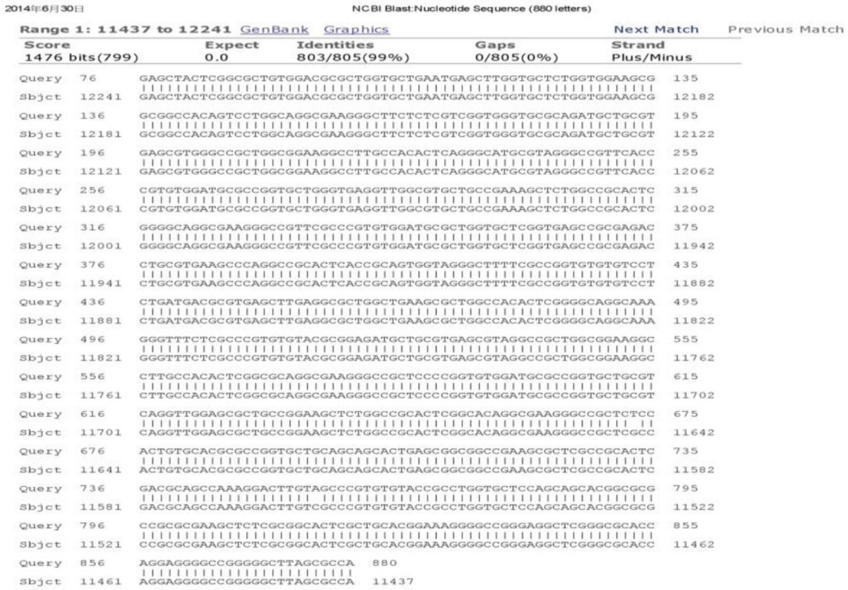

2014年6月30日 NCBI Blast:Nucleotide Sequence (880 letters)

Range 1: 11437 to 12241 GenBank Graphics Next Match Previous Match

Score	Expect	Identities	Gaps	Strand
1476 bits(799)	0.0	803/805(99%)	0/805(0%)	Plus/Minus

```
Query  76     GAGCTACTCGGCGCTGTGGACGCGCTGGTGCTGAATGAGCTTGGTGCTCTGGTGGAAGCG  135
              ||||||||||||||||||||||||||||||||||||||||||||||||||||||||||||
Sbjct  12241  GAGCTACTCGGCGCTGTGGACGCGCTGGTGCTGAATGAGCTTGGTGCTCTGGTGGAAGCG  12182

Query  136    GCGGCCACAGTCCTGGCAGGCGAAGGGCTTCTCTCGTCGGTGGGTGCGCAGATGCTGCGT  195
              ||||||||||||||||||||||||||||||||||||||||||||||||||||||||||||
Sbjct  12181  GCGGCCACAGTCCTGGCAGGCGAAGGGCTTCTCTCGTCGGTGGGTGCGCAGATGCTGCGT  12122

Query  196    GAGCGTGGGCCGCTGGCGGAAGGCCTTGCCACACTCAGGGCATGCGTAGGGCCGTTCACC  255
              ||||||||||||||||||||||||||||||||||||||||||||||||||||||||||||
Sbjct  12121  GAGCGTGGGCCGCTGGCGGAAGGCCTTGCCACACTCAGGGCATGCGTAGGGCCGTTCACC  12062

Query  256    CGTGTGGATGCGCCGGTGCTGGGTGAGGTTGGCGTGCTGCCGAAAGCTCTGGCCGCACTC  315
              ||||||||||||||||||||||||||||||||||||||||||||||||||||||||||||
Sbjct  12061  CGTGTGGATGCGCCGGTGCTGGGTGAGGTTGGCGTGCTGCCGAAAGCTCTGGCCGCACTC  12002

Query  316    GGGGCAGGCGAAGGGCCGTTCGCCCGTGTGGATGCGCTGGTGCTCGGTGAGCCGCGAGAC  375
              ||||||||||||||||||||||||||||||||||||||||||||||||||||||||||||
Sbjct  12001  GGGGCAGGCGAAGGGCCGTTCGCCCGTGTGGATGCGCTGGTGCTCGGTGAGCCGCGAGAC  11942

Query  376    CTGCGTGAAGCCCAGGCCGCACTCACCGCAGTGGTAGGGCTTTTCGCCGGTGTGTGTCCT  435
              ||||||||||||||||||||||||||||||||||||||||||||||||||||||||||||
Sbjct  11941  CTGCGTGAAGCCCAGGCCGCACTCACCGCAGTGGTAGGGCTTTTCGCCGGTGTGTGTCCT  11882

Query  436    CTGATGACGCGTGAGCTTGAGGCGCTGGCTGAAGCGCTGGCCACACTCGGGGCAGGCAAA  495
              ||||||||||||||||||||||||||||||||||||||||||||||||||||||||||||
Sbjct  11881  CTGATGACGCGTGAGCTTGAGGCGCTGGCTGAAGCGCTGGCCACACTCGGGGCAGGCAAA  11822

Query  496    GGGTTTCTCGCCCGTGTGTACGCGGAGATGCTGCGTGAGCGTAGGCCGCTGGCGGAAGGC  555
              ||||||||||||||||||||||||||||||||||||||||||||||||||||||||||||
Sbjct  11821  GGGTTTCTCGCCCGTGTGTACGCGGAGATGCTGCGTGAGCGTAGGCCGCTGGCGGAAGGC  11762

Query  556    CTTGCCACACTCGGCGCAGGCGAAGGGCCGCTCCCCGGTGTGGATGCGCCGGTGCTGCGT  615
              ||||||||||||||||||||||||||||||||||||||||||||||||||||||||||||
Sbjct  11761  CTTGCCACACTCGGCGCAGGCGAAGGGCCGCTCCCCGGTGTGGATGCGCCGGTGCTGCGT  11702

Query  616    CAGGTTGGAGCGCTGCCGGAAGCTCTGGCCGCACTCGGCACAGGCGAAGGGCCGCTCTCC  675
              ||||||||||||||||||||||||||||||||||||||||||||||||||||||||| ||
Sbjct  11701  CAGGTTGGAGCGCTGCCGGAAGCTCTGGCCGCACTCGGCACAGGCGAAGGGCCGCTCGCC  11642

Query  676    ACTGTGCACGCGCCGGTGCTGCAGCAGCACTGAGCGGCGGCCGAAGCGCTCGCCGCACTC  735
              ||||||||||||||||||||||||||||||||||||||||||||||||||||||||||||
Sbjct  11641  ACTGTGCACGCGCCGGTGCTGCAGCAGCACTGAGCGGCGGCCGAAGCGCTCGCCGCACTC  11582

Query  736    GACGCAGCCAAAGGACTTGTAGCCCGTGTGTACCGCCTGGTGCTCCAGCAGCACGGCGCG  795
              |||||||||||||||||||| |||||||||||||||||||||||||||||||||||||||
Sbjct  11581  GACGCAGCCAAAGGACTTGTCGCCCGTGTGTACCGCCTGGTGCTCCAGCAGCACGGCGCG  11522

Query  796    CCGCGCGAAGCTCTCGCGGCACTCGCTGCACGGAAAGGGGCCGGGAGGCTCGGGCGCACC  855
              ||||||||||||||||||||||||||||||||||||||||||||||||||||||||||||
Sbjct  11521  CCGCGCGAAGCTCTCGCGGCACTCGCTGCACGGAAAGGGGCCGGGAGGCTCGGGCGCACC  11462

Query  856    AGGAGGGGCCGGGGGCTTAGCGCCA  880
              |||||||||||||||||||||||||
Sbjct  11461  AGGAGGGGCCGGGGGCTTAGCGCCA  11437
```

Supplementary Figure S14: The up sequence (10781 to 11531) of MZF-1 with a FLAG-tag (amino acids 1-485) (GenBank Accession No. AF 161886, nucleotides 10781 to 12235). (**A**) The sequencing data was present using the CMV30 primer (AATGTCGTAATAACCCCGCCCCGTTGACGC) as initiator. (**B**) The “Query” (130 to 880) of sequence was aligned and identified by the NCBI BLAST.

A

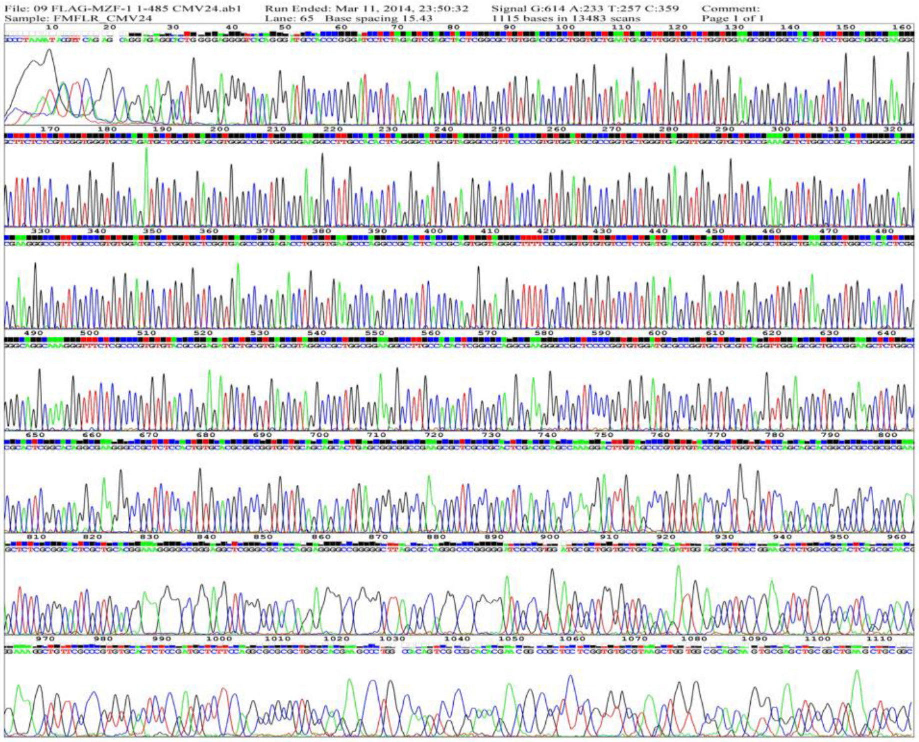

B

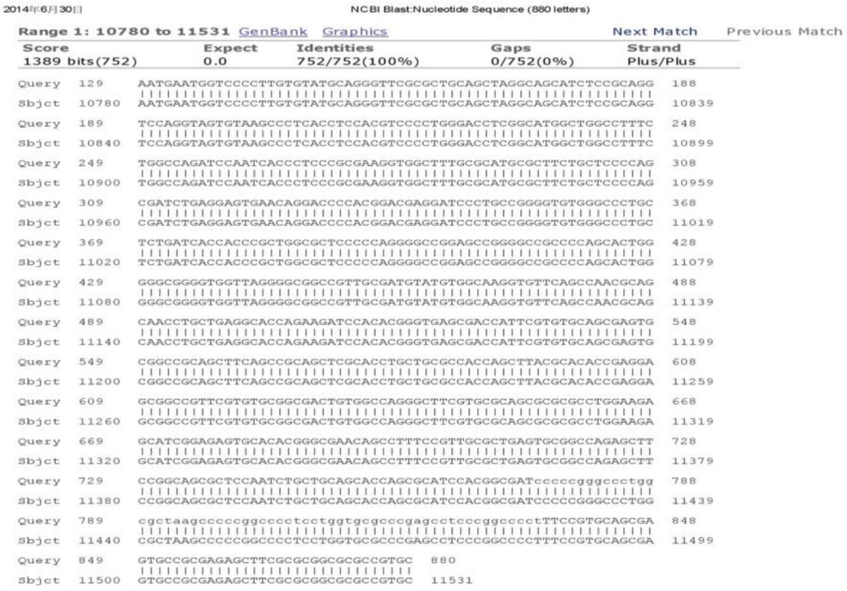

```
2014年6月30日                NCBI Blast:Nucleotide Sequence (880 letters)

Range 1: 10780 to 11531 GenBank Graphics                 Next Match   Previous Match
Score            Expect   Identities        Gaps         Strand
1389 bits(752)   0.0      752/752(100%)     0/752(0%)    Plus/Plus

Query  129    AATGAATGGTCCCCTTGTGTATGCAGGGTTCGCGCTGCAGCTAGGCAGCATCTCCGCAGG  188
              ||||||||||||||||||||||||||||||||||||||||||||||||||||||||||||
Sbjct  10780  AATGAATGGTCCCCTTGTGTATGCAGGGTTCGCGCTGCAGCTAGGCAGCATCTCCGCAGG  10839

Query  189    TCCAGGTAGTGTAAGCCCTCACCTCCACGTCCCCTGGGACCTCGGCATGGCTGGCCTTTC  248
              ||||||||||||||||||||||||||||||||||||||||||||||||||||||||||||
Sbjct  10840  TCCAGGTAGTGTAAGCCCTCACCTCCACGTCCCCTGGGACCTCGGCATGGCTGGCCTTTC  10899

Query  249    TGGCCAGATCCAATCACCCTCCCGCGAAGGTGGCTTTGCGCATGCGCTTCTGCTCCCCAG  308
              ||||||||||||||||||||||||||||||||||||||||||||||||||||||||||||
Sbjct  10900  TGGCCAGATCCAATCACCCTCCCGCGAAGGTGGCTTTGCGCATGCGCTTCTGCTCCCCAG  10959

Query  309    CGATCTGAGGAGTGAACAGGACCCCACGGACGAGGATCCCTGCCGGGGTGTGGGCCCTGC  368
              ||||||||||||||||||||||||||||||||||||||||||||||||||||||||||||
Sbjct  10960  CGATCTGAGGAGTGAACAGGACCCCACGGACGAGGATCCCTGCCGGGGTGTGGGCCCTGC  11019

Query  369    TCTGATCACCACCCGCTGGCGCTCCCCCAGGGGCCGGAGCCGGGGCCGCCCCAGCACTGG  428
              ||||||||||||||||||||||||||||||||||||||||||||||||||||||||||||
Sbjct  11020  TCTGATCACCACCCGCTGGCGCTCCCCCAGGGGCCGGAGCCGGGGCCGCCCCAGCACTGG  11079

Query  429    GGGCGGGGTGGTTAGGGGCGGCCGTTGCGATGTATGTGGCAAGGTGTTCAGCCAACGCAG  488
              ||||||||||||||||||||||||||||||||||||||||||||||||||||||||||||
Sbjct  11080  GGGCGGGGTGGTTAGGGGCGGCCGTTGCGATGTATGTGGCAAGGTGTTCAGCCAACGCAG  11139

Query  489    CAACCTGCTGAGGCACCAGAAGATCCACACGGGTGAGCGACCATTCGTGTGCAGCGAGTG  548
              ||||||||||||||||||||||||||||||||||||||||||||||||||||||||||||
Sbjct  11140  CAACCTGCTGAGGCACCAGAAGATCCACACGGGTGAGCGACCATTCGTGTGCAGCGAGTG  11199

Query  549    CGGCCGCAGCTTCAGCCGCAGCTCGCACCTGCTGCGCCACCAGCTTACGCACACCGAGGA  608
              ||||||||||||||||||||||||||||||||||||||||||||||||||||||||||||
Sbjct  11200  CGGCCGCAGCTTCAGCCGCAGCTCGCACCTGCTGCGCCACCAGCTTACGCACACCGAGGA  11259

Query  609    GCGGCCGTTCGTGTGCGGCGACTGTGGCCAGGGCTTCGTGCGCAGCGCGCGCCTGGAAGA  668
              ||||||||||||||||||||||||||||||||||||||||||||||||||||||||||||
Sbjct  11260  GCGGCCGTTCGTGTGCGGCGACTGTGGCCAGGGCTTCGTGCGCAGCGCGCGCCTGGAAGA  11319

Query  669    GCATCGGAGAGTGCACACGGGCGAACAGCCTTTCCGTTGCGCTGAGTGCGGCCAGAGCTT  728
              ||||||||||||||||||||||||||||||||||||||||||||||||||||||||||||
Sbjct  11320  GCATCGGAGAGTGCACACGGGCGAACAGCCTTTCCGTTGCGCTGAGTGCGGCCAGAGCTT  11379

Query  729    CCGGCAGCGCTCCAATCTGCTGCAGCACCAGCGCATCCACGGCGATcccccgggccctgg  788
              ||||||||||||||||||||||||||||||||||||||||||||||||||||||||||||
Sbjct  11380  CCGGCAGCGCTCCAATCTGCTGCAGCACCAGCGCATCCACGGCGATCCCCCGGGCCCTGG  11439

Query  789    cgctaagcccccggcccctcctggtgcgcccgagcctcccggcccctTTCCGTGCAGCGA  848
              ||||||||||||||||||||||||||||||||||||||||||||||||||||||||||||
Sbjct  11440  CGCTAAGCCCCCGGCCCCTCCTGGTGCGCCCGAGCCTCCCGGCCCCTTTCCGTGCAGCGA  11499

Query  849    GTGCCGCGAGAGCTTCGCGCGGCGCGCCGTGC  880
              ||||||||||||||||||||||||||||||||
Sbjct  11500  GTGCCGCGAGAGCTTCGCGCGGCGCGCCGTGC  11531
```

Supplementary Figure S15: The down sequence (11437 to 12235) of MZF-1 with a FLAG-tag (amino acids 1-485) (GenBank Accession No. AF 161886, nucleotides 10781 to 12235). (**A**) The sequencing data was present using the CMV24 primer (TATTAGGACAAGGCTGGTGGGCAC) primer as initiator. (**B**) The “Query” (82 to 880) of sequence was aligned and identified by the NCBI BLAST.

A

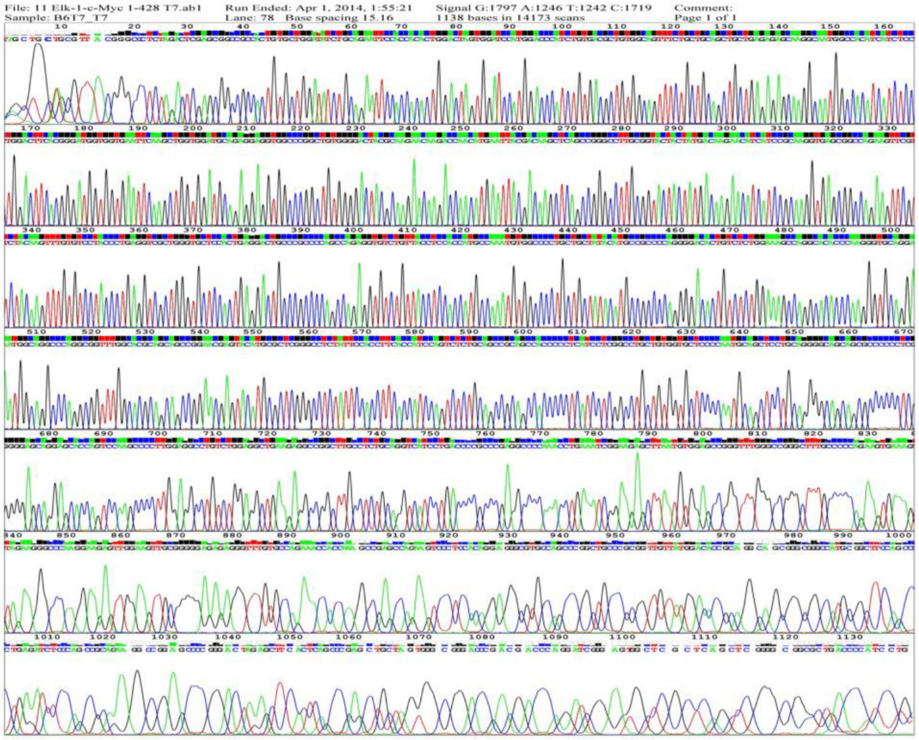

B

2014年6月30日 NCBI Blast:Nucleotide Sequence (800 letters)

Range 1: 101 to 810 GenBank Graphics Next Match Previous Match

Score	Expect	Identities	Gaps	Strand
1306 bits(707)	0.0	709/710(99%)	0/710(0%)	Plus/Plus

```
Query  91   ATGGACCCATCTGTGACGCTGTGGCAGTTTCTGCTGCAGCTGCTGAGAGAGCAAGGCAAT  150
            ||||||||||||||||||||||||||||||||||||||||||||||||||||||||||||
Sbjct  101  ATGGACCCATCTGTGACGCTGTGGCAGTTTCTGCTGCAGCTGCTGAGAGAGCAAGGCAAT  160

Query  151  GGCCACATCATCTCCTGGACTTCACGGGATGGTGGTGAATTCAAGCTGGTGGATGCAGAG  210
            ||||||||||||||||||||||||||||||||||||||||||||||||||||||||||||
Sbjct  161  GGCCACATCATCTCCTGGACTTCACGGGATGGTGGTGAATTCAAGCTGGTGGATGCAGAG  220

Query  211  GAGGTGGCCCGGCTGTGGGGACTACGCAAGAACAAGACCAACATGAATTACGACAAGCTC  270
            ||||||||||||||||||||||||||||||||||||||||||||||||||||||||||||
Sbjct  221  GAGGTGGCCCGGCTGTGGGGACTACGCAAGAACAAGACCAACATGAATTACGACAAGCTC  280

Query  271  AGCCGGGCCTTGCGGTACTACTATGACAAGAACATCATCCGCAAGGTGAGCGGCCAGAAG  330
            ||||||||||||||||||||||||||||||||||||||||||||||||||||||||||||
Sbjct  281  AGCCGGGCCTTGCGGTACTACTATGACAAGAACATCATCCGCAAGGTGAGCGGCCAGAAG  340

Query  331  TTCGTCTACAAGTTTGTGTCCTACCCTGAGGTCGCTGGGTGCTCCACTGAGGACTGCCCG  390
            ||||||||||||||||||||||||||||||||||| ||||||||||||||||||||||||
Sbjct  341  TTCGTCTACAAGTTTGTGTCCTACCCTGAGGTCGCAGGGTGCTCCACTGAGGACTGCCCG  400

Query  391  CCCCAGCCAGAGGTGTCTGTTACCTCCACCATGCCAAATGTGGCCCCTGCTGCTATACAT  450
            ||||||||||||||||||||||||||||||||||||||||||||||||||||||||||||
Sbjct  401  CCCCAGCCAGAGGTGTCTGTTACCTCCACCATGCCAAATGTGGCCCCTGCTGCTATACAT  460

Query  451  GCCGCCCCAGGGGACACTGTCTCTGGAAAGCCAGGCACACCCAAGGGTGCAGGAATGGCA  510
            ||||||||||||||||||||||||||||||||||||||||||||||||||||||||||||
Sbjct  461  GCCGCCCCAGGGGACACTGTCTCTGGAAAGCCAGGCACACCCAAGGGTGCAGGAATGGCA  520

Query  511  GGCCCAGGCGGTTTGGCACGCAGCAGCCGGAACGAGTACATGCGCTCGGGCCTCTATTCC  570
            ||||||||||||||||||||||||||||||||||||||||||||||||||||||||||||
Sbjct  521  GGCCCAGGCGGTTTGGCACGCAGCAGCCGGAACGAGTACATGCGCTCGGGCCTCTATTCC  580

Query  571  ACCTTCACCATCCAGTCTCTGCAGCCGCAGCCACCCCCTCATCCTCGGCCTGCTGTGGTG  630
            ||||||||||||||||||||||||||||||||||||||||||||||||||||||||||||
Sbjct  581  ACCTTCACCATCCAGTCTCTGCAGCCGCAGCCACCCCCTCATCCTCGGCCTGCTGTGGTG  640

Query  631  CTCCCCAATGCAGCTCCTGCAGGGGCAGCAGCGCCCCCCTCGGGGAGCAGGAGCACCAGT  690
            ||||||||||||||||||||||||||||||||||||||||||||||||||||||||||||
Sbjct  641  CTCCCCAATGCAGCTCCTGCAGGGGCAGCAGCGCCCCCCTCGGGGAGCAGGAGCACCAGT  700

Query  691  CCAAGCCCCTTGGAGGCCTGTCTGGAGGCTGAAGAGGCCGGCTTGCCTCTGCAGGTCATC  750
            ||||||||||||||||||||||||||||||||||||||||||||||||||||||||||||
Sbjct  701  CCAAGCCCCTTGGAGGCCTGTCTGGAGGCTGAAGAGGCCGGCTTGCCTCTGCAGGTCATC  760

Query  751  CTGACCCCGCCCGAGGCCCCAAACCTGAAATCGGAAGAGCTTAATGTGGA  800
            ||||||||||||||||||||||||||||||||||||||||||||||||||
Sbjct  761  CTGACCCCGCCCGAGGCCCCAAACCTGAAATCGGAAGAGCTTAATGTGGA  810
```

Supplementary Figure S16: The up sequence (101 to 810) of Elk-1 with a myc-tag (amino acids 1-428) (GenBank Accession No. AB016193, nucleotides 101 to 1384). (**A**) The sequencing data was present using the T7 primer as initiator. (**B**) The "Query" (91 to 800) of sequence was aligned and identified by the NCBI BLAST.

A

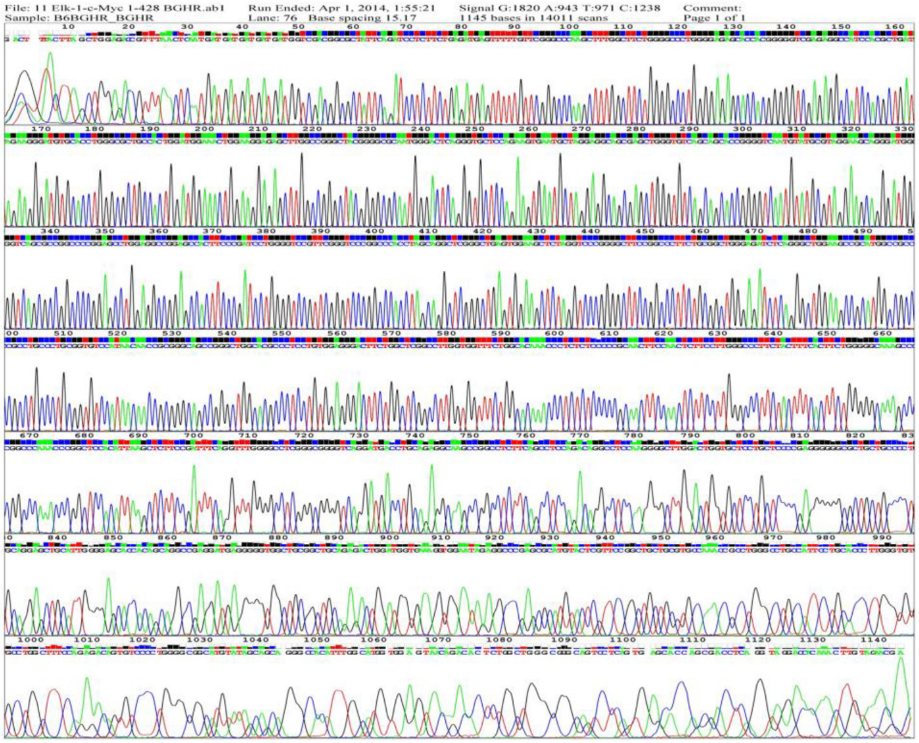

B

2014年6月30日 NCBI Blast:Nucleotide Sequence (800 letters)

Range 1: 691 to 1384 GenBank Graphics Next Match Previous Match

Score	Expect	Identities	Gaps	Strand
1282 bits(694)	0.0	694/694(100%)	0/694(0%)	Plus/Minus

```
Query  107   TGGCTTCTGGGGCCCTGGGGAGAGCACCACGGGGGTCGAGAGGCCATCCACGCTGATAGA  166
             ||||||||||||||||||||||||||||||||||||||||||||||||||||||||||||
Sbjct  1384  TGGCTTCTGGGGCCCTGGGGAGAGCACCACGGGGGTCGAGAGGCCATCCACGCTGATAGA  1325

Query  167   AGGGATGTGCACCTGGGCGCTGCCACTGGATGGAAACTGGAAGGAGAGCTTGGCCGGGCT  226
             ||||||||||||||||||||||||||||||||||||||||||||||||||||||||||||
Sbjct  1324  AGGGATGTGCACCTGGGCGCTGCCACTGGATGGAAACTGGAAGGAGAGCTTGGCCGGGCT  1265

Query  227   ACGGGGCGCAATGGGACTCAGGGTGCTCCAGAAGTGAATGCTAGGAGGCAGCGAGCTGGG  286
             ||||||||||||||||||||||||||||||||||||||||||||||||||||||||||||
Sbjct  1264  ACGGGGCGCAATGGGACTCAGGGTGCTCCAGAAGTGAATGCTAGGAGGCAGCGAGCTGGG  1205

Query  287   TGTCAGCAGCACCGGGGTCAATGTATGCGTAGGAAGCAGGGATGGGGTCAGCGCCGGCCC  346
             ||||||||||||||||||||||||||||||||||||||||||||||||||||||||||||
Sbjct  1204  TGTCAGCAGCACCGGGGTCAATGTATGCGTAGGAAGCAGGGATGGGGTCAGCGCCGGCCC  1145

Query  347   CGGAGCCTGGAGGCCGGAGCCACTTCCCGATCCTGGGGTCCGTTCGGGTCCCGGCCCACC  406
             ||||||||||||||||||||||||||||||||||||||||||||||||||||||||||||
Sbjct  1144  CGGAGCCTGGAGGCCGGAGCCACTTCCCGATCCTGGGGTCCGTTCGGGTCCCGGCCCACC  1085

Query  407   TAGCAGGCTCGGGCTGAGTGGAAGCTCTAGGTCCCGGGGCTTCCGGCCCTTCTGCGGCTG  466
             ||||||||||||||||||||||||||||||||||||||||||||||||||||||||||||
Sbjct  1084  TAGCAGGCTCGGGCTGAGTGGAAGCTCTAGGTCCCGGGGCTTCCGGCCCTTCTGCGGCTG  1025

Query  467   GGAGATCTCAGGGCTGGAAGCCGCATGGCCGCCCGCCTGCCCTGCGGTGTCCATAACAAC  526
             ||||||||||||||||||||||||||||||||||||||||||||||||||||||||||||
Sbjct  1024  GGAGATCTCAGGGCTGGAAGCCGCATGGCCGCCCGCCTGCCCTGCGGTGTCCATAACAAC  965

Query  527   CGCGGGCAGCCGGGCTGGCACGCCCTCCTGTGGAGGGACTTCTGGCTCGGCCTTGGTGGT  586
             ||||||||||||||||||||||||||||||||||||||||||||||||||||||||||||
Sbjct  964   CGCGGGCAGCCGGGCTGGCACGCCCTCCTGTGGAGGGACTTCTGGCTCGGCCTTGGTGGT  905

Query  587   TTCTGGCACAAACCCTCTCTCCCCCGCAACTTCCAACTCTTCCTTGGGCCCTTCTACTTT  646
             ||||||||||||||||||||||||||||||||||||||||||||||||||||||||||||
Sbjct  904   TTCTGGCACAAACCCTCTCTCCCCCGCAACTTCCAACTCTTCCTTGGGCCCTTCTACTTT  845

Query  647   CACTTCTGGGGGCAAAGCCCGGCCCAAACCCGGCTCCACATTAAGCTCTTCCGATTTCAG  706
             ||||||||||||||||||||||||||||||||||||||||||||||||||||||||||||
Sbjct  844   CACTTCTGGGGGCAAAGCCCGGCCCAAACCCGGCTCCACATTAAGCTCTTCCGATTTCAG  785

Query  707   GTTTGGGGCCTCGGGCGGGGTCAGGATGACCTGCAGAGGCAAGCCGGCCTCTTCAGCCTC  766
             ||||||||||||||||||||||||||||||||||||||||||||||||||||||||||||
Sbjct  784   GTTTGGGGCCTCGGGCGGGGTCAGGATGACCTGCAGAGGCAAGCCGGCCTCTTCAGCCTC  725

Query  767   CAGACAGGCCTCCAAGGGGCTTGGACTGGTGCTC  800
             ||||||||||||||||||||||||||||||||||
Sbjct  724   CAGACAGGCCTCCAAGGGGCTTGGACTGGTGCTC  691
```

Supplementary Figure S17: The down sequence (691 to 1384) of Elk-1 with a myc-tag (amino acids 1-428) (GenBank Accession No. AB016193, nucleotides 101 to 1384). (**A**) The sequencing data was present using the BGHR primer as initiator. (**B**) The "Query" (107 to 800) of sequence was aligned and identified by the NCBI BLAST. One nucleotide, "T", in the sequencing data for Elk-1 with a myc-tag differed from the nucleotide "A" found in the codon for the 376th aa in the GenBank AB016194 sequence. However, both "GCA" and "GCT" sequences encode the same amino acid (Ala).

A

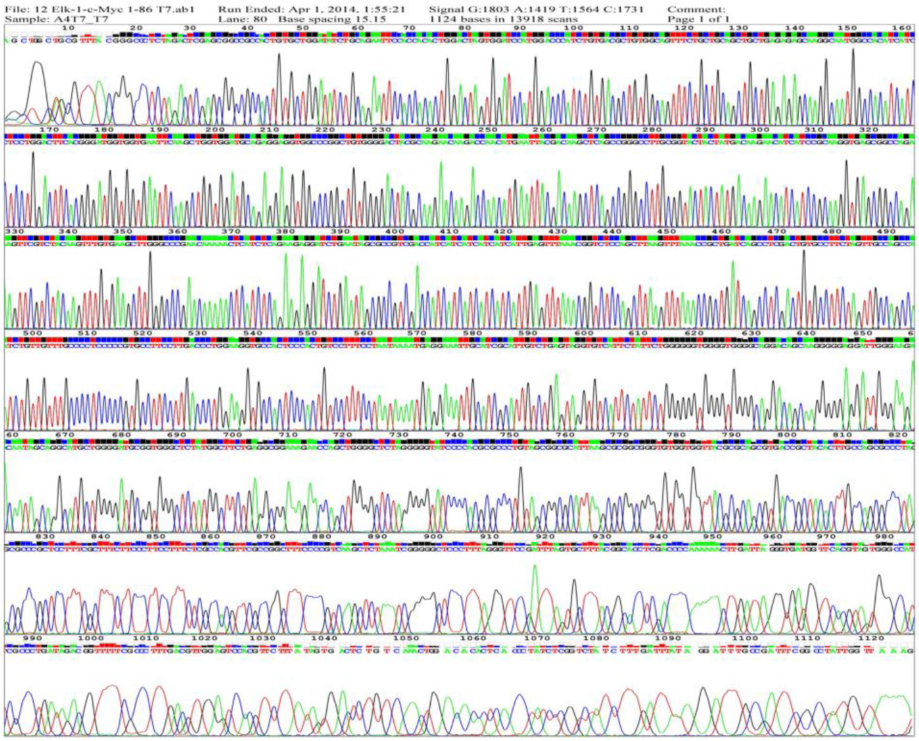

B

2014年6月30日 NCBI Blast:Nucleotide Sequence (400 letters)

Range 1: 101 to 358 GenBank Graphics Next Match Previous Match

Score	Expect	Identities	Gaps	Strand
477 bits(258)	4e-131	258/258(100%)	0/258(0%)	Plus/Plus

```
Query  91   ATGGACCCATCTGTGACGCTGTGGCAGTTTCTGCTGCAGCTGCTGAGAGAGCAAGGCAAT  150
            ||||||||||||||||||||||||||||||||||||||||||||||||||||||||||||
Sbjct  101  ATGGACCCATCTGTGACGCTGTGGCAGTTTCTGCTGCAGCTGCTGAGAGAGCAAGGCAAT  160

Query  151  GGCCACATCATCTCCTGGACTTCACGGGATGGTGGTGAATTCAAGCTGGTGGATGCAGAG  210
            ||||||||||||||||||||||||||||||||||||||||||||||||||||||||||||
Sbjct  161  GGCCACATCATCTCCTGGACTTCACGGGATGGTGGTGAATTCAAGCTGGTGGATGCAGAG  220

Query  211  GAGGTGGCCCGGCTGTGGGGACTACGCAAGAACAAGACCAACATGAATTACGACAAGCTC  270
            ||||||||||||||||||||||||||||||||||||||||||||||||||||||||||||
Sbjct  221  GAGGTGGCCCGGCTGTGGGGACTACGCAAGAACAAGACCAACATGAATTACGACAAGCTC  280

Query  271  AGCCGGGCCTTGCGGTACTACTATGACAAGAACATCATCCGCAAGGTGAGCGGCCAGAAG  330
            ||||||||||||||||||||||||||||||||||||||||||||||||||||||||||||
Sbjct  281  AGCCGGGCCTTGCGGTACTACTATGACAAGAACATCATCCGCAAGGTGAGCGGCCAGAAG  340

Query  331  TTCGTCTACAAGTTTGTG  348
            ||||||||||||||||||
Sbjct  341  TTCGTCTACAAGTTTGTG  358
```

Supplementary Figure S18: Elk-1 with a myc-tag (amino acids 1-86) (GenBank Accession No. AB016193, nucleotides 101 to 358). (**A**) The sequencing data was present using the T7 primer as initiator. (**B**) The "Query" (93 to 350) of sequence was aligned and identified by the NCBI BLAST.

A

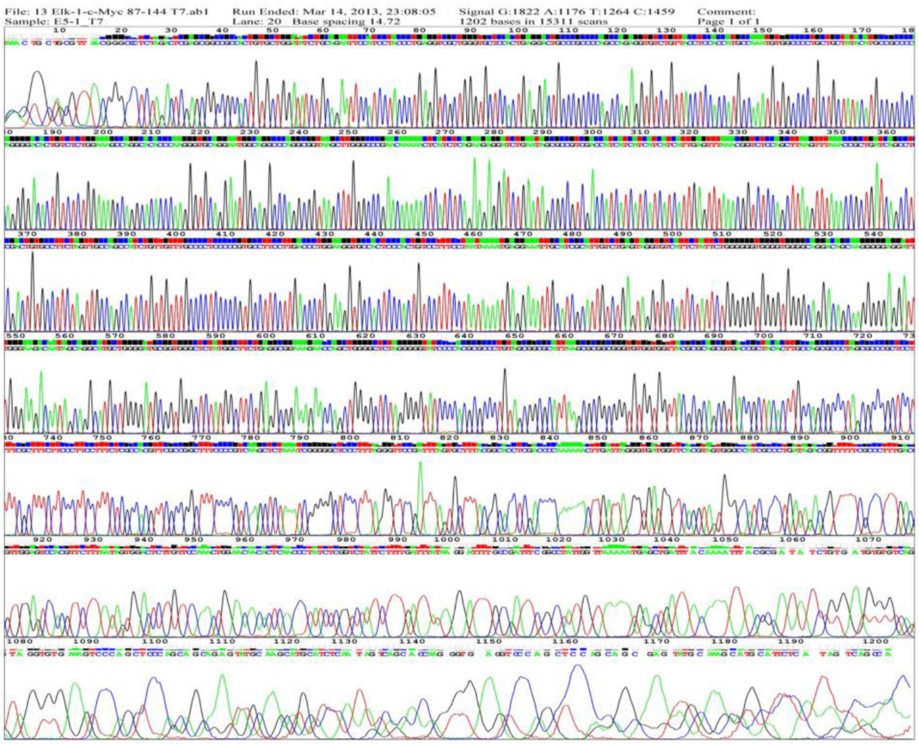

B

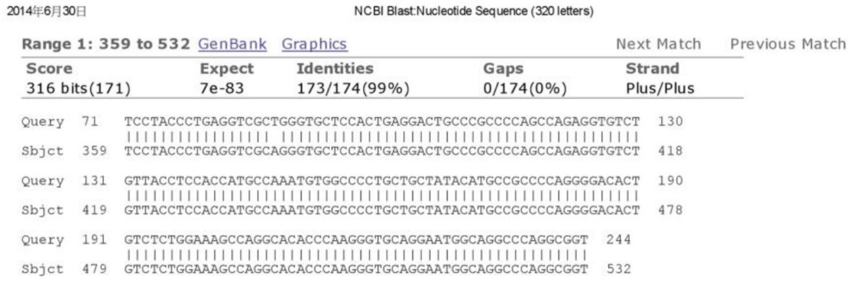

2014年6月30日 NCBI Blast:Nucleotide Sequence (320 letters)

Range 1: 359 to 532 GenBank Graphics Next Match Previous Match

Score	Expect	Identities	Gaps	Strand
316 bits(171)	7e-83	173/174(99%)	0/174(0%)	Plus/Plus

```
Query  71   TCCTACCCTGAGGTCGCTGGGTGCTCCACTGAGGACTGCCCGCCCCAGCCAGAGGTGTCT  130
            ||||||||||||||||| ||||||||||||||||||||||||||||||||||||||||||
Sbjct  359  TCCTACCCTGAGGTCGCAGGGTGCTCCACTGAGGACTGCCCGCCCCAGCCAGAGGTGTCT  418

Query  131  GTTACCTCCACCATGCCAAATGTGGCCCCTGCTGCTATACATGCCGCCCCAGGGGACACT  190
            ||||||||||||||||||||||||||||||||||||||||||||||||||||||||||||
Sbjct  419  GTTACCTCCACCATGCCAAATGTGGCCCCTGCTGCTATACATGCCGCCCCAGGGGACACT  478

Query  191  GTCTCTGGAAAGCCAGGCACACCCAAGGGTGCAGGAATGGCAGGCCCAGGCGGT  244
            ||||||||||||||||||||||||||||||||||||||||||||||||||||||
Sbjct  479  GTCTCTGGAAAGCCAGGCACACCCAAGGGTGCAGGAATGGCAGGCCCAGGCGGT  532
```

Supplementary Figure S19: Elk-1 with a myc-tag (amino acids 87-144) (GenBank Accession No. AB016193, nucleotides 359 to 532). (**A**) The sequencing data was present using the T7 primer as initiator. (**B**) The "Query" (71 to 244) of sequence was aligned and identified by the NCBI BLAST.

A

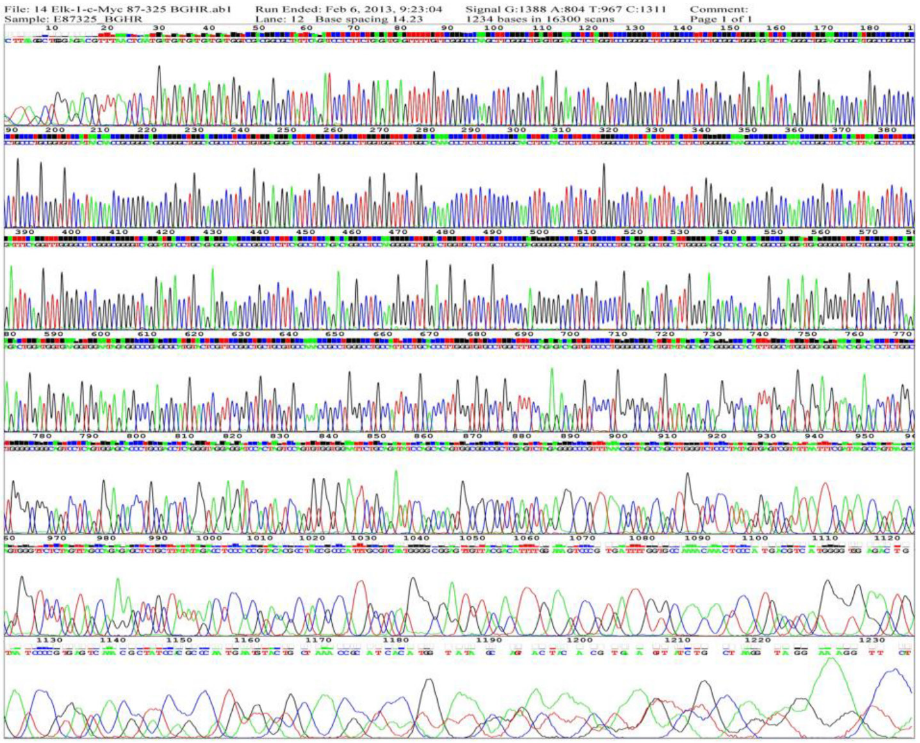

B

2014年6月30日 NCBI Blast:Nucleotide Sequence (880 letters)

Range 1: 359 to 1076 GenBank Graphics Next Match Previous Match

Score	Expect	Identities	Gaps	Strand
1327 bits(718)	0.0	718/718(100%)	0/718(0%)	Plus/Minus

```
Query  102   TCGGGCTGAGTGGAAGCTCTAGGTCCCGGGGCTTCCGGCCCTTCTGCGGCTGGGAGATCT  161
             ||||||||||||||||||||||||||||||||||||||||||||||||||||||||||||
Sbjct  1076  TCGGGCTGAGTGGAAGCTCTAGGTCCCGGGGCTTCCGGCCCTTCTGCGGCTGGGAGATCT  1017

Query  162   CAGGGCTGGAAGCCGCATGGCCGCCCGCCTGCCCTGCGGTGTCCATAACAACCGCGGGCA  221
             ||||||||||||||||||||||||||||||||||||||||||||||||||||||||||||
Sbjct  1016  CAGGGCTGGAAGCCGCATGGCCGCCCGCCTGCCCTGCGGTGTCCATAACAACCGCGGGCA  957

Query  222   GCCGGGCTGGCACGCCCTCCTGTGGAGGGACTTCTGGCTCGGCCTTGGTGGTTTCTGGCA  281
             ||||||||||||||||||||||||||||||||||||||||||||||||||||||||||||
Sbjct  956   GCCGGGCTGGCACGCCCTCCTGTGGAGGGACTTCTGGCTCGGCCTTGGTGGTTTCTGGCA  897

Query  282   CAAACCCTCTCTCCCCCGCAACTTCCAACTCTTCCTTGGGCCCTTCTACTTTCACTTCTG  341
             ||||||||||||||||||||||||||||||||||||||||||||||||||||||||||||
Sbjct  896   CAAACCCTCTCTCCCCCGCAACTTCCAACTCTTCCTTGGGCCCTTCTACTTTCACTTCTG  837

Query  342   GGGGCAAAGCCCGGCCCAAACCCGGCTCCACATTAAGCTCTTCCGATTTCAGGTTTGGGG  401
             ||||||||||||||||||||||||||||||||||||||||||||||||||||||||||||
Sbjct  836   GGGGCAAAGCCCGGCCCAAACCCGGCTCCACATTAAGCTCTTCCGATTTCAGGTTTGGGG  777

Query  402   CCTCGGGCGGGGTCAGGATGACCTGCAGAGGCAAGCCGGCCTCTTCAGCCTCCAGACAGG  461
             ||||||||||||||||||||||||||||||||||||||||||||||||||||||||||||
Sbjct  776   CCTCGGGCGGGGTCAGGATGACCTGCAGAGGCAAGCCGGCCTCTTCAGCCTCCAGACAGG  717

Query  462   CCTCCAAGGGGCTTGGACTGGTGCTCCTGCTCCCCGAGGGGGGCGCTGCTGCCCCTGCAG  521
             ||||||||||||||||||||||||||||||||||||||||||||||||||||||||||||
Sbjct  716   CCTCCAAGGGGCTTGGACTGGTGCTCCTGCTCCCCGAGGGGGGCGCTGCTGCCCCTGCAG  657

Query  522   GAGCTGCATTGGGGAGCACCACAGCAGGCCGAGGATGAGGGGGTGGCTGCGGCTGCAGAG  581
             ||||||||||||||||||||||||||||||||||||||||||||||||||||||||||||
Sbjct  656   GAGCTGCATTGGGGAGCACCACAGCAGGCCGAGGATGAGGGGGTGGCTGCGGCTGCAGAG  597

Query  582   ACTGGATGGTGAAGGTGGAATAGAGGCCCGAGCGCATGTACTCGTTCCGGCTGCTGCGTG  641
             ||||||||||||||||||||||||||||||||||||||||||||||||||||||||||||
Sbjct  596   ACTGGATGGTGAAGGTGGAATAGAGGCCCGAGCGCATGTACTCGTTCCGGCTGCTGCGTG  537

Query  642   CCAAACCGCCTGGGCCTGCCATTCCTGCACCCTTGGGTGTGCCTGGCTTTCCAGAGACAG  701
             ||||||||||||||||||||||||||||||||||||||||||||||||||||||||||||
Sbjct  536   CCAAACCGCCTGGGCCTGCCATTCCTGCACCCTTGGGTGTGCCTGGCTTTCCAGAGACAG  477

Query  702   TGTCCCCTGGGGCGGCATGTATAGCAGCAGGGGCCACATTTGGCATGGTGGAGGTAACAG  761
             ||||||||||||||||||||||||||||||||||||||||||||||||||||||||||||
Sbjct  476   TGTCCCCTGGGGCGGCATGTATAGCAGCAGGGGCCACATTTGGCATGGTGGAGGTAACAG  417

Query  762   ACACCTCTGGCTGGGGCGGGCAGTCCTCAGTGGAGCACCCTGCGACCTCAGGGTAGGA  819
             ||||||||||||||||||||||||||||||||||||||||||||||||||||||||||
Sbjct  416   ACACCTCTGGCTGGGGCGGGCAGTCCTCAGTGGAGCACCCTGCGACCTCAGGGTAGGA  359
```

Supplementary Figure S20: Elk-1 with a myc-tag (amino acids 87-325) (GenBank Accession No. AB016193, nucleotides 359 to 1075). (**A**) The sequencing data was present using the BGHR primer as initiator. (**B**) The "Query" (103 to 800) of sequence was aligned and identified by the NCBI BLAST.

A

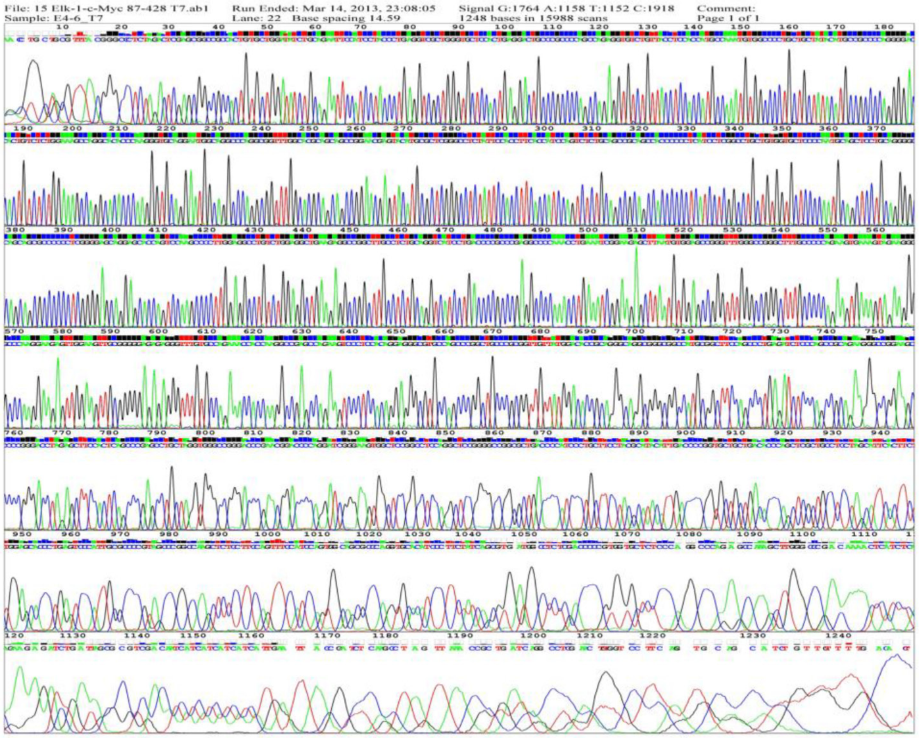

B

2014年6月30日 NCBI Blast:Nucleotide Sequence (880 letters)

Range 1: 359 to 1168 GenBank Graphics Next Match Previous Match

Score	Expect	Identities	Gaps	Strand
1485 bits(804)	0.0	809/811(99%)	1/811(0%)	Plus/Plus

```
Query  70    TCCTACCCTGAGGTCGCTGGGTGCTCCACTGAGGACTGCCCGCCCCAGCCAGAGGTGTCT  129
             |||||||||||||||||| |||||||||||||||||||||||||||||||||||||||||
Sbjct  359   TCCTACCCTGAGGTCGCAGGGTGCTCCACTGAGGACTGCCCGCCCCAGCCAGAGGTGTCT  418

Query  130   GTTACCTCCACCATGCCAAATGTGGCCCCTGCTGCTATACATGCCGCCCCAGGGGACACT  189
             ||||||||||||||||||||||||||||||||||||||||||||||||||||||||||||
Sbjct  419   GTTACCTCCACCATGCCAAATGTGGCCCCTGCTGCTATACATGCCGCCCCAGGGGACACT  478

Query  190   GTCTCTGGAAAGCCAGGCACACCCAAGGGTGCAGGAATGGCAGGCCCAGGCGGTTTGGCA  249
             ||||||||||||||||||||||||||||||||||||||||||||||||||||||||||||
Sbjct  479   GTCTCTGGAAAGCCAGGCACACCCAAGGGTGCAGGAATGGCAGGCCCAGGCGGTTTGGCA  538

Query  250   CGCAGCAGCCGGAACGAGTACATGCGCTCGGGCCTCTATTCCACCTTCACCATCCAGTCT  309
             ||||||||||||||||||||||||||||||||||||||||||||||||||||||||||||
Sbjct  539   CGCAGCAGCCGGAACGAGTACATGCGCTCGGGCCTCTATTCCACCTTCACCATCCAGTCT  598

Query  310   CTGCAGCCGCAGCCACCCCCCTCATCCTCGGCCTGCTGTGGTGCTCCCCAATGCAGCTCC  369
             |||||||||||||| |||||||||||||||||||||||||||||||||||||||||||||
Sbjct  599   CTGCAGCCGCAGCCA-CCCCCTCATCCTCGGCCTGCTGTGGTGCTCCCCAATGCAGCTCC  657

Query  370   TGCAGGGGCAGCAGCGCCCCCCTCGGGGAGCAGGAGCACCAGTCCAAGCCCCTTGGAGGC  429
             ||||||||||||||||||||||||||||||||||||||||||||||||||||||||||||
Sbjct  658   TGCAGGGGCAGCAGCGCCCCCCTCGGGGAGCAGGAGCACCAGTCCAAGCCCCTTGGAGGC  717

Query  430   CTGTCTGGAGGCTGAAGAGGCCGGCTTGCCTCTGCAGGTCATCCTGACCCCGCCCGAGGC  489
             ||||||||||||||||||||||||||||||||||||||||||||||||||||||||||||
Sbjct  718   CTGTCTGGAGGCTGAAGAGGCCGGCTTGCCTCTGCAGGTCATCCTGACCCCGCCCGAGGC  777

Query  490   CCCAAACCTGAAATCGGAAGAGCTTAATGTGGAGCCGGGTTTGGGCCGGGCTTTGCCCCC  549
             ||||||||||||||||||||||||||||||||||||||||||||||||||||||||||||
Sbjct  778   CCCAAACCTGAAATCGGAAGAGCTTAATGTGGAGCCGGGTTTGGGCCGGGCTTTGCCCCC  837

Query  550   AGAAGTGAAAGTAGAAGGGCCCAAGGAAGAGTTGGAAGTTGCGGGGGAGAGAGGGTTTGT  609
             ||||||||||||||||||||||||||||||||||||||||||||||||||||||||||||
Sbjct  838   AGAAGTGAAAGTAGAAGGGCCCAAGGAAGAGTTGGAAGTTGCGGGGGAGAGAGGGTTTGT  897

Query  610   GCCAGAAACCACCAAGGCCGAGCCAGAAGTCCCTCCACAGGAGGGCGTGCCAGCCCGGCT  669
             ||||||||||||||||||||||||||||||||||||||||||||||||||||||||||||
Sbjct  898   GCCAGAAACCACCAAGGCCGAGCCAGAAGTCCCTCCACAGGAGGGCGTGCCAGCCCGGCT  957

Query  670   GCCCGCGGTTGTTATGGACACCGCAGGGCAGGCGGGCGGCCATGCGGCTTCCAGCCCTGA  729
             ||||||||||||||||||||||||||||||||||||||||||||||||||||||||||||
Sbjct  958   GCCCGCGGTTGTTATGGACACCGCAGGGCAGGCGGGCGGCCATGCGGCTTCCAGCCCTGA  1017

Query  730   GATCTCCCAGCCGCAGAAGGGCCGGAAGCCCCGGGACCTAGAGCTTCCACTCAGCCCGAG  789
             ||||||||||||||||||||||||||||||||||||||||||||||||||||||||||||
Sbjct  1018  GATCTCCCAGCCGCAGAAGGGCCGGAAGCCCCGGGACCTAGAGCTTCCACTCAGCCCGAG  1077

Query  790   CCTGCTAGGTGGGCCGGGACCCGAACGGACCCCAGGATCGGGAAGTGGCTCCGGCCTCCA  849
             ||||||||||||||||||||||||||||||||||||||||||||||||||||||||||||
Sbjct  1078  CCTGCTAGGTGGGCCGGGACCCGAACGGACCCCAGGATCGGGAAGTGGCTCCGGCCTCCA  1137

Query  850   GGCTCCGGGGCCGGCGCTGACCCCATCCCTG  880
             |||||||||||||||||||||||||||||||
Sbjct  1138  GGCTCCGGGGCCGGCGCTGACCCCATCCCTG  1168
```

Supplementary Figure S21: The up sequence (359 to 1186) of Elk-1 with a myc-tag (amino acids 87-428) (GenBank Accession No. AB016193, nucleotides 359 to 1384). (**A**) The sequencing data was present using the T7 primer as initiator. (**B**) The "Query" (70 to 897) of sequence was aligned and identified by the NCBI BLAST.

A

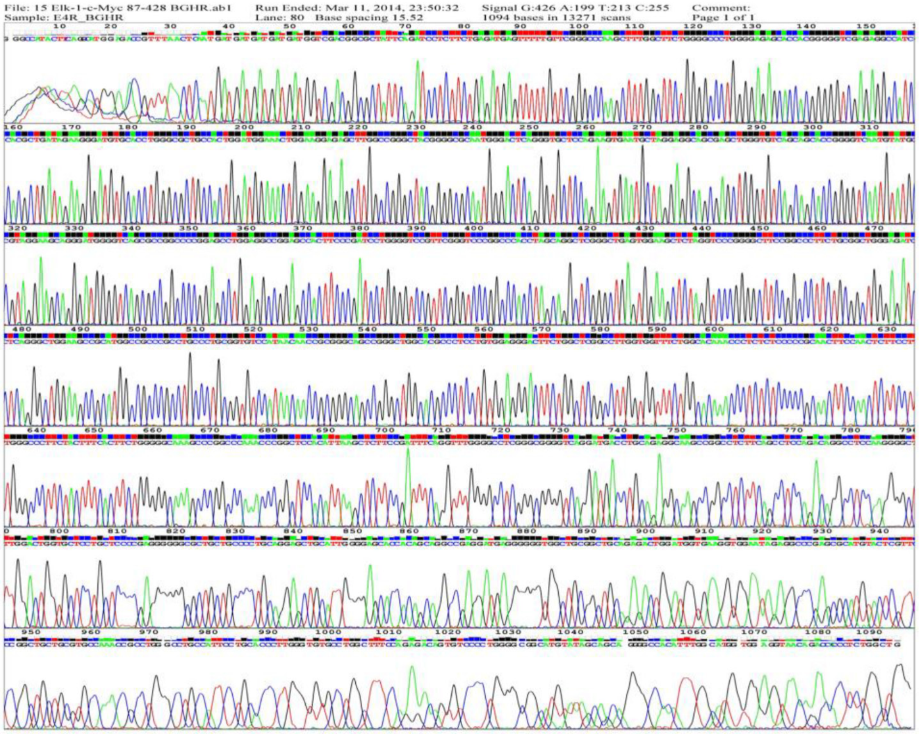

B

2014年6月30日 NCBI Blast:Nucleotide Sequence (880 letters)

```
Range 1: 615 to 1384 GenBank  Graphics                       Next Match   Previous Match
Score            Expect   Identities         Gaps         Strand
1423 bits(770)   0.0      770/770(100%)      0/770(0%)    Plus/Minus

Query  111   TGGCTTCTGGGGCCCTGGGGAGAGCACCACGGGGGTCGAGAGGCCATCCACGCTGATAGA  170
             ||||||||||||||||||||||||||||||||||||||||||||||||||||||||||||
Sbjct  1384  TGGCTTCTGGGGCCCTGGGGAGAGCACCACGGGGGTCGAGAGGCCATCCACGCTGATAGA  1325

Query  171   AGGGATGTGCACCTGGGCGCTGCCACTGGATGGAAACTGGAAGGAGAGCTTGGCCGGGCT  230
             ||||||||||||||||||||||||||||||||||||||||||||||||||||||||||||
Sbjct  1324  AGGGATGTGCACCTGGGCGCTGCCACTGGATGGAAACTGGAAGGAGAGCTTGGCCGGGCT  1265

Query  231   ACGGGGCGCAATGGGACTCAGGGTGCTCCAGAAGTGAATGCTAGGAGGCAGCGAGCTGGG  290
             ||||||||||||||||||||||||||||||||||||||||||||||||||||||||||||
Sbjct  1264  ACGGGGCGCAATGGGACTCAGGGTGCTCCAGAAGTGAATGCTAGGAGGCAGCGAGCTGGG  1205

Query  291   TGTCAGCAGCACCGGGGTCAATGTATGCGTAGGAAGCAGGGATGGGGTCAGCGCCGGCCC  350
             ||||||||||||||||||||||||||||||||||||||||||||||||||||||||||||
Sbjct  1204  TGTCAGCAGCACCGGGGTCAATGTATGCGTAGGAAGCAGGGATGGGGTCAGCGCCGGCCC  1145

Query  351   CGGAGCCTGGAGGCCGGAGCCACTTCCCGATCCTGGGGTCCGTTCGGGTCCCGGCCCACC  410
             ||||||||||||||||||||||||||||||||||||||||||||||||||||||||||||
Sbjct  1144  CGGAGCCTGGAGGCCGGAGCCACTTCCCGATCCTGGGGTCCGTTCGGGTCCCGGCCCACC  1085

Query  411   TAGCAGGCTCGGGCTGAGTGGAAGCTCTAGGTCCCGGGGCTTCCGGCCCTTCTGCGGCTG  470
             ||||||||||||||||||||||||||||||||||||||||||||||||||||||||||||
Sbjct  1084  TAGCAGGCTCGGGCTGAGTGGAAGCTCTAGGTCCCGGGGCTTCCGGCCCTTCTGCGGCTG  1025

Query  471   GGAGATCTCAGGGCTGGAAGCCGCATGGCCGCCCGCCTGCCCTGCGGTGTCCATAACAAC  530
             ||||||||||||||||||||||||||||||||||||||||||||||||||||||||||||
Sbjct  1024  GGAGATCTCAGGGCTGGAAGCCGCATGGCCGCCCGCCTGCCCTGCGGTGTCCATAACAAC  965

Query  531   CGCGGGCAGCCGGGCTGGCACGCCCTCCTGTGGAGGGACTTCTGGCTCGGCCTTGGTGGT  590
             ||||||||||||||||||||||||||||||||||||||||||||||||||||||||||||
Sbjct  964   CGCGGGCAGCCGGGCTGGCACGCCCTCCTGTGGAGGGACTTCTGGCTCGGCCTTGGTGGT  905

Query  591   TTCTGGCACAAACCCTCTCTCCCCCGCAACTTCCAACTCTTCCTTGGGCCCTTCTACTTT  650
             ||||||||||||||||||||||||||||||||||||||||||||||||||||||||||||
Sbjct  904   TTCTGGCACAAACCCTCTCTCCCCCGCAACTTCCAACTCTTCCTTGGGCCCTTCTACTTT  845

Query  651   CACTTCTGGGGGCAAAGCCCGGCCCAAACCCGGCTCCACATTAAGCTCTTCCGATTTCAG  710
             ||||||||||||||||||||||||||||||||||||||||||||||||||||||||||||
Sbjct  844   CACTTCTGGGGGCAAAGCCCGGCCCAAACCCGGCTCCACATTAAGCTCTTCCGATTTCAG  785

Query  711   GTTTGGGGCCTCGGGCGGGGTCAGGATGACCTGCAGAGGCAAGCCGGCCTCTTCAGCCTC  770
             ||||||||||||||||||||||||||||||||||||||||||||||||||||||||||||
Sbjct  784   GTTTGGGGCCTCGGGCGGGGTCAGGATGACCTGCAGAGGCAAGCCGGCCTCTTCAGCCTC  725

Query  771   CAGACAGGCCTCCAAGGGGCTTGGACTGGTGCTCCTGCTCCCCGAGGGGGGCGCTGCTGC  830
             ||||||||||||||||||||||||||||||||||||||||||||||||||||||||||||
Sbjct  724   CAGACAGGCCTCCAAGGGGCTTGGACTGGTGCTCCTGCTCCCCGAGGGGGGCGCTGCTGC  665

Query  831   CCCTGCAGGAGCTGCATTGGGGAGCACCACAGCAGGCCGAGGATGAGGGG  880
             ||||||||||||||||||||||||||||||||||||||||||||||||||
Sbjct  664   CCCTGCAGGAGCTGCATTGGGGAGCACCACAGCAGGCCGAGGATGAGGGG  615
```

Supplementary Figure S22: The down sequence (695 to 1384) of Elk-1 with a myc-tag (amino acids 87-428) (GenBank Accession No. AB016193, nucleotides 359 to 1384). (**A**) The sequencing data was present using the BGHR primer as initiator. (**B**) The "Query" (111 to 880) of sequence was aligned and identified by the NCBI BLAST.

A

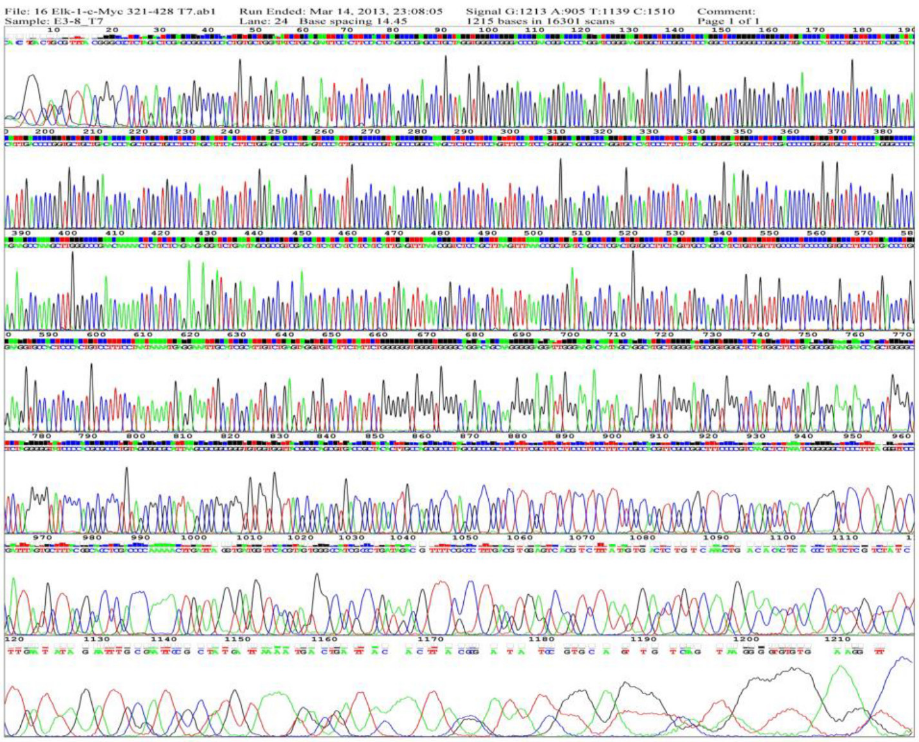

B

2014年6月30日 NCBI Blast:Nucleotide Sequence (400 letters)

Range 1: 1061 to 1384 GenBank Graphics Next Match Previous Match

Score	Expect	Identities	Gaps	Strand
599 bits(324)	8e-168	324/324(100%)	0/324(0%)	Plus/Plus

```
Query  71    CTTCCACTCAGCCCGAGCCTGCTAGGTGGGCCGGGACCCGAACGGACCCCAGGATCGGGA  130
             ||||||||||||||||||||||||||||||||||||||||||||||||||||||||||||
Sbjct  1061  CTTCCACTCAGCCCGAGCCTGCTAGGTGGGCCGGGACCCGAACGGACCCCAGGATCGGGA  1120

Query  131   AGTGGCTCCGGCCTCCAGGCTCCGGGGCCGGCGCTGACCCCATCCCTGCTTCCTACGCAT  190
             ||||||||||||||||||||||||||||||||||||||||||||||||||||||||||||
Sbjct  1121  AGTGGCTCCGGCCTCCAGGCTCCGGGGCCGGCGCTGACCCCATCCCTGCTTCCTACGCAT  1180

Query  191   ACATTGACCCCGGTGCTGCTGACACCCAGCTCGCTGCCTCCTAGCATTCACTTCTGGAGC  250
             ||||||||||||||||||||||||||||||||||||||||||||||||||||||||||||
Sbjct  1181  ACATTGACCCCGGTGCTGCTGACACCCAGCTCGCTGCCTCCTAGCATTCACTTCTGGAGC  1240

Query  251   ACCCTGAGTCCCATTGCGCCCCGTAGCCCGGCCAAGCTCTCCTTCCAGTTTCCATCCAGT  310
             ||||||||||||||||||||||||||||||||||||||||||||||||||||||||||||
Sbjct  1241  ACCCTGAGTCCCATTGCGCCCCGTAGCCCGGCCAAGCTCTCCTTCCAGTTTCCATCCAGT  1300

Query  311   GGCAGCGCCCAGGTGCACATCCCTTCTATCAGCGTGGATGGCCTCTCGACCCCCGTGGTG  370
             ||||||||||||||||||||||||||||||||||||||||||||||||||||||||||||
Sbjct  1301  GGCAGCGCCCAGGTGCACATCCCTTCTATCAGCGTGGATGGCCTCTCGACCCCCGTGGTG  1360

Query  371   CTCTCCCCAGGGCCCCAGAAGCCA  394
             ||||||||||||||||||||||||
Sbjct  1361  CTCTCCCCAGGGCCCCAGAAGCCA  1384
```

Supplementary Figure S23: Elk-1 with a myc-tag (amino acids 321-428) (GenBank Accession No. AB016193, nucleotides 1061 to 1384). (**A**) The sequencing data was present using the T7 primer as initiator. (**B**) The “Query” (71 to 394) of sequence was aligned and identified by the NCBI BLAST.

A

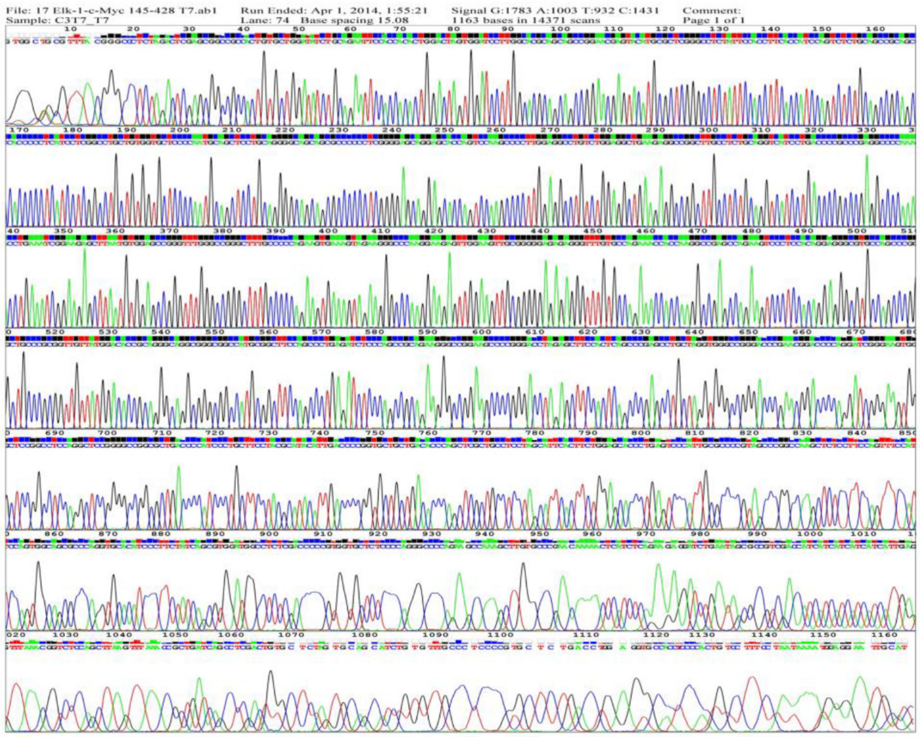

B

2014年6月30日 NCBI Blast:Nucleotide Sequence (800 letters)

Range 1: 533 to 1244 GenBank Graphics Next Match Previous Match

Score	Expect	Identities	Gaps	Strand
1315 bits(712)	0.0	712/712(100%)	0/712(0%)	Plus/Plus

```
Query  89    TTGGCACGCAGCAGCCGGAACGAGTACATGCGCTCGGGCCTCTATTCCACCTTCACCATC  148
             ||||||||||||||||||||||||||||||||||||||||||||||||||||||||||||
Sbjct  533   TTGGCACGCAGCAGCCGGAACGAGTACATGCGCTCGGGCCTCTATTCCACCTTCACCATC  592

Query  149   CAGTCTCTGCAGCCGCAGCCACCCCCTCATCCTCGGCCTGCTGTGGTGCTCCCCAATGCA  208
             ||||||||||||||||||||||||||||||||||||||||||||||||||||||||||||
Sbjct  593   CAGTCTCTGCAGCCGCAGCCACCCCCTCATCCTCGGCCTGCTGTGGTGCTCCCCAATGCA  652

Query  209   GCTCCTGCAGGGGCAGCAGCGCCCCCCTCGGGGAGCAGGAGCACCAGTCCAAGCCCCTTG  268
             ||||||||||||||||||||||||||||||||||||||||||||||||||||||||||||
Sbjct  653   GCTCCTGCAGGGGCAGCAGCGCCCCCCTCGGGGAGCAGGAGCACCAGTCCAAGCCCCTTG  712

Query  269   GAGGCCTGTCTGGAGGCTGAAGAGGCCGGCTTGCCTCTGCAGGTCATCCTGACCCCGCCC  328
             ||||||||||||||||||||||||||||||||||||||||||||||||||||||||||||
Sbjct  713   GAGGCCTGTCTGGAGGCTGAAGAGGCCGGCTTGCCTCTGCAGGTCATCCTGACCCCGCCC  772

Query  329   GAGGCCCCAAACCTGAAATCGGAAGAGCTTAATGTGGAGCCGGGTTTGGGCCGGGCTTTG  388
             ||||||||||||||||||||||||||||||||||||||||||||||||||||||||||||
Sbjct  773   GAGGCCCCAAACCTGAAATCGGAAGAGCTTAATGTGGAGCCGGGTTTGGGCCGGGCTTTG  832

Query  389   CCCCCAGAAGTGAAAGTAGAAGGGCCCAAGGAAGAGTTGGAAGTTGCGGGGGAGAGAGGG  448
             ||||||||||||||||||||||||||||||||||||||||||||||||||||||||||||
Sbjct  833   CCCCCAGAAGTGAAAGTAGAAGGGCCCAAGGAAGAGTTGGAAGTTGCGGGGGAGAGAGGG  892

Query  449   TTTGTGCCAGAAACCACCAAGGCCGAGCCAGAAGTCCCTCCACAGGAGGGCGTGCCAGCC  508
             ||||||||||||||||||||||||||||||||||||||||||||||||||||||||||||
Sbjct  893   TTTGTGCCAGAAACCACCAAGGCCGAGCCAGAAGTCCCTCCACAGGAGGGCGTGCCAGCC  952

Query  509   CGGCTGCCCGCGGTTGTTATGGACACCGCAGGGCAGGCGGGCGGCCATGCGGCTTCCAGC  568
             ||||||||||||||||||||||||||||||||||||||||||||||||||||||||||||
Sbjct  953   CGGCTGCCCGCGGTTGTTATGGACACCGCAGGGCAGGCGGGCGGCCATGCGGCTTCCAGC  1012

Query  569   CCTGAGATCTCCCAGCCGCAGAAGGGCCGGAAGCCCCGGGACCTAGAGCTTCCACTCAGC  628
             ||||||||||||||||||||||||||||||||||||||||||||||||||||||||||||
Sbjct  1013  CCTGAGATCTCCCAGCCGCAGAAGGGCCGGAAGCCCCGGGACCTAGAGCTTCCACTCAGC  1072

Query  629   CCGAGCCTGCTAGGTGGGCCGGGACCCGAACGGACCCCAGGATCGGGAAGTGGCTCCGGC  688
             ||||||||||||||||||||||||||||||||||||||||||||||||||||||||||||
Sbjct  1073  CCGAGCCTGCTAGGTGGGCCGGGACCCGAACGGACCCCAGGATCGGGAAGTGGCTCCGGC  1132

Query  689   CTCCAGGCTCCGGGGCCGGCGCTGACCCCATCCCTGCTTCCTACGCATACATTGACCCCG  748
             ||||||||||||||||||||||||||||||||||||||||||||||||||||||||||||
Sbjct  1133  CTCCAGGCTCCGGGGCCGGCGCTGACCCCATCCCTGCTTCCTACGCATACATTGACCCCG  1192

Query  749   GTGCTGCTGACACCCAGCTCGCTGCCTCCTAGCATTCACTTCTGGAGCACCC  800
             ||||||||||||||||||||||||||||||||||||||||||||||||||||
Sbjct  1193  GTGCTGCTGACACCCAGCTCGCTGCCTCCTAGCATTCACTTCTGGAGCACCC  1244
```

Supplementary Figure S24: The up sequence (533 to 1244) of Elk-1 with a myc-tag (amino acids 145-428) (GenBank Accession No. AB016193, nucleotides 533 to 1384). (**A**) The sequencing data was present using the T7 primer as initiator. (**B**) The "Query" (89 to 800) of sequence was aligned and identified by the NCBI BLAST.

A

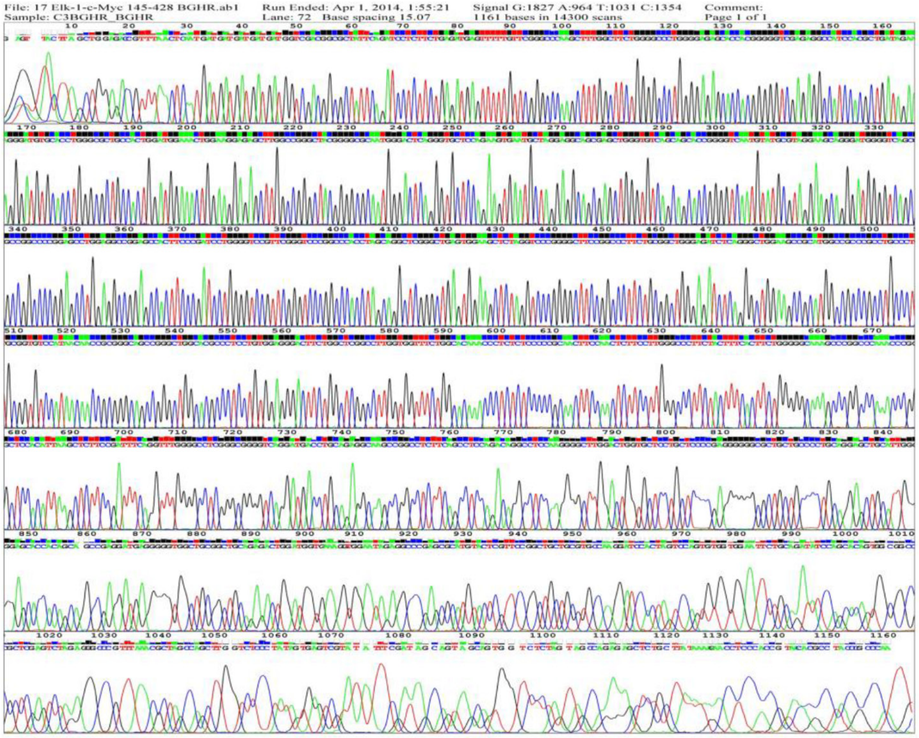

B

2014年6月30日 NCBI Blast:Nucleotide Sequence (800 letters)

Range 1: 690 to 1384 GenBank Graphics Next Match Previous Match

Score	Expect	Identities	Gaps	Strand
1284 bits(695)	0.0	695/695(100%)	0/695(0%)	Plus/Minus

```
Query  106   TGGCTTCTGGGGCCCTGGGGAGAGCACCACGGGGGTCGAGAGGCCATCCACGCTGATAGA  165
             ||||||||||||||||||||||||||||||||||||||||||||||||||||||||||||
Sbjct  1384  TGGCTTCTGGGGCCCTGGGGAGAGCACCACGGGGGTCGAGAGGCCATCCACGCTGATAGA  1325

Query  166   AGGGATGTGCACCTGGGCGCTGCCACTGGATGGAAACTGGAAGGAGAGCTTGGCCGGGCT  225
             ||||||||||||||||||||||||||||||||||||||||||||||||||||||||||||
Sbjct  1324  AGGGATGTGCACCTGGGCGCTGCCACTGGATGGAAACTGGAAGGAGAGCTTGGCCGGGCT  1265

Query  226   ACGGGGCGCAATGGGACTCAGGGTGCTCCAGAAGTGAATGCTAGGAGGCAGCGAGCTGGG  285
             ||||||||||||||||||||||||||||||||||||||||||||||||||||||||||||
Sbjct  1264  ACGGGGCGCAATGGGACTCAGGGTGCTCCAGAAGTGAATGCTAGGAGGCAGCGAGCTGGG  1205

Query  286   TGTCAGCAGCACCGGGGTCAATGTATGCGTAGGAAGCAGGGATGGGGTCAGCGCCGGCCC  345
             ||||||||||||||||||||||||||||||||||||||||||||||||||||||||||||
Sbjct  1204  TGTCAGCAGCACCGGGGTCAATGTATGCGTAGGAAGCAGGGATGGGGTCAGCGCCGGCCC  1145

Query  346   CGGAGCCTGGAGGCCGGAGCCACTTCCCGATCCTGGGGTCCGTTCGGGTCCCGGCCCACC  405
             ||||||||||||||||||||||||||||||||||||||||||||||||||||||||||||
Sbjct  1144  CGGAGCCTGGAGGCCGGAGCCACTTCCCGATCCTGGGGTCCGTTCGGGTCCCGGCCCACC  1085

Query  406   TAGCAGGCTCGGGCTGAGTGGAAGCTCTAGGTCCCGGGGCTTCCGGCCCTTCTGCGGCTG  465
             ||||||||||||||||||||||||||||||||||||||||||||||||||||||||||||
Sbjct  1084  TAGCAGGCTCGGGCTGAGTGGAAGCTCTAGGTCCCGGGGCTTCCGGCCCTTCTGCGGCTG  1025

Query  466   GGAGATCTCAGGGCTGGAAGCCGCATGGCCGCCCGCCTGCCCTGCGGTGTCCATAACAAC  525
             ||||||||||||||||||||||||||||||||||||||||||||||||||||||||||||
Sbjct  1024  GGAGATCTCAGGGCTGGAAGCCGCATGGCCGCCCGCCTGCCCTGCGGTGTCCATAACAAC  965

Query  526   CGCGGGCAGCCGGGCTGGCACGCCCTCCTGTGGAGGGACTTCTGGCTCGGCCTTGGTGGT  585
             ||||||||||||||||||||||||||||||||||||||||||||||||||||||||||||
Sbjct  964   CGCGGGCAGCCGGGCTGGCACGCCCTCCTGTGGAGGGACTTCTGGCTCGGCCTTGGTGGT  905

Query  586   TTCTGGCACAAACCCTCTCTCCCCCGCAACTTCCAACTCTTCCTTGGGCCCTTCTACTTT  645
             ||||||||||||||||||||||||||||||||||||||||||||||||||||||||||||
Sbjct  904   TTCTGGCACAAACCCTCTCTCCCCCGCAACTTCCAACTCTTCCTTGGGCCCTTCTACTTT  845

Query  646   CACTTCTGGGGGCAAAGCCCGGCCCAAACCCGGCTCCACATTAAGCTCTTCCGATTTCAG  705
             ||||||||||||||||||||||||||||||||||||||||||||||||||||||||||||
Sbjct  844   CACTTCTGGGGGCAAAGCCCGGCCCAAACCCGGCTCCACATTAAGCTCTTCCGATTTCAG  785

Query  706   GTTTGGGGCCTCGGGCGGGGTCAGGATGACCTGCAGAGGCAAGCCGGCCTCTTCAGCCTC  765
             ||||||||||||||||||||||||||||||||||||||||||||||||||||||||||||
Sbjct  784   GTTTGGGGCCTCGGGCGGGGTCAGGATGACCTGCAGAGGCAAGCCGGCCTCTTCAGCCTC  725

Query  766   CAGACAGGCCTCCAAGGGGCTTGGACTGGTGCTCC  800
             |||||||||||||||||||||||||||||||||||
Sbjct  724   CAGACAGGCCTCCAAGGGGCTTGGACTGGTGCTCC  690
```

Supplementary Figure S25: The down sequence (690 to 1384) of Elk-1 with a myc-tag (amino acids 145-428) (GenBank Accession No. AB016193, nucleotides 533 to 1384). (**A**) The sequencing data was present using the BGHR primer as initiator. (**B**) The “Query” (106 to 800) of sequence was aligned and identified by the NCBI BLAST.

A

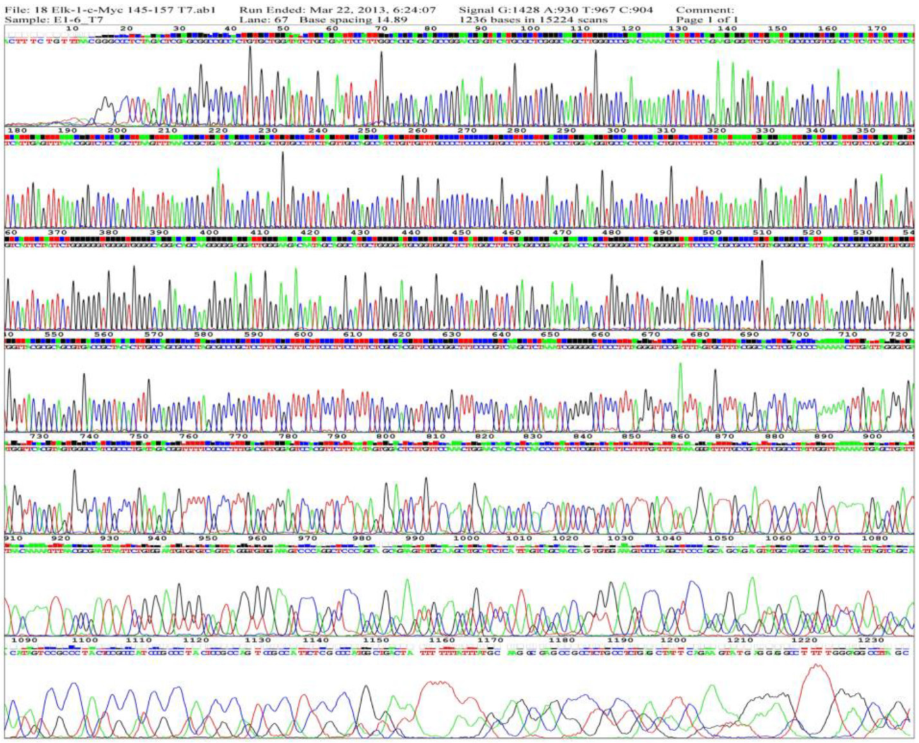

B

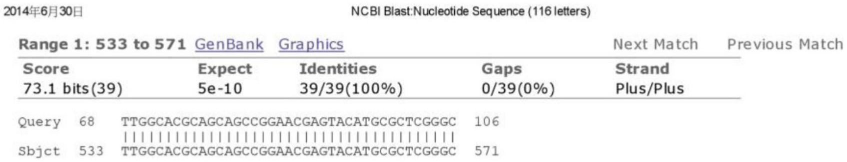

Supplementary Figure S26: Elk-1 with a myc-tag (amino acids 145-157) (GenBank Accession No. AB016193, nucleotides 533 to 571). (**A**) The sequencing data was present using the T7 primer as initiator. (**B**) The "Query" (68 to 106) of sequence was aligned and identified by the NCBI BLAST.

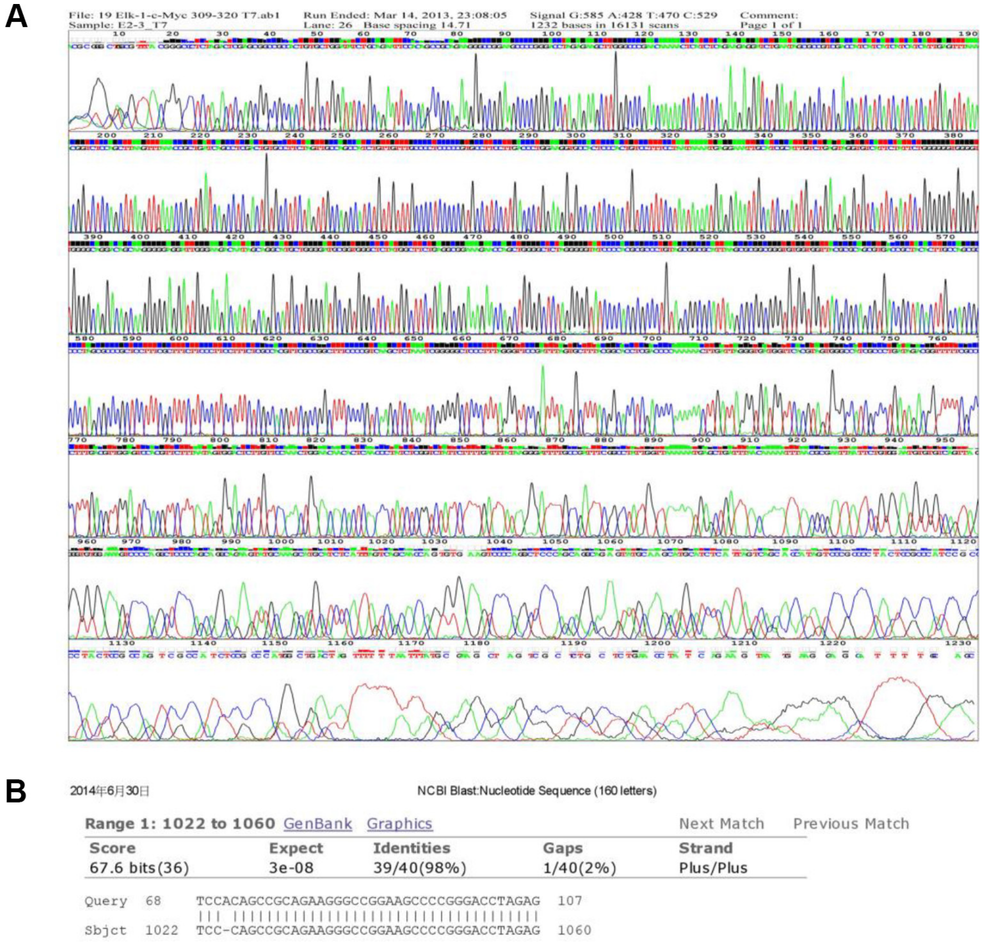

Supplementary Figure S27: Elk-1 with a myc-tag (amino acids 309-320) (GenBank Accession No. AB016193, nucleotides 1025 to 1060). (**A**) The sequencing data was present using the T7 primer as initiator. (**B**) The "Query" (72 to 107) of sequence was aligned and identified by the NCBI BLAST.

A

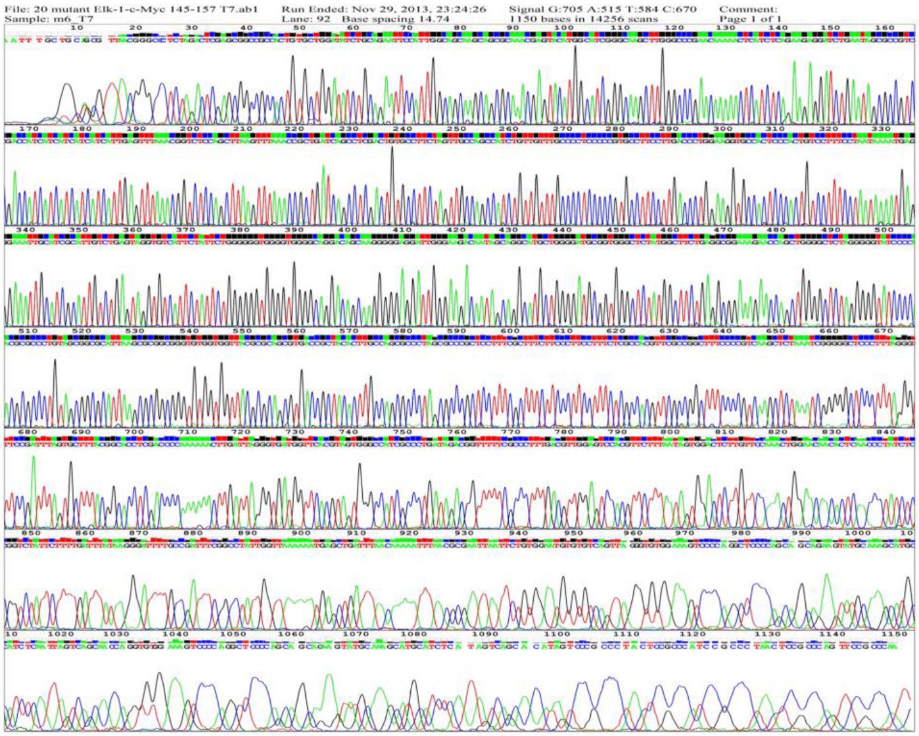

B

```
Query  73   TTGGCAGCAAGCAGCGCAAACGAGTACATGGCATCGGGC  111
            ||||||   ||||||   ||||||||||||   ||||||
Sbjct  533  TTGGCACGCAGCAGCCGGAACGAGTACATGCGCTCGGGC  571
```

Supplementary Figure S28: Mutant Elk-1 with a myc-tag (amino acids 145-157) (GenBank Accession No. AB016193, nucleotides 533 to 571). (**A**) The sequencing data was present using the T7 primer as initiator. (**B**) The "Query" (73 to 111) of sequence was aligned and identified by the NCBI BLAST.

A

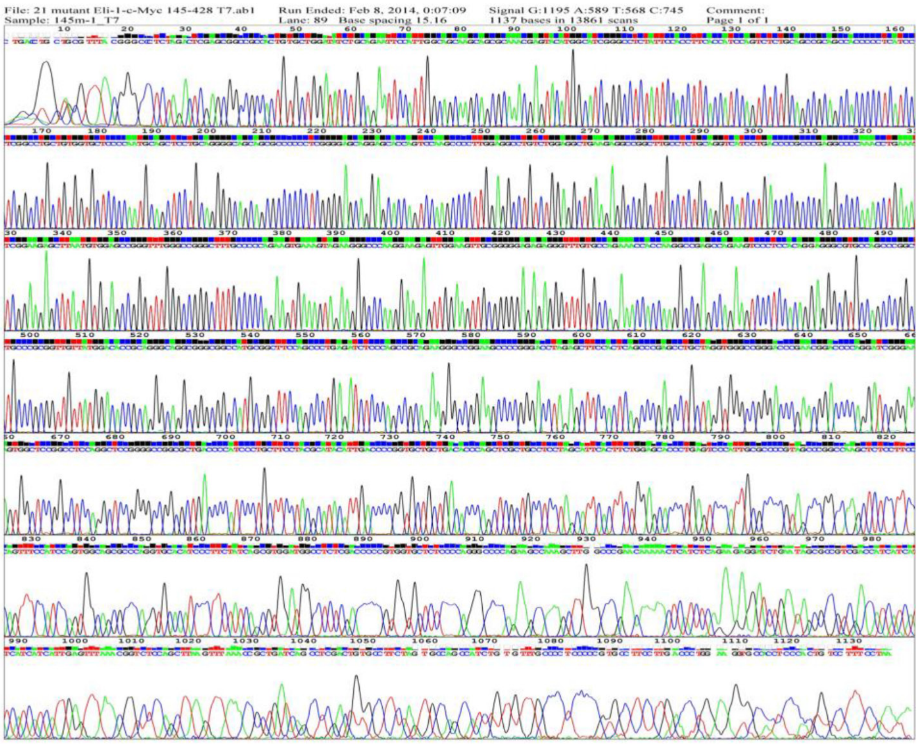

B

```
2014年6月30日                          NCBI Blast:Nucleotide Sequence (800 letters)

Range 1: 533 to 1261  GenBank  Graphics                      Next Match   Previous Match
 Score            Expect    Identities       Gaps         Strand
 1308 bits(708)   0.0       724/731(99%)     4/731(0%)    Plus/Plus

Query  72    TTGGCA-GCAAGCAGCGCAAACGAGTACATG-GCATCGGGCCTCTATTCCACCTTCACCA  129
             |||||| ||  ||||||    ||||||||||||| ||  |||||||||||||||||||||||||||
Sbjct  533   TTGGCACGC-AGCAGCCGGAACGAGTACATGCGC-TCGGGCCTCTATTCCACCTTCACCA  590

Query  130   TCCAGTCTCTGCAGCCGCAGCCACCCCCTCATCCTCGGCCTGCTGTGGTGCTCCCCAATG  189
             ||||||||||||||||||||||||||||||||||||||||||||||||||||||||||||
Sbjct  591   TCCAGTCTCTGCAGCCGCAGCCACCCCCTCATCCTCGGCCTGCTGTGGTGCTCCCCAATG  650

Query  190   CAGCTCCTGCAGGGGCAGCAGCGCCCCCCTCGGGGAGCAGGAGCACCAGTCCAAGCCCCT  249
             ||||||||||||||||||||||||||||||||||||||||||||||||||||||||||||
Sbjct  651   CAGCTCCTGCAGGGGCAGCAGCGCCCCCCTCGGGGAGCAGGAGCACCAGTCCAAGCCCCT  710

Query  250   TGGAGGCCTGTCTGGAGGCTGAAGAGGCCGGCTTGCCTCTGCAGGTCATCCTGACCCCGC  309
             ||||||||||||||||||||||||||||||||||||||||||||||||||||||||||||
Sbjct  711   TGGAGGCCTGTCTGGAGGCTGAAGAGGCCGGCTTGCCTCTGCAGGTCATCCTGACCCCGC  770

Query  310   CCGAGGCCCCAAACCTGAAATCGGAAGAGCTTAATGTGGAGCCGGGTTTGGGCCGGGCTT  369
             ||||||||||||||||||||||||||||||||||||||||||||||||||||||||||||
Sbjct  771   CCGAGGCCCCAAACCTGAAATCGGAAGAGCTTAATGTGGAGCCGGGTTTGGGCCGGGCTT  830

Query  370   TGCCCCCAGAAGTGAAAGTAGAAGGGCCCAAGGAAGAGTTGGAAGTTGCGGGGGAGAGAG  429
             ||||||||||||||||||||||||||||||||||||||||||||||||||||||||||||
Sbjct  831   TGCCCCCAGAAGTGAAAGTAGAAGGGCCCAAGGAAGAGTTGGAAGTTGCGGGGGAGAGAG  890

Query  430   GGTTTGTGCCAGAAACCACCAAGGCCGAGCCAGAAGTCCCTCCACAGGAGGGCGTGCCAG  489
             ||||||||||||||||||||||||||||||||||||||||||||||||||||||||||||
Sbjct  891   GGTTTGTGCCAGAAACCACCAAGGCCGAGCCAGAAGTCCCTCCACAGGAGGGCGTGCCAG  950

Query  490   CCCGGCTGCCCGCGGTTGTTATGGACACCGCAGGGCAGGCGGGCGGCCATGCGGCTTCCA  549
             ||||||||||||||||||||||||||||||||||||||||||||||||||||||||||||
Sbjct  951   CCCGGCTGCCCGCGGTTGTTATGGACACCGCAGGGCAGGCGGGCGGCCATGCGGCTTCCA  1010

Query  550   GCCCTGAGATCTCCCAGCCGCAGAAGGGCCGGAAGCCCCGGGACCTAGAGCTTCCACTCA  609
             ||||||||||||||||||||||||||||||||||||||||||||||||||||||||||||
Sbjct  1011  GCCCTGAGATCTCCCAGCCGCAGAAGGGCCGGAAGCCCCGGGACCTAGAGCTTCCACTCA  1070

Query  610   GCCCGAGCCTGCTAGGTGGGCCGGGACCCGAACGGACCCCAGGATCGGGAAGTGGCTCCG  669
             ||||||||||||||||||||||||||||||||||||||||||||||||||||||||||||
Sbjct  1071  GCCCGAGCCTGCTAGGTGGGCCGGGACCCGAACGGACCCCAGGATCGGGAAGTGGCTCCG  1130

Query  670   GCCTCCAGGCTCCGGGGCCGGCGCTGACCCCATCCCTGCTTCCTACGCATACATTGACCC  729
             ||||||||||||||||||||||||||||||||||||||||||||||||||||||||||||
Sbjct  1131  GCCTCCAGGCTCCGGGGCCGGCGCTGACCCCATCCCTGCTTCCTACGCATACATTGACCC  1190

Query  730   CGGTGCTGCTGACACCCAGCTCGCTGCCTCCTAGCATTCACTTCTGGAGCACCCTGAGTC  789
             ||||||||||||||||||||||||||||||||||||||||||||||||||||||||||||
Sbjct  1191  CGGTGCTGCTGACACCCAGCTCGCTGCCTCCTAGCATTCACTTCTGGAGCACCCTGAGTC  1250

Query  790   CCATTGCGCCC  800
             |||||||||||
Sbjct  1251  CCATTGCGCCC  1261
```

Supplementary Figure S29: The up sequence (533 to 1261) of Mutant Elk-1 with a myc-tag (amino acids 145-428) (GenBank Accession No. AB016193, nucleotides 533 to 1384). (**A**) The sequencing data was present using the T7 primer as initiator. (**B**) The "Query" (72 to 800) of sequence was aligned and identified by the NCBI BLAST.

A

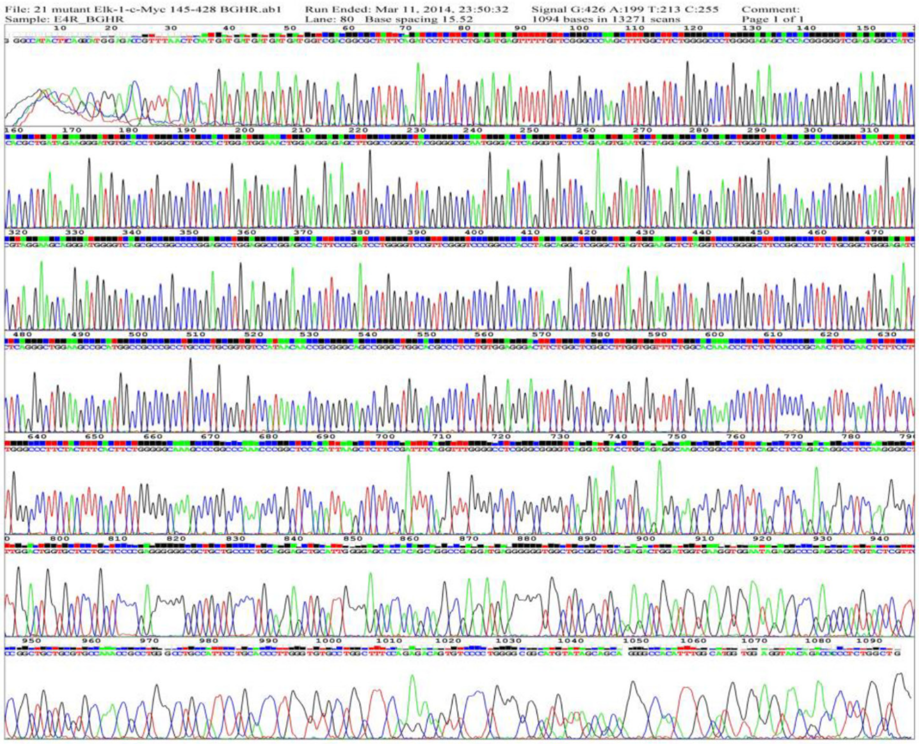

B

2014年6月30日 NCBI Blast:Nucleotide Sequence (640 letters)

Range 1: 855 to 1384 GenBank Graphics Next Match Previous Match

Score	Expect	Identities	Gaps	Strand
979 bits(530)	0.0	530/530(100%)	0/530(0%)	Plus/Minus

```
Query  111   TGGCTTCTGGGGCCCTGGGGAGAGCACCACGGGGGTCGAGAGGCCATCCACGCTGATAGA  170
             ||||||||||||||||||||||||||||||||||||||||||||||||||||||||||||
Sbjct  1384  TGGCTTCTGGGGCCCTGGGGAGAGCACCACGGGGGTCGAGAGGCCATCCACGCTGATAGA  1325

Query  171   AGGGATGTGCACCTGGGCGCTGCCACTGGATGGAAACTGGAAGGAGAGCTTGGCCGGGCT  230
             ||||||||||||||||||||||||||||||||||||||||||||||||||||||||||||
Sbjct  1324  AGGGATGTGCACCTGGGCGCTGCCACTGGATGGAAACTGGAAGGAGAGCTTGGCCGGGCT  1265

Query  231   ACGGGGCGCAATGGGACTCAGGGTGCTCCAGAAGTGAATGCTAGGAGGCAGCGAGCTGGG  290
             ||||||||||||||||||||||||||||||||||||||||||||||||||||||||||||
Sbjct  1264  ACGGGGCGCAATGGGACTCAGGGTGCTCCAGAAGTGAATGCTAGGAGGCAGCGAGCTGGG  1205

Query  291   TGTCAGCAGCACCGGGGTCAATGTATGCGTAGGAAGCAGGGATGGGGTCAGCGCCGGCCC  350
             ||||||||||||||||||||||||||||||||||||||||||||||||||||||||||||
Sbjct  1204  TGTCAGCAGCACCGGGGTCAATGTATGCGTAGGAAGCAGGGATGGGGTCAGCGCCGGCCC  1145

Query  351   CGGAGCCTGGAGGCCGGAGCCACTTCCCGATCCTGGGGTCCGTTCGGGTCCCGGCCCACC  410
             ||||||||||||||||||||||||||||||||||||||||||||||||||||||||||||
Sbjct  1144  CGGAGCCTGGAGGCCGGAGCCACTTCCCGATCCTGGGGTCCGTTCGGGTCCCGGCCCACC  1085

Query  411   TAGCAGGCTCGGGCTGAGTGGAAGCTCTAGGTCCCGGGGCTTCCGGCCCTTCTGCGGCTG  470
             ||||||||||||||||||||||||||||||||||||||||||||||||||||||||||||
Sbjct  1084  TAGCAGGCTCGGGCTGAGTGGAAGCTCTAGGTCCCGGGGCTTCCGGCCCTTCTGCGGCTG  1025

Query  471   GGAGATCTCAGGGCTGGAAGCCGCATGGCCGCCCGCCTGCCCTGCGGTGTCCATAACAAC  530
             ||||||||||||||||||||||||||||||||||||||||||||||||||||||||||||
Sbjct  1024  GGAGATCTCAGGGCTGGAAGCCGCATGGCCGCCCGCCTGCCCTGCGGTGTCCATAACAAC  965

Query  531   CGCGGGCAGCCGGGCTGGCACGCCCTCCTGTGGAGGGACTTCTGGCTCGGCCTTGGTGGT  590
             ||||||||||||||||||||||||||||||||||||||||||||||||||||||||||||
Sbjct  964   CGCGGGCAGCCGGGCTGGCACGCCCTCCTGTGGAGGGACTTCTGGCTCGGCCTTGGTGGT  905

Query  591   TTCTGGCACAAACCCTCTCTCCCCCGCAACTTCCAACTCTTCCTTGGGCC  640
             ||||||||||||||||||||||||||||||||||||||||||||||||||
Sbjct  904   TTCTGGCACAAACCCTCTCTCCCCCGCAACTTCCAACTCTTCCTTGGGCC  855
```

Supplementary Figure S30: The down sequence (855 to 1384) of Mutant Elk-1 with a myc-tag (amino acids 145-428) (GenBank Accession No. AB016193, nucleotides 533 to 1384). (**A**) The sequencing data was present using the BGHR primer as initiator. (**B**) The "Query" (111 to 640) of sequence was aligned and identified by the NCBI BLAST.

A

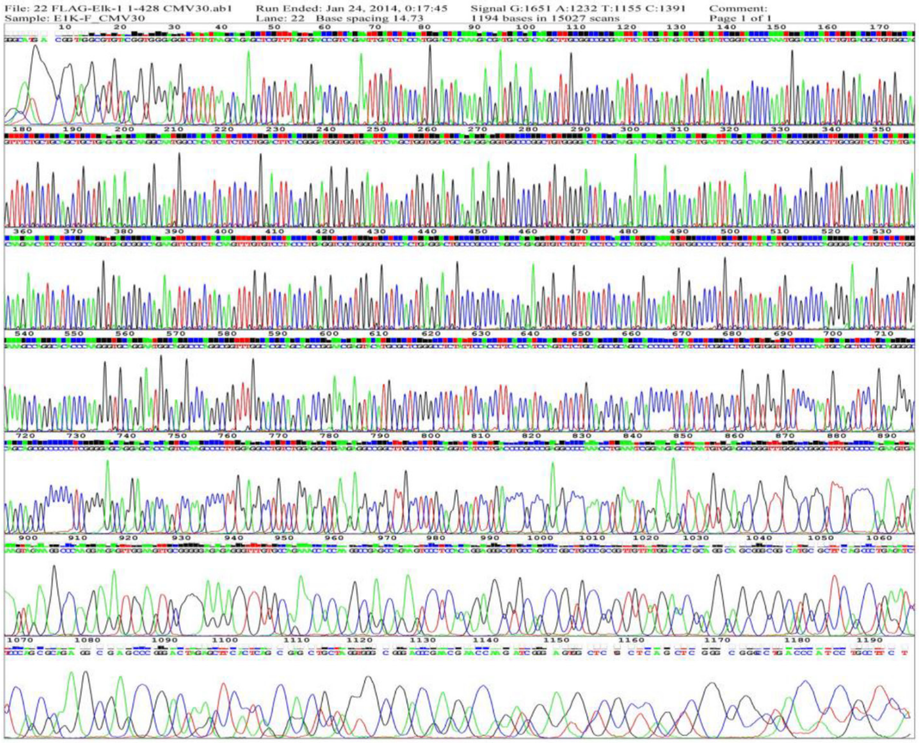

B

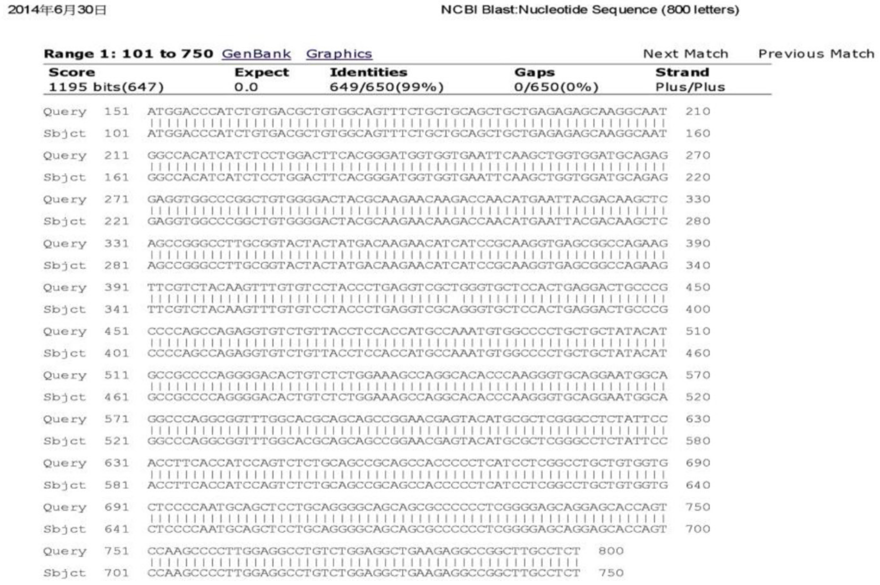

2014年6月30日 NCBI Blast:Nucleotide Sequence (800 letters)

Range 1: 101 to 750 GenBank Graphics Next Match Previous Match

Score	Expect	Identities	Gaps	Strand
1195 bits(647)	0.0	649/650(99%)	0/650(0%)	Plus/Plus

```
Query  151  ATGGACCCATCTGTGACGCTGTGGCAGTTTCTGCTGCAGCTGCTGAGAGAGCAAGGCAAT  210
            ||||||||||||||||||||||||||||||||||||||||||||||||||||||||||||
Sbjct  101  ATGGACCCATCTGTGACGCTGTGGCAGTTTCTGCTGCAGCTGCTGAGAGAGCAAGGCAAT  160

Query  211  GGCCACATCATCTCCTGGACTTCACGGGATGGTGGTGAATTCAAGCTGGTGGATGCAGAG  270
            ||||||||||||||||||||||||||||||||||||||||||||||||||||||||||||
Sbjct  161  GGCCACATCATCTCCTGGACTTCACGGGATGGTGGTGAATTCAAGCTGGTGGATGCAGAG  220

Query  271  GAGGTGGCCCGGCTGTGGGGACTACGCAAGAACAAGACCAACATGAATTACGACAAGCTC  330
            ||||||||||||||||||||||||||||||||||||||||||||||||||||||||||||
Sbjct  221  GAGGTGGCCCGGCTGTGGGGACTACGCAAGAACAAGACCAACATGAATTACGACAAGCTC  280

Query  331  AGCCGGGCCTTGCGGTACTACTATGACAAGAACATCATCCGCAAGGTGAGCGGCCAGAAG  390
            ||||||||||||||||||||||||||||||||||||||||||||||||||||||||||||
Sbjct  281  AGCCGGGCCTTGCGGTACTACTATGACAAGAACATCATCCGCAAGGTGAGCGGCCAGAAG  340

Query  391  TTCGTCTACAAGTTTGTGTCCTACCCTGAGGTCGCTGGGTGCTCCACTGAGGACTGCCCG  450
            |||||||||||||||||||||||||||||||||| |||||||||||||||||||||||||
Sbjct  341  TTCGTCTACAAGTTTGTGTCCTACCCTGAGGTCGCAGGGTGCTCCACTGAGGACTGCCCG  400

Query  451  CCCCAGCCAGAGGTGTCTGTTACCTCCACCATGCCAAATGTGGCCCCTGCTGCTATACAT  510
            ||||||||||||||||||||||||||||||||||||||||||||||||||||||||||||
Sbjct  401  CCCCAGCCAGAGGTGTCTGTTACCTCCACCATGCCAAATGTGGCCCCTGCTGCTATACAT  460

Query  511  GCCGCCCCAGGGGACACTGTCTCTGGAAAGCCAGGCACACCCAAGGGTGCAGGAATGGCA  570
            ||||||||||||||||||||||||||||||||||||||||||||||||||||||||||||
Sbjct  461  GCCGCCCCAGGGGACACTGTCTCTGGAAAGCCAGGCACACCCAAGGGTGCAGGAATGGCA  520

Query  571  GGCCCAGGCGGTTTGGCACGCAGCAGCCGGAACGAGTACATGCGCTCGGGCCTCTATTCC  630
            ||||||||||||||||||||||||||||||||||||||||||||||||||||||||||||
Sbjct  521  GGCCCAGGCGGTTTGGCACGCAGCAGCCGGAACGAGTACATGCGCTCGGGCCTCTATTCC  580

Query  631  ACCTTCACCATCCAGTCTCTGCAGCCGCAGCCACCCCCTCATCCTCGGCCTGCTGTGGTG  690
            ||||||||||||||||||||||||||||||||||||||||||||||||||||||||||||
Sbjct  581  ACCTTCACCATCCAGTCTCTGCAGCCGCAGCCACCCCCTCATCCTCGGCCTGCTGTGGTG  640

Query  691  CTCCCCAATGCAGCTCCTGCAGGGGCAGCAGCGCCCCCCTCGGGGAGCAGGAGCACCAGT  750
            ||||||||||||||||||||||||||||||||||||||||||||||||||||||||||||
Sbjct  641  CTCCCCAATGCAGCTCCTGCAGGGGCAGCAGCGCCCCCCTCGGGGAGCAGGAGCACCAGT  700

Query  751  CCAAGCCCCTTGGAGGCCTGTCTGGAGGCTGAAGAGGCCGGCTTGCCTCT  800
            ||||||||||||||||||||||||||||||||||||||||||||||||||
Sbjct  701  CCAAGCCCCTTGGAGGCCTGTCTGGAGGCTGAAGAGGCCGGCTTGCCTCT  750
```

Supplementary Figure S31: The up sequence (101 to 750) of Elk-1 with a FLAG-tag (amino acids 1-428) (GenBank Accession No. AB016193, nucleotides 101 to 1384). (**A**) The sequencing data was present using the CMV30 primer as initiator. (**B**) The "Query" (151 to 800) of the sequence was aligned and identified by the NCBI BLAST.

A

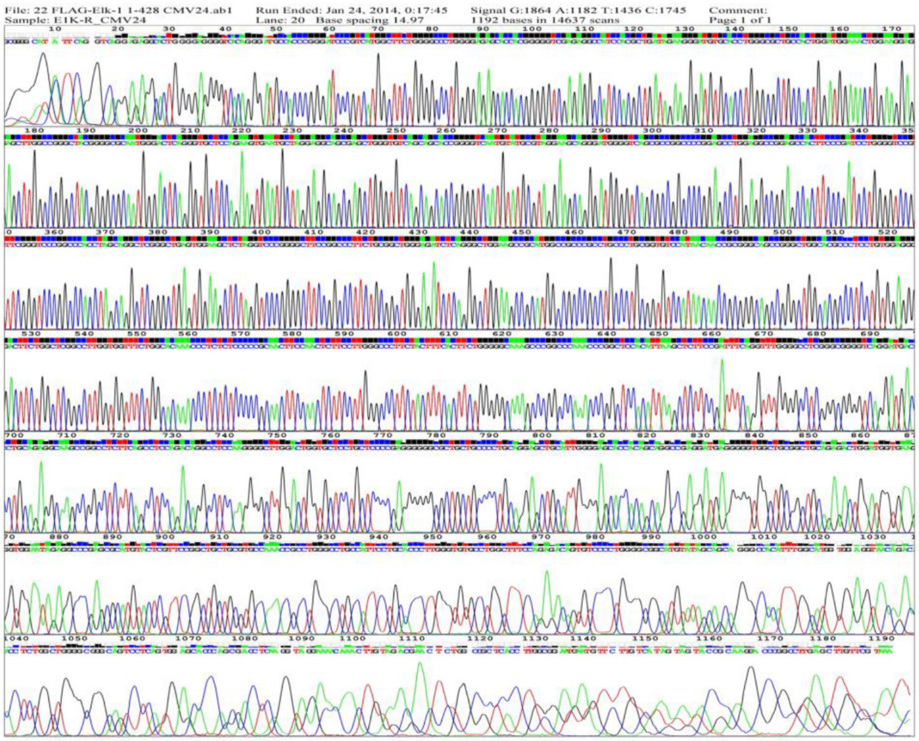

B

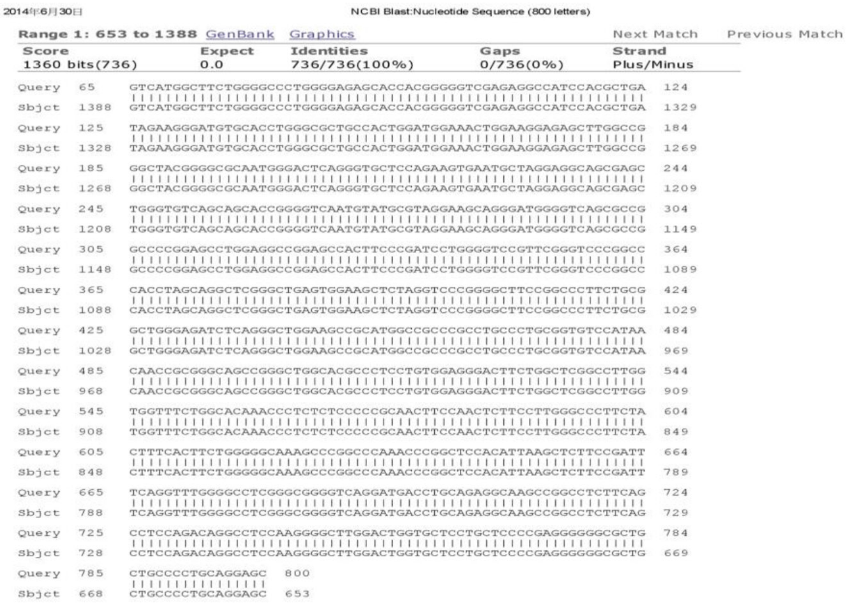

2014年6月30日 NCBI Blast:Nucleotide Sequence (800 letters)

Range 1: 653 to 1388 GenBank Graphics Next Match Previous Match

Score	Expect	Identities	Gaps	Strand
1360 bits(736)	0.0	736/736(100%)	0/736(0%)	Plus/Minus

```
Query  65    GTCATGGCTTCTGGGGCCCTGGGGAGAGCACCACGGGGGTCGAGAGGCCATCCACGCTGA  124
             ||||||||||||||||||||||||||||||||||||||||||||||||||||||||||||
Sbjct  1388  GTCATGGCTTCTGGGGCCCTGGGGAGAGCACCACGGGGGTCGAGAGGCCATCCACGCTGA  1329

Query  125   TAGAAGGGATGTGCACCTGGGCGCTGCCACTGGATGGAAACTGGAAGGAGAGCTTGGCCG  184
             ||||||||||||||||||||||||||||||||||||||||||||||||||||||||||||
Sbjct  1328  TAGAAGGGATGTGCACCTGGGCGCTGCCACTGGATGGAAACTGGAAGGAGAGCTTGGCCG  1269

Query  185   GGCTACGGGGCGCAATGGGACTCAGGGTGCTCCAGAAGTGAATGCTAGGAGGCAGCGAGC  244
             ||||||||||||||||||||||||||||||||||||||||||||||||||||||||||||
Sbjct  1268  GGCTACGGGGCGCAATGGGACTCAGGGTGCTCCAGAAGTGAATGCTAGGAGGCAGCGAGC  1209

Query  245   TGGGTGTCAGCAGCACCGGGGTCAATGTATGCGTAGGAAGCAGGGATGGGGTCAGCGCCG  304
             ||||||||||||||||||||||||||||||||||||||||||||||||||||||||||||
Sbjct  1208  TGGGTGTCAGCAGCACCGGGGTCAATGTATGCGTAGGAAGCAGGGATGGGGTCAGCGCCG  1149

Query  305   GCCCCGGAGCCTGGAGGCCGGAGCCACTTCCCGATCCTGGGGTCCGTTCGGGTCCCGGCC  364
             ||||||||||||||||||||||||||||||||||||||||||||||||||||||||||||
Sbjct  1148  GCCCCGGAGCCTGGAGGCCGGAGCCACTTCCCGATCCTGGGGTCCGTTCGGGTCCCGGCC  1089

Query  365   CACCTAGCAGGCTCGGGCTGAGTGGAAGCTCTAGGTCCCGGGGCTTCCGGCCCTTCTGCG  424
             ||||||||||||||||||||||||||||||||||||||||||||||||||||||||||||
Sbjct  1088  CACCTAGCAGGCTCGGGCTGAGTGGAAGCTCTAGGTCCCGGGGCTTCCGGCCCTTCTGCG  1029

Query  425   GCTGGGAGATCTCAGGGCTGGAAGCCGCATGGCCGCCCGCCTGCCCTGCGGTGTCCATAA  484
             ||||||||||||||||||||||||||||||||||||||||||||||||||||||||||||
Sbjct  1028  GCTGGGAGATCTCAGGGCTGGAAGCCGCATGGCCGCCCGCCTGCCCTGCGGTGTCCATAA  969

Query  485   CAACCGCGGGCAGCCGGGCTGGCACGCCCTCCTGTGGAGGGACTTCTGGCTCGGCCTTGG  544
             ||||||||||||||||||||||||||||||||||||||||||||||||||||||||||||
Sbjct  968   CAACCGCGGGCAGCCGGGCTGGCACGCCCTCCTGTGGAGGGACTTCTGGCTCGGCCTTGG  909

Query  545   TGGTTTCTGGCACAAACCCTCTCTCCCCCGCAACTTCCAACTCTTCCTTGGGCCCTTCTA  604
             ||||||||||||||||||||||||||||||||||||||||||||||||||||||||||||
Sbjct  908   TGGTTTCTGGCACAAACCCTCTCTCCCCCGCAACTTCCAACTCTTCCTTGGGCCCTTCTA  849

Query  605   CTTTCACTTCTGGGGGCAAAGCCCGGCCCAAACCCGGCTCCACATTAAGCTCTTCCGATT  664
             ||||||||||||||||||||||||||||||||||||||||||||||||||||||||||||
Sbjct  848   CTTTCACTTCTGGGGGCAAAGCCCGGCCCAAACCCGGCTCCACATTAAGCTCTTCCGATT  789

Query  665   TCAGGTTTGGGGCCTCGGGCGGGGTCAGGATGACCTGCAGAGGCAAGCCGGCCTCTTCAG  724
             ||||||||||||||||||||||||||||||||||||||||||||||||||||||||||||
Sbjct  788   TCAGGTTTGGGGCCTCGGGCGGGGTCAGGATGACCTGCAGAGGCAAGCCGGCCTCTTCAG  729

Query  725   CCTCCAGACAGGCCTCCAAGGGGCTTGGACTGGTGCTCCTGCTCCCCGAGGGGGGCGCTG  784
             ||||||||||||||||||||||||||||||||||||||||||||||||||||||||||||
Sbjct  728   CCTCCAGACAGGCCTCCAAGGGGCTTGGACTGGTGCTCCTGCTCCCCGAGGGGGGCGCTG  669

Query  785   CTGCCCCTGCAGGAGC  800
             ||||||||||||||||
Sbjct  668   CTGCCCCTGCAGGAGC  653
```

Supplementary Figure S32: The down sequence (653 to 1387) of Elk-1 with a FLAG-tag (amino acids 1-428) (GenBank Accession No. AB016193, nucleotides 101 to 1384). (**A**) The sequencing data was present using the CMV24 primer as initiator. (**B**) The "Query" (66 to 800) of the sequence was aligned and identified by the NCBI BLAST.

A

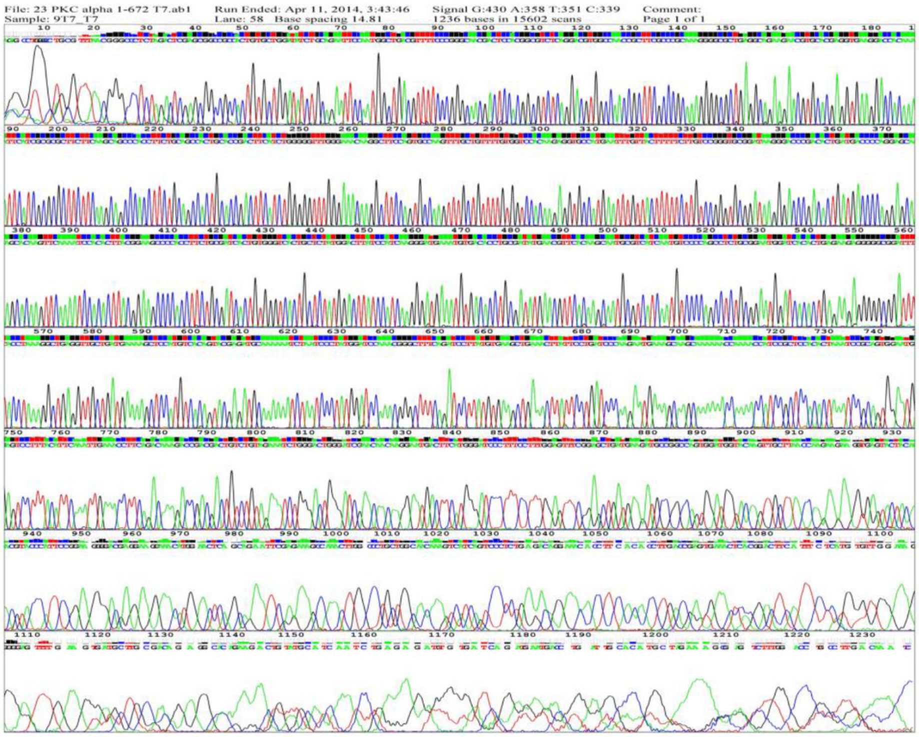

B

2014年6月30日 NCBI Blast:Nucleotide Sequence (960 letters)

Range 1: 45 to 929 GenBank Graphics Next Match Previous Match

Score	Expect	Identities	Gaps	Strand
1635 bits(885)	0.0	885/885(100%)	0/885(0%)	Plus/Plus

```
Query  76   ATGGCTGACGTTTTCCCGGGCAACGACTCCACGGCGTCTCAGGACGTGGCCAACCGCTTC  135
            ||||||||||||||||||||||||||||||||||||||||||||||||||||||||||||
Sbjct  45   ATGGCTGACGTTTTCCCGGGCAACGACTCCACGGCGTCTCAGGACGTGGCCAACCGCTTC  104

Query  136  GCCCGCAAAGGGGCGCTGAGGCAGAAGAACGTGCACGAGGTGAAGGACCACAAATTCATC  195
            ||||||||||||||||||||||||||||||||||||||||||||||||||||||||||||
Sbjct  105  GCCCGCAAAGGGGCGCTGAGGCAGAAGAACGTGCACGAGGTGAAGGACCACAAATTCATC  164

Query  196  GCGCGCTTCTTCAAGCAGCCCACCTTCTGCAGCCACTGCACCGACTTCATCTGGGGGTTT  255
            ||||||||||||||||||||||||||||||||||||||||||||||||||||||||||||
Sbjct  165  GCGCGCTTCTTCAAGCAGCCCACCTTCTGCAGCCACTGCACCGACTTCATCTGGGGGTTT  224

Query  256  GGGAAACAAGGCTTCCAGTGCCAAGTTTGCTGTTTTGTGGTCCACAAGAGGTGCCATGAA  315
            ||||||||||||||||||||||||||||||||||||||||||||||||||||||||||||
Sbjct  225  GGGAAACAAGGCTTCCAGTGCCAAGTTTGCTGTTTTGTGGTCCACAAGAGGTGCCATGAA  284

Query  316  TTTGTTACTTTTTCTTGTCCGGGTGCGGATAAGGGACCCGACACTGATGACCCCAGGAGC  375
            ||||||||||||||||||||||||||||||||||||||||||||||||||||||||||||
Sbjct  285  TTTGTTACTTTTTCTTGTCCGGGTGCGGATAAGGGACCCGACACTGATGACCCCAGGAGC  344

Query  376  AAGCACAAGTTCAAAATCCACACTTACGGAAGCCCCACCTTCTGCGATCACTGTGGGTCA  435
            ||||||||||||||||||||||||||||||||||||||||||||||||||||||||||||
Sbjct  345  AAGCACAAGTTCAAAATCCACACTTACGGAAGCCCCACCTTCTGCGATCACTGTGGGTCA  404

Query  436  CTGCTCTATGGACTTATCCATCAAGGGATGAAATGTGACACCTGCGATATGAACGTTCAC  495
            ||||||||||||||||||||||||||||||||||||||||||||||||||||||||||||
Sbjct  405  CTGCTCTATGGACTTATCCATCAAGGGATGAAATGTGACACCTGCGATATGAACGTTCAC  464

Query  496  AAGCAATGCGTCATCAATGTCCCCAGCCTCTGCGGAATGGATCACACTGAGAAGAGGGGG  555
            ||||||||||||||||||||||||||||||||||||||||||||||||||||||||||||
Sbjct  465  AAGCAATGCGTCATCAATGTCCCCAGCCTCTGCGGAATGGATCACACTGAGAAGAGGGGG  524

Query  556  CGGATTTACCTAAAGGCTGAGGTTGCTGATGAAAAGCTCCATGTCACAGTACGAGATGCA  615
            ||||||||||||||||||||||||||||||||||||||||||||||||||||||||||||
Sbjct  525  CGGATTTACCTAAAGGCTGAGGTTGCTGATGAAAAGCTCCATGTCACAGTACGAGATGCA  584

Query  616  AAAAATCTAATCCCTATGGATCCAAACGGGCTTTCAGATCCTTATGTGAAGCTGAAACTT  675
            ||||||||||||||||||||||||||||||||||||||||||||||||||||||||||||
Sbjct  585  AAAAATCTAATCCCTATGGATCCAAACGGGCTTTCAGATCCTTATGTGAAGCTGAAACTT  644

Query  676  ATTCCTGATCCCAAGAATGAAAGCAAGCAAAAAACCAAAACCATCCGCTCCACACTAAAT  735
            ||||||||||||||||||||||||||||||||||||||||||||||||||||||||||||
Sbjct  645  ATTCCTGATCCCAAGAATGAAAGCAAGCAAAAAACCAAAACCATCCGCTCCACACTAAAT  704

Query  736  CCGCAGTGGAATGAGTCCTTTACATTCAAATTGAAACCTTCAGACAAAGACCGACGACTG  795
            ||||||||||||||||||||||||||||||||||||||||||||||||||||||||||||
Sbjct  705  CCGCAGTGGAATGAGTCCTTTACATTCAAATTGAAACCTTCAGACAAAGACCGACGACTG  764

Query  796  TCTGTAGAAATCTGGGACTGGGATCGAACAACAAGGAATGACTTCATGGGATCCCTTTCC  855
            ||||||||||||||||||||||||||||||||||||||||||||||||||||||||||||
Sbjct  765  TCTGTAGAAATCTGGGACTGGGATCGAACAACAAGGAATGACTTCATGGGATCCCTTTCC  824

Query  856  TTTGGAGTTTCGGAGCTGATGAAGATGCCGGCCAGTGGATGGTACAAGTTGCTTAACCAA  915
            ||||||||||||||||||||||||||||||||||||||||||||||||||||||||||||
Sbjct  825  TTTGGAGTTTCGGAGCTGATGAAGATGCCGGCCAGTGGATGGTACAAGTTGCTTAACCAA  884

Query  916  GAAGAAGGTGAGTACTACAACGTACCCATTCCGGAAGGGGACGAG  960
            |||||||||||||||||||||||||||||||||||||||||||||
Sbjct  885  GAAGAAGGTGAGTACTACAACGTACCCATTCCGGAAGGGGACGAG  929
```

Supplementary Figure S33:The up sequence (45 to 929) of PKCα (1-672 aa) with a myc-tag (amino acids 1-672) (GenBank Accession No. NM_002737, nucleotides 45 to 2060). (**A**) The sequencing data was present using the T7 primer as initiator. (**B**) The “Query” (76 to 960) of the sequence was aligned and identified by the NCBI BLAST.

A

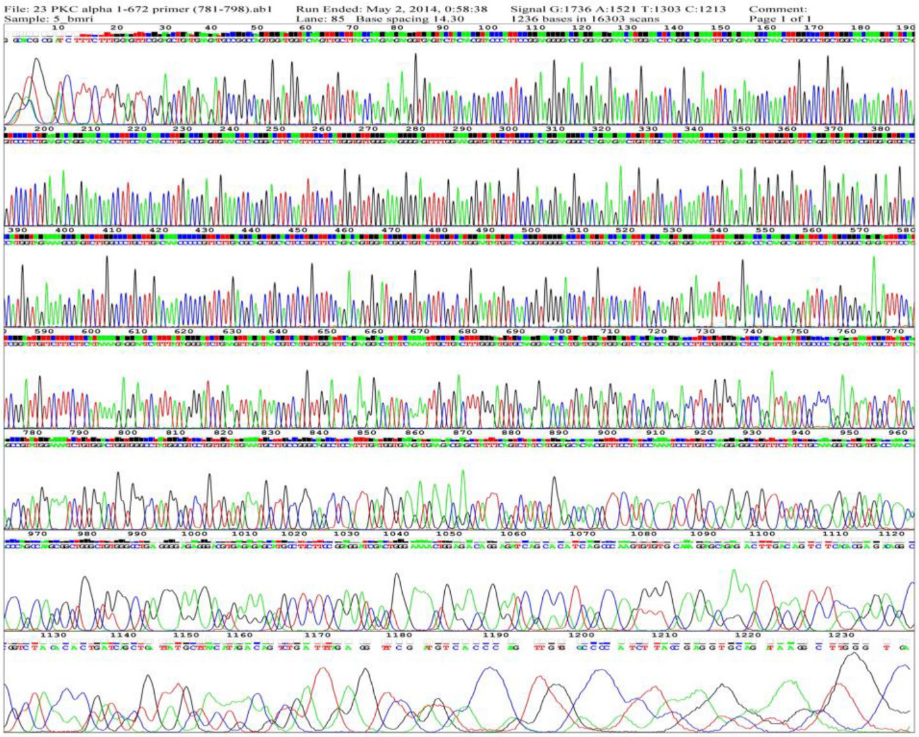

B

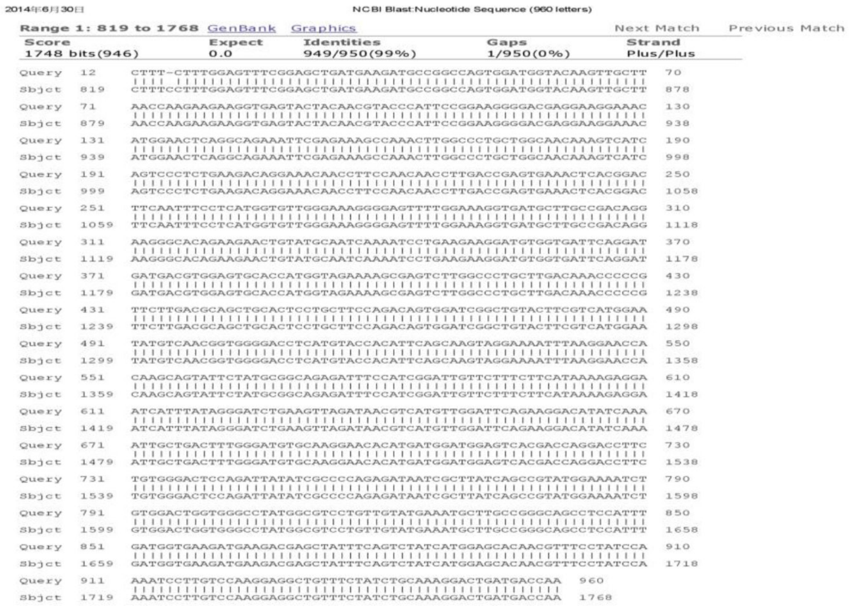

Supplementary Figure S34: The middle sequence (824 to 1768) of PKCα (1-672 aa) with a myc-tag (amino acids 1-672) (GenBank Accession No. NM_002737, nucleotides 45 to 2060). (**A**) The sequencing data was present using the one PKCα sequence (781 to 798; ACTGGGATCGAACAACAA) primer as initiator. (**B**) The "Query" (16 to 960) of the sequence was aligned and identified by the NCBI BLAST.

A

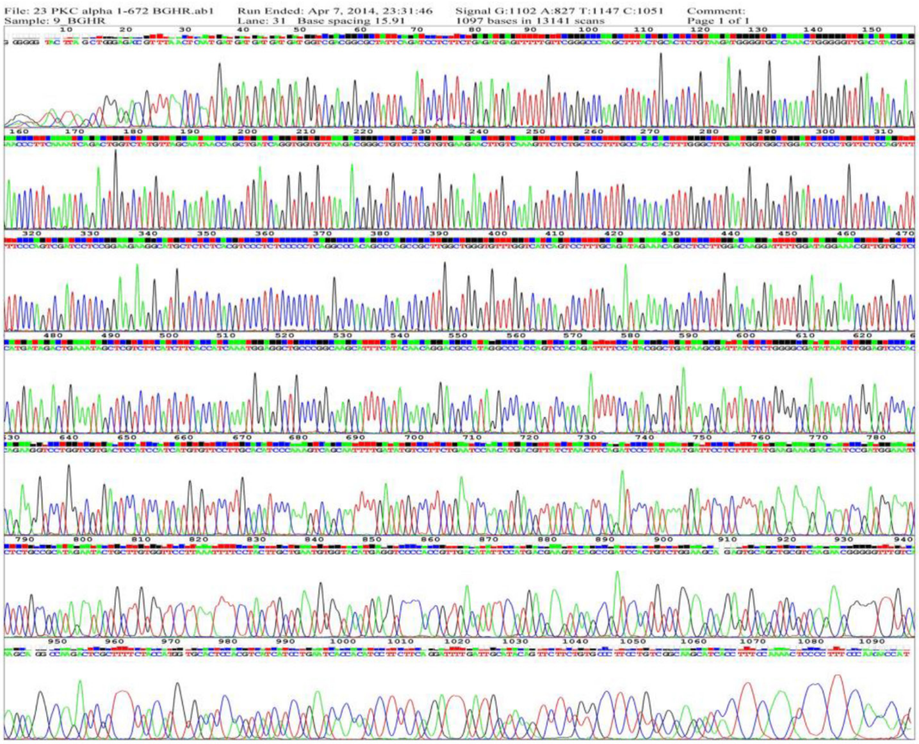

B

```
2014年6月30日                NCBI Blast:Nucleotide Sequence (800 letters)

Range 1: 1369 to 2060 GenBank Graphics              Next Match    Previous Match
Score            Expect   Identities      Gaps        Strand
1279 bits(692)   0.0      692/692(100%)   0/692(0%)   Plus/Minus

Query  109   TACTGCACTCTGTAAGATGGGGTGCACAAACTGGGGGTTGACATACGAGAACCCTTCAAA  168
             ||||||||||||||||||||||||||||||||||||||||||||||||||||||||||||
Sbjct  2060  TACTGCACTCTGTAAGATGGGGTGCACAAACTGGGGGTTGACATACGAGAACCCTTCAAA  2001

Query  169   ATCAGACTGGTCTATGTTAGCAATAACCAGCTGATCAGGTGGTGTTAAGACGGGCTGTCC  228
             ||||||||||||||||||||||||||||||||||||||||||||||||||||||||||||
Sbjct  2000  ATCAGACTGGTCTATGTTAGCAATAACCAGCTGATCAGGTGGTGTTAAGACGGGCTGTCC  1941

Query  229   TCGTGTGAAGAACTTGTCAAAGTTCTCTGCTCCTTTGCCACACACTTTGGGCTTGAATGG  288
             ||||||||||||||||||||||||||||||||||||||||||||||||||||||||||||
Sbjct  1940  TCGTGTGAAGAACTTGTCAAAGTTCTCTGCTCCTTTGCCACACACTTTGGGCTTGAATGG  1881

Query  289   TGGCTGGATCTCCCTGTTCTCCAGTTTTTCCCAGTCGATCCTCCGGAAGAAGGCATGCTC  348
             ||||||||||||||||||||||||||||||||||||||||||||||||||||||||||||
Sbjct  1880  TGGCTGGATCTCCCTGTTCTCCAGTTTTTCCCAGTCGATCCTCCGGAAGAAGGCATGCTC  1821

Query  349   TCTCACGTCCCTCTCCCCCTCAGGCCCACAGCCCAGCCGCTTGGCTGGGTGTTTGGTCAT  408
             ||||||||||||||||||||||||||||||||||||||||||||||||||||||||||||
Sbjct  1820  TCTCACGTCCCTCTCCCCCTCAGGCCCACAGCCCAGCCGCTTGGCTGGGTGTTTGGTCAT  1761

Query  409   CAGTCCTTTGCAGATAGAAACAGCCTCCTTGGACAAGGATTTTGGATAGGAAACGTTGTG  468
             ||||||||||||||||||||||||||||||||||||||||||||||||||||||||||||
Sbjct  1760  CAGTCCTTTGCAGATAGAAACAGCCTCCTTGGACAAGGATTTTGGATAGGAAACGTTGTG  1701

Query  469   CTCCATGATAGACTGAAATAGCTCGTCTTCATCTTCACCATCAAATGGAGGCTGCCCGGC  528
             ||||||||||||||||||||||||||||||||||||||||||||||||||||||||||||
Sbjct  1700  CTCCATGATAGACTGAAATAGCTCGTCTTCATCTTCACCATCAAATGGAGGCTGCCCGGC  1641

Query  529   AAGCATTTCATACAACAGGACGCCATAGGCCCACCAGTCCACAGATTTTCCATACGGCTG  588
             ||||||||||||||||||||||||||||||||||||||||||||||||||||||||||||
Sbjct  1640  AAGCATTTCATACAACAGGACGCCATAGGCCCACCAGTCCACAGATTTTCCATACGGCTG  1581

Query  589   ATAAGCGATTATCTCTGGGGCGATATAATCTGGAGTCCCACAGAAGGTCCTGGTCGTGAC  648
             ||||||||||||||||||||||||||||||||||||||||||||||||||||||||||||
Sbjct  1580  ATAAGCGATTATCTCTGGGGCGATATAATCTGGAGTCCCACAGAAGGTCCTGGTCGTGAC  1521

Query  649   TCCATCCATCATGTGTTCCTTGCACATCCCAAAGTCAGCAATTTTGATATGTCCTTCTGA  708
             ||||||||||||||||||||||||||||||||||||||||||||||||||||||||||||
Sbjct  1520  TCCATCCATCATGTGTTCCTTGCACATCCCAAAGTCAGCAATTTTGATATGTCCTTCTGA  1461

Query  709   ATCCAACATGACGTTATCTAACTTCAGATCCCTATAAATGATTCCTCTTTTATGAAGAAA  768
             ||||||||||||||||||||||||||||||||||||||||||||||||||||||||||||
Sbjct  1460  ATCCAACATGACGTTATCTAACTTCAGATCCCTATAAATGATTCCTCTTTTATGAAGAAA  1401

Query  769   GAACAATCCGATGGAAATCTCTGCCGCATAGA  800
             ||||||||||||||||||||||||||||||||
Sbjct  1400  GAACAATCCGATGGAAATCTCTGCCGCATAGA  1369
```

Supplementary Figure S35: The down sequence (1369 to 2060) of PKCα (1-672 aa) with a myc-tag (amino acids 1-672) (GenBank Accession No. NM_002737, nucleotides 45 to 2060). (**A**) The sequencing data was present using the BGHR primer as initiator. (**B**) The "Query" (109 to 800) of sequence was aligned and identified by the NCBI BLAST.

Supplementary Table S1: Primers and restriction enzyme (R.E.) sites for the full-length and truncated MZF-1, Elk-1 and PKCα constructs

No.	constructs	A.A.	Primer forward	R.E.	Primer reverse	R.E.	Nuccleotides (bp)	Figures*
1	MZF-1-c-Myc	1–485	GGAATTCCAATGAATGGTCCCCTTGTG	EcoRI	GGGGTACCCTCGGCGCTGTGGACGCG	KpnI	10781–12235	S4–S5
2	MZF-1-c-Myc	1–60	GGAATTCCAATGAATGGTCCCCTTGTG	EcoRI	CCCAAGCTTGGGGCTGGGGAGCAGAACGCG	HindIII	10781–10960	S6
3	MZF-1-c-Myc	1–72	GGAATTCCAATGAATGGTCCCCTTGTG	EcoRI	GGGGTACCCCCATCCTCGTCCGTGGGGTC	KpnI	10781–10996	S7
4	MZF-1-c-Myc	1–141	GGAATTCCAATGAATGGTCCCCTTGTG	EcoRI	GGGGTACCCCCGCCGCACTCGCTGCAC	KpnI	10781–11203	S8
5	MZF-1-c-Myc	73–485	GGAATTCCACCCTGCCGGGGTGTGGGCCCT	EcoRI	GGGGTACCCCCCTCGGCGCTGTGGACGCG	KpnI	10997–12235	S9–S10
6	MZF-1-c-Myc	60–72	GGAATTCCAAGCGATCTGAGGAGTGAAC<br>AGGACCCCACGGACGAGGAT<br>CCCAAGCTTGGG	EcoRI	CCCAAGCTTGGGATCCTCGTCC<br>GTGGGGTCCTGTTCACTCCTC<br>AGATCGCTTGGAATTCC	HindIII	10961–10996	S11
7	MZF-1-c-Myc	60–72 (Mutant)	GGAATTCCAAGCGCACTGAGGAGTGA<br>ACAGGCACCCACGGCAGAGG<br>CACCCAAGCTTGGG	EcoRI	CCCAAGCTTGGGTGCCTCTGCCGTGGG<br>TGCCTGTTCACTCCTCAG<br>TGCGCTTGGAATTCC	HindII	10961–10996	S12
8	MZF-1-c-Myc	1–72 (Mutant)	GGAATTCCAATGAATGGTCCCCTTGTG	EcoRI	CCCAAGCTTGGGTGCCTCTGCCGTGG<br>GTGCCTGTTCACTCCTCAGTGCGCT	HindIII	10781–10996	S13
9	FLAG-MZF-1	1–485	GGAATTCAATGAATGGTCCCCTTGTG	EcoRI	CCGCTCGAGCTCGGCGCTGTGGACGCG	XhoI	10781–12235	S14–S15
10	Elk-1-c-Myc	1–428	CGGGATCCATGGACCCATCTGTGACG	BamHI	CCCAAGCTTTGGCTTCTGGGGCCCTGG	HindIII	101–1384	S16–S17
11	Elk-1-c-Myc	1–86	CGGGATCCATGGACCCATCTGTGACG	BamHI	CCCAAGCTTCACAAACTTGTAGACGAA	HindIII	101–385	S18
12	Elk-1-c-Myc	87–144	GGAATTCCATCCTACCCTGAGGTCGCT	EcoRI	CCCAAGCTTACCGCCTGGGCCTGCCAT	HindIII	359–532	S19
13	Elk-1-c-Myc	87–325	GGAATTCCATCCTACCCTGAGGTCGCT	EcoRI	CCCAAGCTTCGGGCTGAGTGGAAGCTC	HindIII	359–1075	S20
14	Elk-1-c-Myc	87–428	GGAATTCCATCCTACCCTGAGGTCGCT	EcoRI	CCCAAGCTTTGGCTTCTGGGGCCCTGG	HindIII	359–1384	S21–S22
15	Elk-1-c-Myc	321–428	GGAATTCCACTTCCACTCAGCCCGAGC	EcoRI	CCCAAGCTTTGGCTTCTGGGGCCCTGG	HindIII	1061–1384	S23
16	Elk-1-c-Myc	145–428	CGGGATCCTTGGCACGCAGCAGCCGG	BamHI	CCCAAGCTTTGGCTTCTGGGGCCCTGG	HindIII	533–1384	S24–S25
17	Elk-1-c-Myc	145–157	GGAATTCCATTGGCACGCAGCAG<br>CCGGAACGAGTACATGCGC<br>TCGGGCAAGCTTGGG	EcoRI	CCCAAGCTTGCCCGAGCGCATGTACTC<br>GTTCCGGCTGCTGCGTGCCAATGGAATTCC	HindIII	1061–1099	S26
18	Elk-1-c-Myc	309–320	GGAATTCCACAGCCGCAGAAGGG<br>CCGGAAGCCCCGGGACCT<br>AGAGAAGCTTGGG	EcoRI	CCCAAGCTTCTCTAGGTCCCGGGGCT<br>TCCGGCCCTTCTGCGGCTGTGGAATTCC	HindIII	1025–1060	S27
19	Elk-1-c-Myc	145–157 (Mutant)	GGAATTCCATTGGCAGCAAGCAGCGCAA<br>ACGAGTACATGGCATCGGGCAAGCTTGGG	EcoRI	CCCAAGCTTGCCCGATGCCATGTACTCGT<br>TTGCGCTGCTTGCTGCCAATGGAATTCC	HindIII	1061–1099	S28
20	Elk-1-c-Myc	145–428 (Mutant)	GGAATTCCATTGGCAGCAAGCAGCG<br>CAAACGAGTACATGGCATCGGGCCTCTAT	EcoRI	CCCAAGCTTTGGCTTCTGGGGCCCTGG	HindIII	533–1384	S29–S30
21	FLAG-Elk-1	1–428	GGGGTACCCCAAATGGACCCATCTGTGACG	KpnI	CGGGATCCCGTCATGGCTTCTGGGGCC	BamHI	101–1384	S31–S32
22	PKCα	1–672	GGAATTCCAATGGCTGACGTTTTCCCGGGC	EcoRI	CCCAAGCTTTACTGCACTCTGTAAGATGGG	HindIII	45–2060	S33–S35

*The DNA sequencing of all genes or fragments are completed by Life Technologies (Taipei, Taiwan) and the images of the data are archived by Chromas (Technelysium software for DNA sequence (Figure S5–S37), South Brisbane QLD, Australia) and the gene sequence is identified by NCBI (http://blast.ncbi.nlm.nih.gov/Blast.cgi) as followings. A.A., amino acids.
